# Chlorination of phenylallene derivatives with 1-chloro-1,2-benziodoxol-3-one: synthesis of *vicinal*-dichlorides and chlorodienes

**DOI:** 10.3762/bjoc.14.67

**Published:** 2018-04-09

**Authors:** Zhensheng Zhao, Graham K Murphy

**Affiliations:** 1 Department of Chemistry, University of Waterloo, 200 University Ave. W., Waterloo, N2L3G1, ON, Canada

**Keywords:** allene, chlorination, hypervalent iodine, synthetic methods, vinyl chloride

## Abstract

Allyl and vinyl chlorides represent important structural motifs in organic chemistry. Herein is described the chemoselective and regioselective reaction of aryl- and α-substituted phenylallenes with the hypervalent iodine (HVI) reagent 1-chloro-1,2-benziodoxol-3-one. The reaction typically results in *vicinal* dichlorides, except with proton-containing α-alkyl substituents, which instead give chlorinated dienes as the major product. Experimental evidence suggests that a radical mechanism is involved.

## Introduction

Organochlorine compounds are vital as polymer precursors [1], as pharmaceuticals [2–3] and agrochemicals [4–6] and as functional materials [7–8]. And as there is an abundance of chlorine-containing natural products, the synthesis of chlorinated functional groups, such as allyl- and vinyl chlorides, can represent challenging obstacles that practitioners of natural product synthesis must surmount [9–12]. More commonly, allyl- and vinyl chlorides are highly sought-after intermediates for effecting allylations, and for use in transition metal-catalyzed carbon–carbon and carbon–heteroatom bond-forming reactions [13–28]. Given the versatility of allyl chloride and β-chlorostyrene groups, installing them in close proximity (as shown in **3**) provides two handles for rapidly achieving high-density molecular complexity. Thus, the development of strategies for their synthesis is an important endeavour. We envisioned accomplishing this by developing a chemo- and regioselective *vicinal*-dichlorination of phenylallenes; however, no such chlorination reaction has yet been achieved [29–34].

**Scheme 1:**
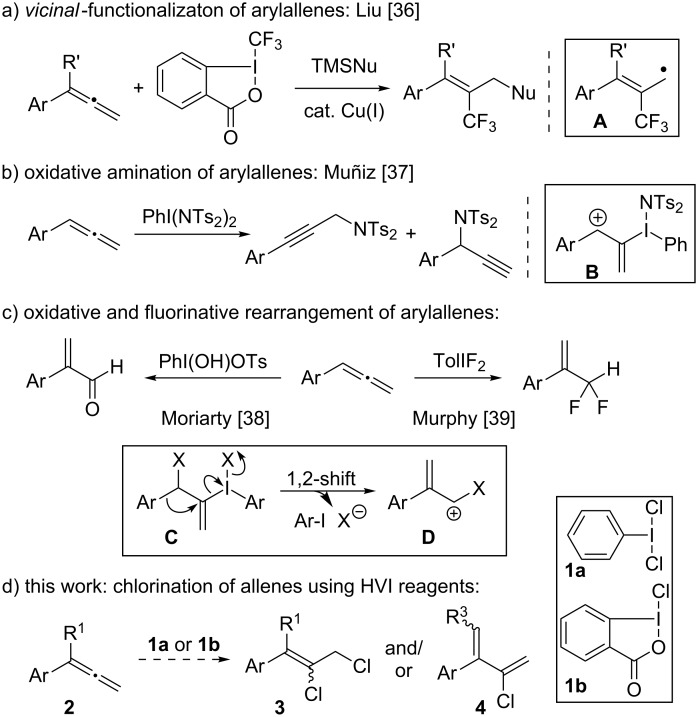
Reactions of substituted allenes with HVI reagents.

Recent reports of reactions between hypervalent iodine reagents and phenylallenes have highlighted the possible product outcomes achievable through ionic and radical reaction pathways. For example, Liu and co-workers used Togni’s benziodoxolone reagent [35] in a radical-mediated *vicinal* 2,3-difunctionalization of allenes, which proceeded via CF_3_-radical adduct **A** (Scheme 1a) [36]. In contrast, Muñiz reported that with PhI(NTs_2_)_2_, an oxidative amination occurred via cation **B**, giving regioisomeric propargylamides upon elimination of the iodanyl adduct (Scheme 1b) [37]. Moriarty and Murphy, respectively, showed how reactions of arylallenes with either PhI(OH)OTs [38] or TolIF_2_ [39] provide α-disubstituted styrenes by sequences involving intermediates analogous to **B**, followed by a 1,2-phenyl shift (**C** to **D**, Scheme 1c). There has been no investigation of the chemistry between arylallenes and chlorinated hypervalent iodine reagents, and given the differing reactivities that might be achievable with (dichloroiodo)benzene [40] (**1a**) and chlorobenziodoxolone (**1b**) [30,41–45], there is potential for the selective generation of diverse, poly-chlorinated scaffolds. Reported here are the results of these investigations, and while indiscriminate chlorination of **2** was observed with **1a**, **1b** reacted chemo- and regioselectively to give 2,3-dichlorides (**3**) or chlorodienes (**4**).

## Results and Discussion

**Scheme 2:**
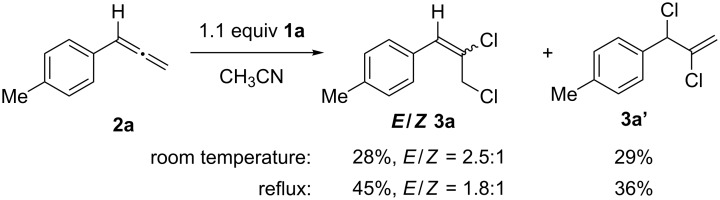
Chlorination of *p*-tolylallene (**2a**) with (dichloroiodo)benzene (**1a**).

We began our investigation of allene chlorination using *p*-tolylallene (**2a**), prepared from 4-methylstyrene through Doering–Moore–Skattebøl reaction [46], and iodane **1a**. The reaction was carried out using a slight excess of **1a** in acetonitrile, at both room temperature and at reflux, and upon consumption of the allene an inseparable mixture of chlorination products **3a** and **3a’** were obtained. While the overall yield of the chlorinated products increased when under reflux conditions, very little change in chemoselectivity was observed (Scheme 2) [31]. As these results were consistent with those achievable by other allene chlorination reactions, it was not investigated further.

We next investigated the chlorination of **2a** with benziodoxolone **1b** [47–49], which proved highly regioselective. An initial reaction with 2.2 equiv of **1b** in acetonitrile at room temperature failed; however, repeating the reaction under reflux conditions gave **3a** as a mixture of *E/Z* alkenes in 58% yield (Table 1, entries 1 and 2). The reaction was entirely selective for the terminal alkene, with none of **3a’** being observed. Toluene, chlorobenzene, DMF and DCE were also tested as reaction solvents, but none were superior to acetonitrile (Table 1, entries 3–6). A small improvement in yield was achieved by adding **2a** dropwise over 30 minutes (Table 1, entry 7), and we ultimately found that adding **2a** dropwise over one hour was optimal, giving **3a** in 90% yield as a *E*:*Z =* 1:1.25 mixture (Table 1, entry 8). One final reaction was carried out using the related *gem*-dimethyl chlorobenziodoxole [49], but the yield of **3a** decreased to 45% (Table 1, entry 9). This result is the first example of a selective chlorination reaction of phenylallenes, and as the regiochemical outcome parallels that observed by Liu (Scheme 1a), it is likely that radical pathways are involved [50].

**Table 1:** Optimization of the reaction conditions.^a^

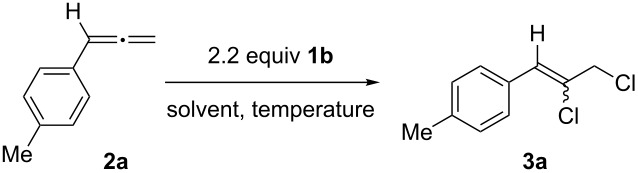			

entry	solvent	temp. °C	yield (%)

1	CH_3_CN	rt	trace
2	CH_3_CN	85	58%
3	toluene	110	40%^b^
4	PhCl	110	trace
5	DMF	110	trace
6	DCE	85	NR
7	CH_3_CN^c^	85	65%
8	CH_3_CN^d^	85	90%
9	CH_3_CN^e^	85	45%

^a^General conditions: Allene **2a** (0.2 mmol, 1 equiv), **1b** (0.44 mmol, 2.2 equiv) in 0.1 M solvent under reflux conditions for 2 h; isolated yield. ^b 1^H NMR yield using HMDSO (hexamethyldisiloxane) as internal standard. ^c^Dropwise addition of **2a** over 30 min. ^d^Dropwise addition of **2a** over 1 h. ^e^3,3-Dimethyl-1-chloro-1,2-benziodoxole used instead of **1b**.

**Scheme 3:**
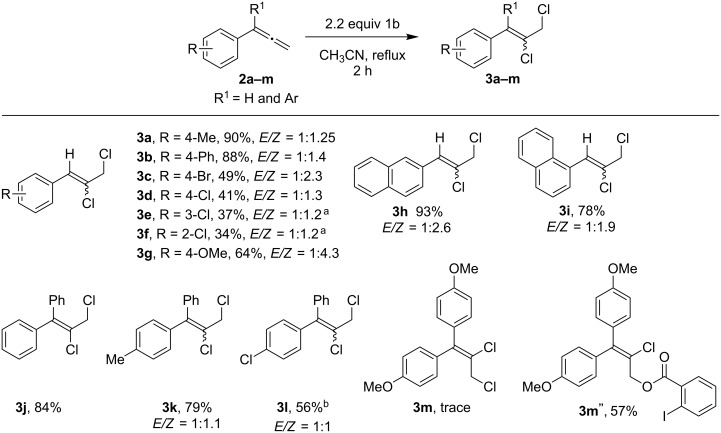
Chlorination of various aryl-substituted allenes. General conditions: Allene **2a** (0.2 mmol, 1 equiv) was added dropwise over 1 h to a solution of **1b** (0.44 mmol, 2.2 equiv) in 0.1 M CH_3_CN under reflux conditions, and the reaction stirred for 1 h; isolated yields. ^a 1^H NMR yield using HMDSO as internal standard.

A series of aryl- and allenyl-substituted phenylallenes (**2b–v**) were then examined in the chlorination reaction. First, phenylallenes with various aryl substituents were investigated, and the *p*-tolyl and *p*-biphenyl derivatives gave the 1,2-dichlorides **3a** and **3b** in excellent yield, favouring the *Z*-alkene (Scheme 3). The 4-bromo and 2-, 3- or 4-chloro derivatives **2c**–**f** led to **3c**–**f** in only moderate yield, with the mass balance of chlorinated materials being made up by the regioisomeric *vicinal*-dichloroination products **3c’**–**f’** (compare with **3a’**, Scheme 2) [30]. The *p*-anisyl derivative **2g** was also viable in the reaction, giving **3g** in 64% yield, as were the 1- and 2-naphthylallenes (**2h** and **2i**), which gave the desired dichlorides **3h** and **3i** in 93% and 78% yield. In each case, preference for forming the *Z*-alkene was observed, with selectivities ranging from 1.2–4.3:1 *Z*:*E*. α-Substituents on the allenes were equally viable, as 1,1-diphenylallene gave **3j** in 84% yield, and the related mono-methyl and mono-chloro derivatives **2k** and **2l** gave dichlorides **3k** and **3l** in 79% and 56% yield, respectively, with little preference observed for formation of either the *Z* or *E* alkene. Curiously, with 1,1-di(*p*-anisyl)allene (**2m**), only a trace of **3m** was observed, and the reaction instead produced iodobenzoate **3m”** in 57% yield. Presumably, this anomalous result arose due to the increased stability offered to an electron-deficient radical intermediate by the two methoxy groups, permitting a deviation in reaction outcome.

**Scheme 4:**
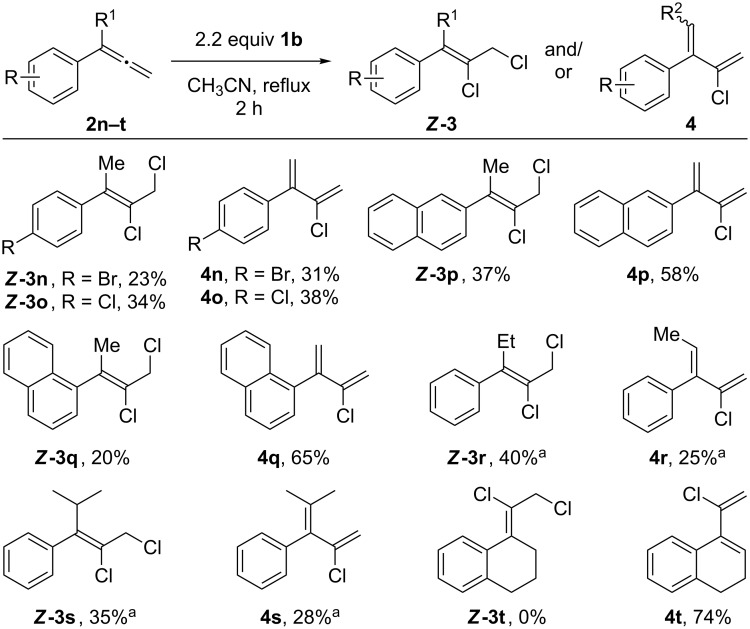
Chlorination of various α-substituted phenylallene derivatives. General conditions: Allene **2a** (0.2 mmol, 1 equiv) was added dropwise over 1 h to a solution of **1b** (0.44 mmol, 2.2 equiv) in 0.1 M CH_3_CN under reflux conditions, and the reaction stirred for 1 h; isolated yields. ^a 1^H NMR yield using HMDSO as internal standard.

We next subjected α-alkyl-substituted phenylallenes **2n–v** to the standard reaction conditions, which resulted in mixtures of exclusively the *Z*-dichloroalkenes (***Z*-3**) and chlorodienes **4** (Scheme 4). When the *para*-Br and *para*-Cl phenylallenes bearing α-methyl groups (**2n**, **2o**) were tested, the chlorodienes **4n** and **4o** were obtained in 31% and 38% yield, respectively, along with 23% of ***Z*-3n** and 34% of ***Z*-3o**. With α-methyl naphthylallene derivatives **2p** and **2q**, chlorodienes **4p** and **4q** were obtained in 58% and 65% yield, along with 37% and 20% of the 2,3-dichlorides. Substrates with α-ethyl (**2r**) and α-isopropyl (**2s**) substituents reacted similarly, giving chlorinated products in 63–65% yield. Lastly, vinylidene **2t** gave chlorodiene **4t** as the sole product in 74% yield.

**Scheme 5:**
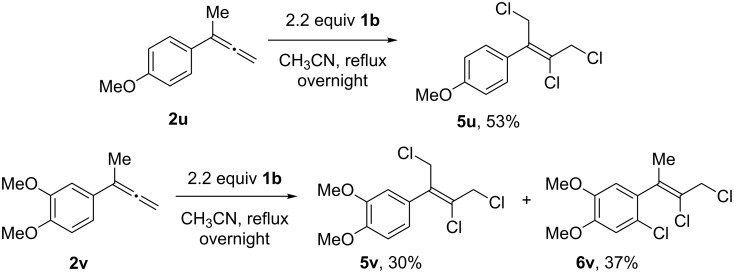
Chlorination of methoxy-substituted α-methyl phenylallenes. General conditions: Allene **2a** (0.2 mmol, 1 equiv) was added dropwise over 1 h to a solution of **1b** (0.44 mmol, 2.2 equiv) in 0.1 M CH_3_CN under reflux conditions, and the reaction stirred overnight; isolated yields.

As with alkoxy substrate **2m**, the α-methylated substrates **2u** and **2v** possessing methoxy group(s) on the arene also deviated from the expected reaction course. These reactions failed to fully consume the starting materials **2u** and **2v**, even upon prolonged heating, which we discovered to be the result of **1b** being also consumed through over-chlorination. 4-Methoxy derivative **2u** gave trichloride **5u** in 53% yield, with no trace of the expected dichloride **3u** or chlorodiene **4u** products observable by NMR (Scheme 5). The 3,4-dimethoxy substrate **2v** gave trichlorides **5v** and **6v** in a combined 67% yield, or in 91% yield based on the loading of **1b** (Scheme 5). These anomalous outcomes were again rationalized as resulting from the stabilization of radical intermediates gained upon methoxy substitution [51], which permitted further chlorination of either the methyl or arene groups.

**Scheme 6:**
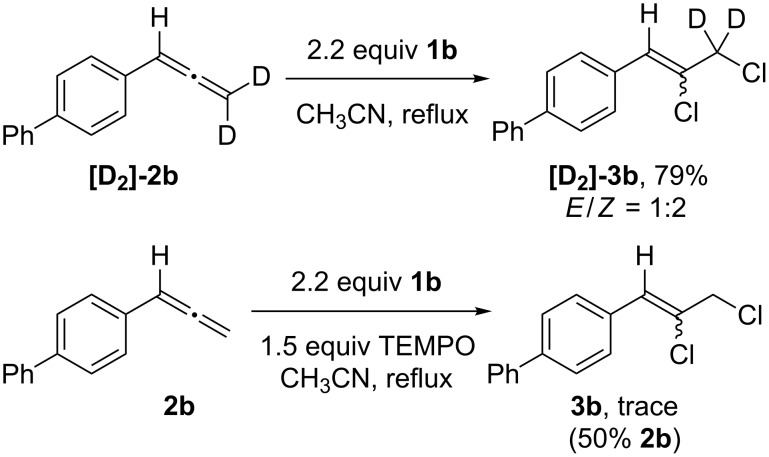
Control reactions: (a) chlorination of deuterated biphenylallene **[D_2_]-2b**; (b) reaction with TEMPO.

To gain insight into the reaction mechanism we carried out two key control experiments. First, to test for rearrangement processes that might not be elucidated through product analysis alone, deuterated biphenylallene **[D_2_]-2b** was subjected to the standard reaction conditions, and **[D_2_]-3b** (*E/Z* = 1:2) was obtained in 79% yield (Scheme 6 top, also see Supporting Information File 1). As there was no indication of deuterium scrambling observable by ^1^H or ^2^H NMR of the product mixture, it appeared that 1,2-phenyl shifts or other rearrangement processes were not involved in the reaction. A further reaction was carried out in the presence of the radical scavenger TEMPO (1.5 equiv), from which only a trace of **3b** was recovered, along with 50% of **2b** (Scheme 6 bottom). As the chlorination reactivity was suppressed, our hypothesis that these reactions involved radical intermediates was further supported.

**Figure 1:**
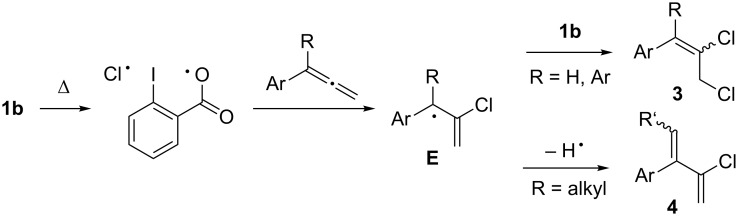
Proposed reaction mechanism.

When allene chlorination was carried out with **1a**, the observed product distributions were consistent with the results previously obtained, suggesting that ionic processes were operative. Furthermore, since no evidence of propargyl chlorides or α-dichloromethylstyrenes were observed, it appears the chlorination of allenes with **1a** proceeded without interruption of 1,2-phenyl shifts or iodane elimination, resulting in a reactivity pattern that differs from the related reagents TolIF_2_, PhI(OH)OTs or PhI(NTs_2_)_2_ (Scheme 1b and c). With **1b**, however, the reactions were entirely selective for 2,3-dichlorination of the allene, which was consistent with the regiochemical outcome of reactions involving a trifluoromethyl radical (Scheme 1a). This, coupled with the results of Scheme 6, led us to propose a radical mechanism that was initiated by homolytic cleavage of the I–Cl bond of **1b** at elevated temperature (Figure 1) [50]. Addition of the chlorine atom to the allene central carbon resulted in the highly stabilized radical intermediate **E**, which then abstracted a chlorine atom from a second equivalent of **1b**, giving dichlorides **3**. Or, in the case of α-alkyl groups, intermediate **E** was also subject to a competing hydrogen abstraction pathway, resulting in mixtures of **3** and chlorodienes **4**.

## Conclusion

In conclusion, we report here an efficient new process for the chlorination of substituted phenylallene derivatives using the hypervalent iodine reagent 1-chloro-1,2-benziodoxol-3-one (**1b**). The reactions disclosed here represent the first report of a regioselective chlorination of phenylallenes, in which the 2,3-allene olefin undergoes selective *vicinal* dichlorination. Overall, the reactions were mild and operationally-simple, tolerant to a variety of different functional groups, and provided the products in typically good yield. The selectivity of the reaction is presumably derived from it being a radical, not ionic, process, which also enabled the formation of chlorodiene products with α-alkyl substituted allenes. This reaction offers a new strategy for accessing dichlorinated functional group building blocks not readily accessible with other reagents, and our continued work in this area will be disclosed in due course.

## Supporting Information

File 1 Experimental and characterization details, and NMR spectra of compounds.
