# Supplementary material for: Chlorination of phenylallene derivatives with 1-chloro-1,2-benziodoxol-3-one: synthesis of vicinal-dichlorides and chlorodienes
Source: Beilstein J Org Chem. 2018 Apr 9;14:796–802. doi: 10.3762/bjoc.14.67 (PMC5905278; doi:10.3762/bjoc.14.67)
Supplement: File 1 — Experimental and characterization details, and NMR spectra of compounds. [file Beilstein_J_Org_Chem-14-796-s001.pdf]

Supporting Information

for

Chlorination of phenylallene derivatives with 1-chloro-1,2-benziodoxol-3-one: synthesis of *vicinal*-dichlorides and chlorodienes

Zhensheng Zhao and Graham K. Murphy*

Address: Department of Chemistry, University of Waterloo, 200 University Ave. W., Waterloo, N2L3G1, ON, Canada

Email: Graham Murphy - graham.murphy@uwaterloo.ca

* Corresponding author

Experimental and characterization details, and NMR spectra of compounds

Table of Contents

General experimental and characterization details: S2
General procedure for allene synthesis: S2
Characterization data for allenes 2a–v: S3
General procedure for chlorination of allenes: S11
Characterization data for chloroination: S12
Synthesis of 4-(propa-1,2-dien-1-yl-3,3-d_2)-1,1'-biphenyl) ([D_2]-2b): S18
Synthesis of 4-(2,3-dichloroprop-1-en-1-yl-3,3-d_2)-1,1'-biphenyl ([D_2]-3b): S19
^{1}H NMR, ^{19}F and ^{13}C NMR spectra: S20

General experimental and characterization details:

Reactions were carried out in oven-dried glassware under a nitrogen atmosphere. Solvents were dried and purified using a JC Meyer solvent purification system, and were used without further purification. Transfer of anhydrous solvents and reagents was accomplished with oven-dried syringes. Thin-layer chromatography was performed on glass plates pre-coated with 0.25 mm silica gel 60 F254 (Silicycle). Flash chromatography columns were packed with 230–400 mesh silica gel (Silicycle). Radial chromatography was carried out on a Chromatotron 7924T (Harrison Research) equipped with 4 mm silica gel 60 F254 with gypsum binder (EM) thick-layer plates on glass rotors. Infrared spectra were recorded on a Perkin Elmer FT-IR Spectrum Two with ATR Two. Proton NMR spectra (^{1}H NMR) were recorded at 300 or 500 MHz, and are reported (ppm) relative to the residual chloroform peak (7.26 ppm), and coupling constants (J) are reported in hertz (Hz). Carbon NMR spectra (^{13}C NMR) were recorded at 125 or 75 MHz and are reported (ppm) relative to the center line of the triplet from $CDCl_3$ (77.16 ppm). High resolution mass spectroscopy was performed on a Thermo Fisher Scientific Q-Exactive hybrid mass spectrometer equipped with an Agilent HPLC pump interfaced with the Q-Exactive's APCI source.

General procedure for allene synthesis: [1]

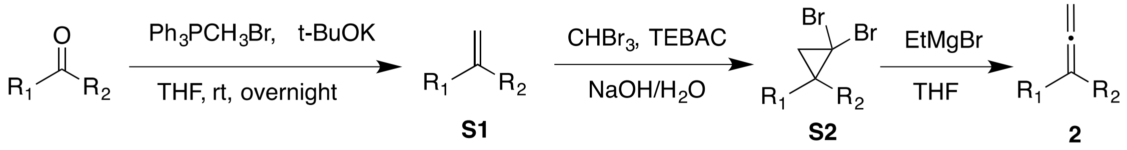

Step 1: In an oven dried flask, was added methyl triphenylphosphonium bromide (1.2 equiv) followed by THF (2.5 mL/mmol). Then *t*-BuOK (1.2 equiv) was added and the resulting yellow suspension was stirred at room temperature for 60 min. To this suspension, a solution of ketone or aldehyde (1.0 equiv) was added in one portion and the resulting mixture was further stirred at room temperature overnight. Water and DCM were added to the reaction mixture, and the aqueous phase was extracted with DCM (3 × 50 mL). The combined organic phases were washed with saturated NaCl solution, dried over Na_2SO_4 and the solvent removed under reduced pressure. The reaction mixture was purified by column chromatography over silica gel (200–300 mesh) using hexanes as eluent afforded **S1**.

Step 2: To a solution of alkene **S1** (1.0 equiv), bromoform (1.5 equiv) and $BnNEt_3Cl$ (1 mol %) was added dropwise a solution of 50% NaOH (4.0 equiv), and the mixture was stirred at room temperature for 60 min, then heated to 60 ^{o}C and further stirred until conversion was complete as observed by TLC analysis. Water and DCM were added and the aqueous phase was extracted with DCM (3 × 50 mL). The combined organic phases were washed with saturated NaCl solution, dried over Na_2SO_4 and the solvent removed under reduced pressure. The reaction mixture was purified by column chromatography afforded **S2**.

Step3: EtMgBr (3.0 M in ether, 1.5 equiv) was added dropwise to a pre-cooled (ice-bath) solution of **S2** (1.0 equiv) in dry THF (1.0 mL/mmol) under nitrogen atmosphere. After EtMgBr was added the mixture was then slowly warmed to room temperature, and stirred at room temperature for an additional 2 hours. Then the reaction was quenched by HCl (0.5 N, 10 mL) solution, water was added, and the mixture extracted with ether (3 × 50 mL). The combined organic layers were washed with brine, dried with anhydrous Na_2SO_4 and filtered. After removing the solvent under reduced pressure, the crude product was purified by column chromatography on silica gel to afford allenes **2a**–**v**.

Characterization data for allenes 2a–v:

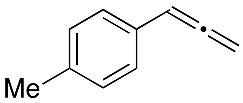

1-Methyl-4-(propa-1,2-dien-1-yl)benzene (2a): Obtained as a colorless liquid in 95% yield. Compound purified by silica gel column chromatography eluting with Hexanes. R_f=0.7 (Hexanes). ^{1}H NMR (300 MHz, $CDCl_3$) δ 7.27 (d, *J* = 8.0 Hz, 2H), 7.18 (d, *J* = 8.0 Hz, 2H), 6.22 (t, *J* = 6.8 Hz, 1H), 5.19 (d, *J* = 6.8 Hz, 2H), 2.40 (s, 3H). ^{13}C NMR (75 MHz, $CDCl_3$) δ 209.6, 136.6, 130.9, 129.3, 126.6, 93.7, 78.6, 21.1.

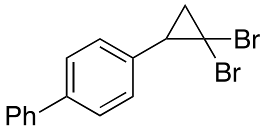

4-(2,2-Dibromocyclopropyl)-1,1'-biphenyl (S2-b): Obtained as a colorless liquid in 36% yield. Compound purified by silica gel column chromatography eluting with Hexanes. R_f=0.3 (Hexanes). ^{1}H NMR (300 MHz, $CDCl_3$) δ 7.61 – 7.56 (m, 4H), 7.46 – 7.40 (m, 2H), 7.37 – 7.30 (m, 3H), 2.98 (t, *J* = 9.4 Hz, 1H), 2.19 – 2.13 (m, 1H), 2.08 – 2.00 (m, 1H). ^{13}C NMR (75 MHz, $CDCl_3$) δ 140.4, 140.3, 134.9, 129.2, 128.7, 127.3, 126.9, 126.8, 35.6, 28.4, 27.3.

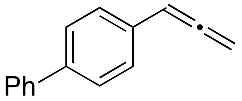

4-(Propa-1,2-dien-1-yl)-1,1'-biphenyl (2b):[2] Obtained as a yellow solid in 99% yield. Compound purified by silica gel column chromatography eluting with Hexanes. R_f=0.45 (Hexanes). ^{1}H NMR (300 MHz, $CDCl_3$) δ 7.59 (d, *J* = 7.3 Hz, 2H), 7.55 (d, *J* = 8.2 Hz, 2H), 7.43 (t, *J* = 7.5 Hz, 2H), 7.38 – 7.31 (m, 3H), 6.21 (t, *J* = 6.8 Hz, 1H), 5.18 (d, *J* = 6.8 Hz, 2H). ^{13}C NMR (75 MHz, $CDCl_3$) δ 209.9, 140.7, 139.6, 132.9, 128.7, 127.2, 127.1, 127.0, 126.8, 93.5, 78.8.

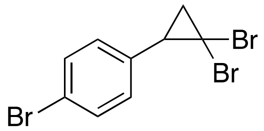

1**-Bromo-4-(2,2-dibromocyclopropyl)benzene (S2-c):** Obtained as a colorless liquid in 80% yield. Compound purified by silica gel column chromatography eluting with Hexanes. R_f=0.4 (Hexanes). ^{1}H NMR (300 MHz, $CDCl_3$) δ 7.47 (d, *J* = 8.4 Hz, 2H), 7.11 (d, *J* = 8.4 Hz, 2H), 2.98 – 2.82 (m, 1H), 2.13 (dd, *J* = 9.8, 7.2 Hz, 1H), 1.96 (t, *J* = 8.1 Hz, 1H). ^{13}C NMR (75 MHz, $CDCl_3$) δ 135.0, 131.4, 130.6, 121.6, 35.3, 27.8, 27.4.

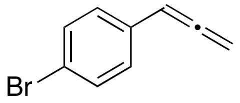

1-Bromo-4-(propa-1,2-dien-1-yl)benzene (2c):[3] Obtained as a colorless liquid in 71% yield. Compound purified by silica gel column chromatography eluting with Hexanes. R_f=0.55 (Hexanes). ^{1}H NMR (500 MHz, $CDCl_3$) δ 7.44 (d, *J* = 8.4 Hz, 2H), 7.20 – 7.14 (m, 2H), 6.12 (t, *J* = 6.8 Hz, 1H), 5.17 (d, *J* = 6.8 Hz, 2H). ^{13}C NMR (126 MHz, $CDCl_3$) δ 210.5, 133.7, 132.4, 128.9, 121.2, 93.9, 80.0.

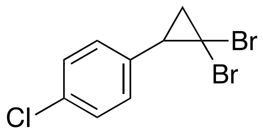

1**-Chloro-4-(2,2-dibromocyclopropyl)benzene (S2-d):** Obtained as a colorless liquid in 74% yield. Compound purified by silica gel column chromatography eluting with Hexanes. R_f=0.6 (Hexanes). ^{1}H NMR (300 MHz, $CDCl_3$) δ 7.32 (d, *J* = 8.5 Hz, 2H), 7.17 (d, *J* = 8.3 Hz, 2H), 2.90 (dd, *J* = 10.3, 8.4 Hz, 1H), 2.14

(dd, J = 10.5, 7.8 Hz, 1H), 1.96 (t, J = 8.0 Hz, 1H). ^{13}C NMR (75 MHz, $CDCl_3$) δ 134.5, 133.5, 130.2, 128.5, 77.0, 35.3, 27.8, 27.4.

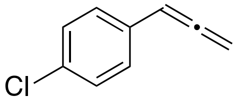

1-Chloro-4-(propa-1,2-dien-1-yl)benzene (2d):[4] Obtained as a colorless liquid in 73% yield. Compound purified by silica gel column chromatography eluting with Hexanes. R_f=0.7 (Hexanes). ^{1}H NMR (300 MHz, $CDCl_3$) δ 7.27 (d, J = 8.5 Hz, 2H), 7.21 (d, J = 8.4 Hz, 2H), 6.12 (t, J = 6.8 Hz, 1H), 5.15 (d, J = 6.8 Hz, 2H). ^{13}C NMR (75 MHz, $CDCl_3$) δ 209.7, 132.4, 132.4, 128.6, 127.8, 93.0, 79.1.

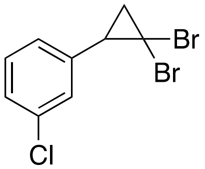

1-Chloro-3-(2,2-dibromocyclopropyl)benzene (S2-e): Obtained as a colorless liquid in 85% yield. Compound purified by silica gel column chromatography eluting with Hexanes. R_f=0.55 (Hexanes). ^{1}H NMR (300 MHz, $CDCl_3$) δ 7.29 (d, J = 4.9 Hz, 2H), 7.24 (s, 1H), 7.18 – 7.11 (m, 1H), 3.02 – 2.85 (m, 1H), 2.15 (dd, J = 10.4, 7.9 Hz, 1H), 1.99 (t, J = 8.1 Hz, 1H). ^{13}C NMR (75 MHz, $CDCl_3$) δ 138.0, 134.2, 129.6, 129.1, 127.9, 127.2, 35.5, 27.5, 27.4.

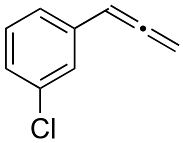

1-Chloro-3-(propa-1,2-dien-1-yl)benzene (2e):[3] Obtained as a colorless liquid in 75% yield. Compound purified by silica gel column chromatography eluting with Hexanes. R_f=0.7 (Hexanes). ^{1}H NMR (300 MHz, $CDCl_3$) δ 7.32 (s, 1H), 7.26 – 7.15 (m, 3H), 6.12 (t, J = 6.7 Hz, 1H), 5.20 (d, J = 6.7 Hz, 2H). ^{13}C NMR (75 MHz, $CDCl_3$) δ 209.9, 135.9, 134.5, 129.7, 126.8, 126.5, 124.8, 93.1, 79.2.

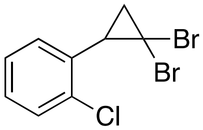

1-Chloro-2-(2,2-dibromocyclopropyl)benzene (S2-f): Obtained as a colorless liquid in 95% yield. Compound purified by silica gel column chromatography eluting with Hexanes. R_f=0.55 (Hexanes). ^{1}H NMR (300 MHz, $CDCl_3$) δ 7.47 (d, J = 7.5 Hz, 1H), 7.25 (p, J = 7.4 Hz, 2H), 7.05 (d, J = 7.3 Hz, 1H), 3.00 (t, J = 9.4 Hz, 1H), 2.18 (t, J = 9.1 Hz, 1H), 2.04 (t, J = 8.2 Hz, 1H). ^{13}C NMR (75 MHz, $CDCl_3$) δ 136.6, 135.0, 129.3, 129.2, 128.9, 126.6, 34.9, 27.2, 27.0.

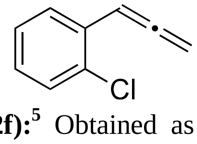

1-Chloro-2-(propa-1,2-dien-1-yl)benzene (2f):[5] Obtained as a colorless liquid in 60% yield. Compound purified by silica gel column chromatography eluting with Hexanes. R_f=0.7 (Hexanes). ^{1}H NMR (300 MHz, $CDCl_3$) δ 7.47 (d, J = 7.7 Hz, 1H), 7.33 (d, J = 7.9 Hz, 1H), 7.20 (t, J = 7.5 Hz, 1H), 7.11 (t, J = 7.6 Hz, 1H), 6.63 (t, J = 6.8 Hz, 1H), 5.18 (d, J = 6.9 Hz, 2H). ^{13}C NMR (75 MHz, $CDCl_3$) δ 210.4, 131.8, 131.6, 129.5, 128.1, 127.8, 126.7, 90.3, 78.8.

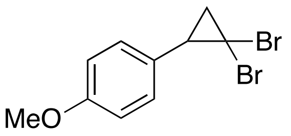

1-(2,2-Dibromocyclopropyl)-4-methoxybenzene (S2-g): Obtained as a colorless liquid in 63% yield. Compound purified by silica gel column chromatography eluting with Hexanes. R_f=0.35 (DCM: Hexanes= 15: 85). ^{1}H NMR (300 MHz, $CDCl_3$) δ 7.18 (d, J = 8.3 Hz, 2H), 6.89 (d, J = 8.3 Hz, 2H), 3.81 (s, 3H), 2.97 – 2.84 (m, 1H), 2.13 – 2.07 (m, 1H), 1.95 (t, J = 8.0 Hz, 1H). ^{13}C NMR (75 MHz, $CDCl_3$) δ 159.0, 129.9, 128.1, 113.6, 55.2, 35.2, 29.3, 27.2.

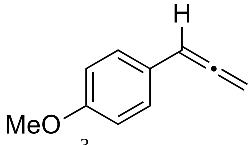

1-Methoxy-4-(propa-1,2-dien-1-yl)benzene (2g):[3] Obtained as a colorless liquid in 99% yield. Compound purified by silica gel column chromatography eluting with Hexanes. R_f=0.5 (DCM: Hexanes= 15: 85). ^{1}H NMR (300 MHz, $CDCl_3$) δ 7.24 (d, J = 8.6 Hz, 2H), 6.87 (d, J = 8.7 Hz, 2H), 6.15 (t, J = 6.8 Hz, 1H), 5.13 (d, J = 6.8 Hz, 2H), 3.80 (s, 4H). ^{13}C NMR (75 MHz, $CDCl_3$) δ 209.3, 158.7, 127.7, 126.0, 114.1, 93.3, 78.6, 55.2.

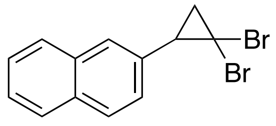

2-(2,2-Dibromocyclopropyl)naphthalene (S2-h): Obtained as a colorless liquid in 66% yield. Compound purified by silica gel column chromatography eluting with Hexanes. R_f=0.30 (Hexanes). ^{1}H NMR (300 MHz, $CDCl_3$) δ 7. 85 – 7.80 (m, 3H), 7.65 (s, 1H), 7.53 – 7.39 (m, 3H), 3.27 – 2.99 (m, 1H), 2.32 – 2.06 (m, 2H). ^{13}C NMR (75 MHz, $CDCl_3$) δ 133.5, 133.1, 132.6, 127.8, 127.7, 127.6, 127.5, 126.9, 126.2, 126.0, 36.0, 28.3, 27.3.

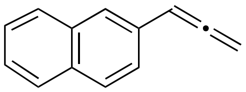

2-(Propa-1,2-dien-1-yl)naphthalene (2h):[3] Obtained as a colorless liquid in 76% yield. Compound purified by silica gel column chromatography eluting with Hexanes. R_f=0.45 (Hexanes). ^{1}H NMR (300 MHz, $CDCl_3$) δ 7. 78 – 7.75 (m, 3H), 7.65 (s, 1H), 7.50 (d, J = 8.6 Hz, 1H), 7. 45 – 7.41 (m, 2H), 6.34 (t, J = 6.8 Hz, 1H), 5.22 (d, J = 6.7 Hz, 2H).^{13}C NMR (75 MHz, $CDCl_3$) δ 210.2, 133.6, 132.5, 131.3, 128.2, 127.6, 127.5, 126.1, 125.5, 125.3, 124.5, 94.2, 79.0.

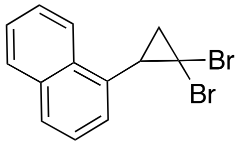

1-**(2,2-Dibromocyclopropyl)naphthalene (S2-i):** Obtained as a colorless liquid in 55% yield. Compound purified by silica gel column chromatography eluting with Hexanes. R_f=0.45 (Hexanes). ^{1}H NMR (300 MHz, $CDCl_3$) δ 8.29 (d, J = 8.4 Hz, 1H), 7.94 (d, J = 8.1 Hz, 1H), 7.86 (d, J = 8.3 Hz, 1H), 7.70 (t, J = 7.6 Hz, 1H), 7.59 (t, J = 7.5 Hz, 1H), 7.44 (t, J = 7.7 Hz, 1H), 7.22 (d, J = 7.1 Hz, 1H), 3.30 (t, J = 7.9 Hz, 1H), 2.32 (t, J = 8.0 Hz, 1H), 2.22 (t, J = 7.9 Hz, 1H). ^{13}C NMR (75 MHz, $CDCl_3$) δ 133.8, 133.5, 133.2, 128.6, 128.5, 126.8, 126.3, 125.7, 125.3, 124.8, 34.8, 28.5, 27.0.

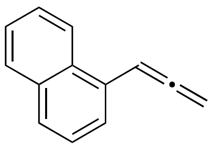

1-(propa-1,2-dien-1-yl)naphthalene (2i):[6] Obtained as a yellow oil in 90% yield. Compound purified by silica gel column chromatography eluting with Hexanes. R_f=0.55 (Hexanes). ^{1}H NMR (300 MHz, $CDCl_3$) δ 8.28 (d, *J* = 9.1 Hz, 1H), 7.92 (d, *J* = 7.0 Hz, 1H), 7.81 (d, *J* = 8.1 Hz, 1H), 7.68 (d, *J* = 7.1 Hz, 1H), 7.63 – 7.48 (m, 3H), 6.95 (t, *J* = 6.9 Hz, 1H), 5.27 (d, *J* = 6.9 Hz, 2H). ^{13}C NMR (75 MHz, $CDCl_3$) δ 211.0, 133.9, 130.8, 130.1, 128.7, 127.5, 126.0, 125.7, 125.6, 125.3, 123.5, 90.5, 77.8.

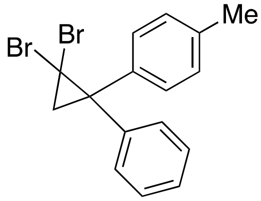

1-(2,2-Dibromo-1-phenylcyclopropyl)-4-methylbenzene (S2-j): Obtained as a colorless liquid in 59% yield. Compound purified by silica gel column chromatography eluting with Hexanes. R_f=0.35 (Hexanes). ^{1}H NMR (300 MHz, $CDCl_3$) δ 7.53 (d, *J* = 7.1 Hz, 2H), 7.43 (d, *J* = 8.1 Hz, 2H), 7.33 (t, *J* = 7.8 Hz, 2H), 7.25 (d, *J* = 5.9 Hz, 1H), 7.15 (d, *J* = 7.9 Hz, 2H), 2.47 (s, 2H), 2.32 (s, 3H). ^{13}C NMR (75 MHz, $CDCl_3$) δ 142.0, 139.0, 137.0, 129.1, 129.1, 129.0, 128.4, 127.2, 44.8, 34.4, 34.2, 21.1.

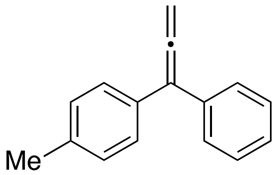

1-Methyl-4-(1-phenylpropa-1,2-dien-1-yl)benzene (2j)[7]: Obtained as a colorless oil in 95% yield. Compound purified by silica gel column chromatography eluting with Hexanes. R_f=0.45 (Hexanes). ^{1}H NMR (300 MHz, $CDCl_3$) δ 7.44 – 7.33 (m, 4H), 7.30 (dd, *J* = 7.5, 3.4 Hz, 3H), 7.19 (d, *J* = 7.9 Hz, 2H), 5.27 (s, 2H), 2.39 (s, 3H). ^{13}C NMR (75 MHz, $CDCl_3$) δ 209.7, 136.9, 136.4, 133.2, 129.1, 128.3, 128.3, 128.3, 127.1, 108.9, 77.8, 21.1.

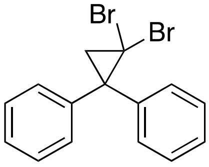

(2,2-Dibromocyclopropane-1,1-diyl)dibenzene (S2-k): Obtained as a colorless liquid in 54% yield. Compound purified by silica gel column chromatography eluting with dichloromethane and Hexanes. R_f=0.3 (DCM: Hexanes= 15:85). ^{1}H NMR (300 MHz, $CDCl_3$) δ 7.54 (d, *J* = 7.6 Hz, 4H), 7.34 (t, *J* = 7.4 Hz, 4H), 7.25 (t, *J* = 7.3 Hz, 2H), 2.50 (s, 2H). ^{13}C NMR (75 MHz, $CDCl_3$) δ 141.8, 129.1, 128.4, 127.3, 45.0, 34.5, 33.9.

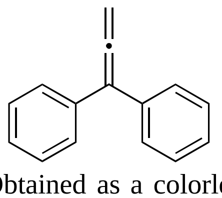

Propa-1,2-diene-1,1-diyldibenzene (2k):[6] Obtained as a colorless oil in 78% yield. Compound purified by silica gel column chromatography eluting with dichloromethane and Hexanes. R_f=0.4 (DCM: Hexanes= 15:85).

^{1}H NMR (300 MHz, $CDCl_3$) δ 7.50 – 7.31 (m, 10H), 5.34 (s, 2H). ^{13}C NMR (75 MHz, $CDCl_3$) δ 210.0, 136.3, 128.5, 127.3, 109.3, 78.2.

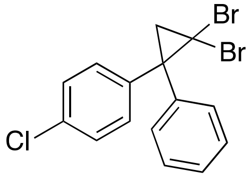

1-Chloro-4-(2,2-dibromo-1-phenylcyclopropyl)benzene (S2-l): Obtained as a yellow oil in 59% yield. Compound purified by silica gel column chromatography eluting with Hexanes. R_f=0.5 (Hexanes). ^{1}H NMR (300 MHz, $CDCl_3$) δ 7.47 (dd, J = 10.9, 8.4 Hz, 4H), 7.35-7.24 (m, 5H), 2.49 (d, J = 7.6 Hz, 1H), 2.43 (d, J = 7.6 Hz, 1H).

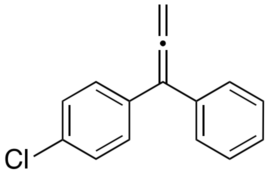

1-Chloro-4-(1-phenylpropa-1,2-dien-1-yl)benzene (2l):[6] Obtained as a yellow oil in 30% yield. Compound purified by silica gel column chromatography eluting with Hexanes. R_f=0.65 (Hexanes). ^{1}H NMR (300 MHz, $CDCl_3$) δ 7.37-7.35 (m, 4H), 7.31-7.29 (m, 5H), 5.29 (s, 2H). ^{13}C NMR (75 MHz, $CDCl_3$) δ 209.7, 135.7, 134.8, 132.9, 129.6, 128.5, 128.4, 128.3, 127.3, 108.3, 78.3.

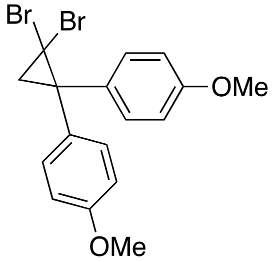

4,4'-(2,2-Dibromocyclopropane-1,1-diyl)bis(methoxybenzene) (S2-m) : Obtained as a yellow oil in 32% yield. Compound purified by silica gel column chromatography eluting with EtOAc and Hexanes. R_f=0.30 (EtOAc: Hexanes= 1:10). ^{1}H NMR (300 MHz, $CDCl_3$) δ 7.37 (d, J = 8.8 Hz, 4H), 6.82 (d, J = 8.7 Hz, 4H), 3.74 (s, 6H), 2.38 (s, 2H). ^{13}C NMR (75 MHz, $CDCl_3$) δ 158.5, 134.4, 130.0, 113.7, 55.1, 43.7, 34.5.

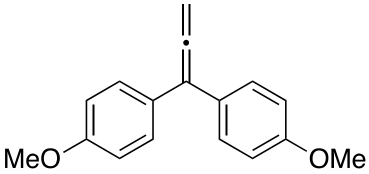

4,4'-(Propa-1,2-diene-1,1-diyl)bis(methoxybenzene) (2m):[8] Obtained as a yellow oil in 69% yield. Compound purified by silica gel column chromatography eluting with EtOAc and Hexanes. R_f=0.40 (EtOAc: Hexanes= 1:10). ^{1}H NMR (300 MHz, $CDCl_3$) δ 7.32 (d, J = 8.3 Hz, 4H), 6.92 (d, J = 8.3 Hz, 4H), 5.25 (s, 2H), 3.84 (s, 6H). ^{13}C NMR (75 MHz, $CDCl_3$) δ 209.4, 158.8, 129.4, 128.6, 113.8, 108.1, 77.7, 55.2.

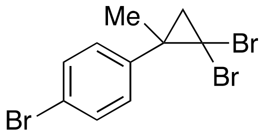

1-Bromo-4-(2,2-dibromo-1-methylcyclopropyl)benzene (S2-n) : Obtained as a colorless liquid in 67% yield. Compound purified by silica gel column chromatography eluting with Hexanes. R_f=0.55 (Hexanes). ^{1}H NMR (300 MHz, $CDCl_3$) δ 7.48 (d, *J* = 8.4 Hz, 2H), 7.18 (d, *J* = 8.4 Hz, 2H), 2.18 (d, *J* = 7.6 Hz, 1H), 1.78 (d, *J* = 7.6 Hz, 1H), 1.69 (s, 3H). ^{13}C NMR (75 MHz, $CDCl_3$) δ 141.3, 131.5, 130.2, 121.2, 36.0, 35.2, 33.7, 27.5.

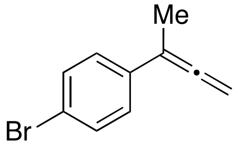

1-Bromo-4-(buta-2,3-dien-2-yl)benzene (2n):[6] Obtained as a yellow solid in 71% yield. Compound purified by silica gel column chromatography eluting with Hexanes. R_f=0.7 (Hexanes). ^{1}H NMR (300 MHz, $CDCl_3$) δ 7.42 (d, *J* = 8.5 Hz, 2H), 7.25 (d, *J* = 8.4 Hz, 2H), 5.01 (d, *J* = 2.9 Hz, 2H), 2.06 (t, *J* = 2.8 Hz, 3H). ^{13}C NMR (75 MHz, $CDCl_3$) δ 208.8, 135.7, 131.2, 127.2, 120.3, 99.0, 77.2, 16.5.

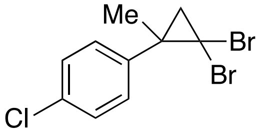

1-Chloro-4-(2,2-dibromo-1-methylcyclopropyl)benzene (S2-o):[6] Obtained as a colorless liquid in 99% yield. Compound purified by silica gel column chromatography eluting with Hexanes. R_f=0.35 (Hexanes). ^{1}H NMR (300 MHz, $CDCl_3$) δ 7.32 (d, *J* = 8.5 Hz, 2H), 7.22 (d, *J* = 7.9 Hz, 2H), 2.11 (d, *J* = 7.6 Hz, 1H), 1.77 (d, *J* = 7.6 Hz, 1H), 1.68 (s, 3H). ^{13}C NMR (75 MHz, $CDCl_3$) δ 140.8, 133.0, 129.8, 128.5, 35.9, 35.1, 33.7, 27.5.

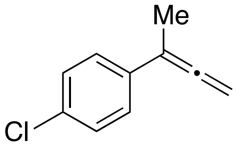

1-(Buta-2,3-dien-2-yl)-4-chlorobenzene (2o):[6] Obtained as a yellow solid in 86% yield. Compound purified by silica gel column chromatography eluting with Hexanes. R_f=0.45 (Hexanes). ^{1}H NMR (300 MHz, $CDCl_3$) δ 7.32 (d, *J* = 8.8 Hz, 2H), 7.27 (d, *J* = 8.9 Hz, 2H), 5.03 (s, 2H), 2.06 (s, 3H). ^{13}C NMR (75 MHz, $CDCl_3$) δ 208.8, 135.2, 132.2, 128.3, 126.8, 98.9, 77.2, 16.5.

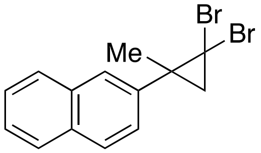

2-(2,2-Dibromo-1-methylcyclopropyl)naphthalene (S2-p): Obtained as a colorless liquid in 69% yield. Compound purified by silica gel column chromatography eluting with dichloromethane and Hexanes. R_f=0.45 (DCM: Hexanes= 15:85). ^{1}H NMR (300 MHz, $CDCl_3$) δ 7.92 – 7.80 (m, 3H), 7.70 (s, 1H), 7.56 – 7.46 (m, 3H), 2.32 (d, *J* = 7.6 Hz, 1H), 1.88 (d, *J* = 7.6 Hz, 1H), 1.80 (s, 3H). ^{13}C NMR (75 MHz, $CDCl_3$) δ 140.0, 133.3, 132.6, 128.2, 127.8, 127.8, 127.0, 126.8, 126.3, 126.1, 36.7, 36.0, 33.9, 27.7.

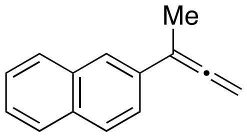

2-(Buta-2,3-dien-2-yl)naphthalene (2p):[6] Obtained as a yellow solid in 99% yield. Compound purified by silica gel column chromatography eluting with dichloromethane and Hexanes. R_f=0.5 (DCM: Hexanes= 15:85). ^{1}H NMR (300 MHz, $CDCl_3$) δ 7.88 – 7.72 (m, 4H), 7.66 (d, *J* = 8.6 Hz, 1H), 7.52 – 7.41 (m, 2H), 5.25 – 5.06 (m, 2H), 2.24 (t, *J* = 2.7 Hz, 3H). ^{13}C NMR (75 MHz, $CDCl_3$) δ 209.7, 134.1, 133.6, 132.3, 128.0, 127.7, 127.6, 126.1, 125.7, 125.0, 123.3, 100.1, 16.8.

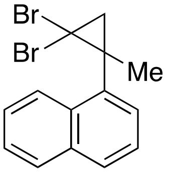

1-(2,2-Dibromo-1-methylcyclopropyl)naphthalene (S2-q): Obtained as a colorless liquid in 55% yield. Compound purified by silica gel column chromatography eluting with dichloromethane and Hexanes. R_f=0.45 (DCM: Hexanes= 15:85). ^{1}H NMR (300 MHz, $CDCl_3$) δ 8.20 (d, *J* = 8.4 Hz, 1H), 7.92 (d, *J* = 8.1 Hz, 1H), 7.81 (d, *J* = 8.2 Hz, 1H), 7.67 (t, *J* = 7.6 Hz, 1H), 7.56 (t, *J* = 8.0 Hz, 1H), 7.47 – 7.39 (m, 1H), 7.32 (d, *J* = 7.1 Hz, 1H), 2.27 (d, *J* = 7.4 Hz, 1H), 1.98 (d, *J* = 7.4 Hz, 1H), 1.85 (s, 3H). ^{13}C NMR (75 MHz, $CDCl_3$) δ 139.7, 134.0, 131.4, 128.6, 128.0, 126.2, 126.0, 125.9, 125.4, 37.3, 35.4, 34.5, 26.9.

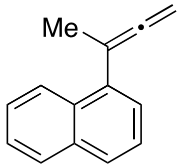

1-(Buta-2,3-dien-2-yl)naphthalene (2q):[6] Obtained as a yellow oil in 90% yield. Compound purified by silica gel column chromatography eluting with dichloromethane and Hexanes. R_f=0.5 (DCM: Hexanes= 15:85). ^{1}H NMR (300 MHz, $CDCl_3$) δ 8.24 (d, *J* = 7.4 Hz, 1H), 7.88 (d, *J* = 8.5 Hz, 1H), 7.79 (d, *J* = 7.1 Hz, 1H), 7.58 – 7.43 (m, 4H), 4.88 (q, *J* = 3.1 Hz, 2H), 2.24 (t, *J* = 3.2 Hz, 3H). ^{13}C NMR (75 MHz, $CDCl_3$) δ 208.2, 136.5, 134.0, 130.9, 128.5, 127.5, 125.9, 125.8, 125.5, 125.5, 125.0, 98.2, 74.3, 21.2.

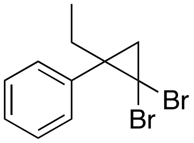

(2,2-Dibromo-1-ethylcyclopropyl)benzene (S2-r): Obtained as a colorless liquid in 80% yield. Compound purified by silica gel column chromatography eluting with Hexanes. R_f=0.5 (Hexanes). ^{1}H NMR (300 MHz, $CDCl_3$) δ 7.37 (d, *J* = 7.5 Hz, 2H), 7.31 – 7.28 (m, 3H), 2.25 – 2.13 (m, 1H), 2.07 (d, *J* = 7.4 Hz, 1H), 1.90 – 1.77 (m, 1H), 1.75 (d, *J* = 7.5 Hz, 1H), 0.89 (t, *J* = 7.4 Hz, 3H). ^{13}C NMR (75 MHz, $CDCl_3$) δ 140.2, 129.4, 128.1, 127.2, 41.0, 36.8, 33.7, 32.8, 11.3.

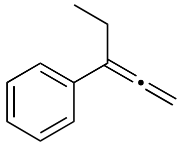

(Penta-1,2-dien-3-yl)benzene (2r):[6] Obtained as a colorless liquid in 71% yield. Compound purified by silica gel column chromatography eluting with Hexanes. R_f=0.6 (Hexanes). ^{1}H NMR (300 MHz, $CDCl_3$) δ 7.49 (d, *J* = 7.8 Hz, 2H), 7.41 – 7.36 (m, 2H), 7.26 (t, *J* = 6.6 Hz, 1H), 5.17 (s, 2H), 2.54 – 2.48 (m, 2H), 1.28 – 1.21 (m, 3H). ^{13}C NMR (75 MHz, $CDCl_3$) δ 208.4, 136.5, 128.3, 126.5, 125.9, 106.7, 78.7, 22.4, 12.4.

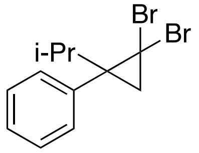

(2,2-Dibromo-1-isopropylcyclopropyl)benzene (S2-s): Obtained as a colorless liquid in 59% yield. Compound purified by silica gel column chromatography eluting with Hexanes. R_f=0.65 (Hexanes). ^{1}H NMR (300 MHz, $CDCl_3$) δ 7.52 – 7.26 (m, 4H), 7.07 (s, 1H), 2.03 – 1.85 (m, 2H), 1.79 (d, *J* = 7.1 Hz, 1H), 1.12 (d, *J* = 6.7 Hz, 3H), 0.88 (d, *J* = 6.8 Hz, 3H). ^{13}C NMR (75 MHz, $CDCl_3$) δ 136.8, 132.4, 130.7, 128.0, 127.3, 126.9, 44.1, 37.9, 36.9, 35.4, 20.2, 19.1.

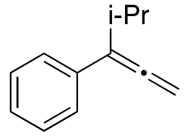

(**4-Methylpenta-1,2-dien-3-yl)benzene (2s):**[9] Obtained as a colorless liquid in 77% yield. Compound purified by silica gel column chromatography eluting with Hexanes. R_f=0.75 (Hexanes). ^{1}H NMR (300 MHz, $CDCl_3$) δ 7.39 (d, *J* = 7.8 Hz, 2H), 7.35 – 7.17 (m, 3H), 5.07 (d, *J* = 2.3 Hz, 2H), 2.85 – 2.74 (m, 1H), 1.14 (d, *J*=0.75Hz, 3H), 1.12 (d, *J*=0.75Hz, 3H). ^{13}C NMR (75 MHz, $CDCl_3$) δ 207.5, 136.4, 130.9, 128.2, 127.6, 126.4, 112.1, 78.9, 27.2, 22.0.

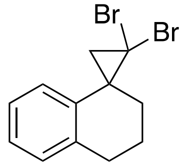

2,2-Dibromo-3',4'-dihydro-2'H-spiro[cyclopropane-1,1'-naphthalene] (S2-t): Obtained as a colorless oil in 68% yield. Compound purified by silica gel column chromatography eluting with Hexanes. R_f=0.45 (Hexanes). ^{1}H NMR (300 MHz, $CDCl_3$) δ 7.22 – 7.15 (m, 3H), 6.98 (d, *J* = 7.7 Hz, 1H), 3.05 – 2.86 (m, 2H), 2.44 (d, *J* = 8.2 Hz, 1H), 2.35 – 2.02 (m, 3H), 2.01 – 1.87 (m, 1H), 1.82 (d, *J* = 8.2 Hz, 1H). ^{13}C NMR (75 MHz, $CDCl_3$) δ 139.5, 135.6, 128.5, 127.4, 126.2, 124.9, 39.5, 34.9, 33.5, 31.2, 29.2, 20.7.

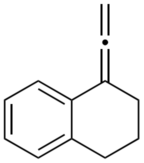

1-Vinylidene-1,2,3,4-tetrahydronaphthalene (2t):[11] Obtained as a colorless oil in 85% yield. Compound purified by silica gel column chromatography eluting with Hexanes. R_f=0.55 (Hexanes). ^{1}H NMR (300 MHz, $CDCl_3$) δ 7.58 (d, *J* = 7.4 Hz, 1H), 7.28 – 7.10 (m, 3H), 5.14 (s, 2H), 2.87 (t, *J* = 6.3 Hz, 2H), 2.73 – 2.57 (m, 2H), 1.97 (p, *J* = 6.1 Hz, 2H). ^{13}C NMR (75 MHz, $CDCl_3$) δ 206.5, 136.3, 131.1, 129.1, 126.8, 126.5, 126.0, 101.0, 77.9, 30.1, 28.7, 22.8.

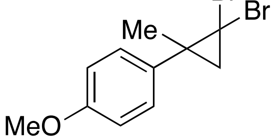

1-(2,2-Dibromo-1-methylcyclopropyl)-4-methoxybenzene (S2-u): Obtained as a colorless liquid in 46% yield. Compound purified by silica gel column chromatography eluting with dichloromethane and Hexanes. R_f=0.30 (DCM: Hexanes= 15: 85). ^{1}H NMR (300 MHz, $CDCl_3$) δ 7.20 (d, *J* = 8.7 Hz, 2H), 6.87 (d, *J* = 8.7 Hz, 2H), 3.79 (s, 3H), 2.10 (d, *J* = 7.5 Hz, 1H), 1.73 (d, *J* = 7.5 Hz, 1H), 1.68 (s, 3H). ^{13}C NMR (75 MHz, $CDCl_3$) δ 158.5, 134.5, 129.4, 113.6, 55.1, 37.3, 35.0, 33.7, 27.6.

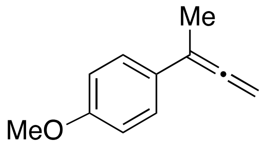

1-(Buta-2,3-dien-2-yl)-4-methoxybenzene (2u):[6] Obtained as a colorless oil in 75% yield. Compound purified by silica gel column chromatography eluting with dichloromethane and Hexanes. R_f=0.45 (DCM: Hexanes= 15:85). ^{1}H NMR (300 MHz, $CDCl_3$) δ 7.35 (d, *J* = 8.7 Hz, 2H), 6.88 (d, *J* = 8.8 Hz, 2H), 5.01 (q, *J* = 3.1 Hz, 2H), 3.81 (s, 3H), 2.09 (t, *J* = 3.1 Hz, 3H). ^{13}C NMR (75 MHz, $CDCl_3$) δ 208.5, 158.4, 128.9, 126.7, 113.7, 99.2, 76.7, 55.2, 16.8.

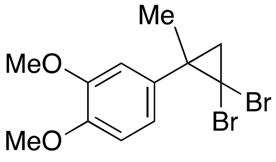

4-(2,2-Dibromo-1-methylcyclopropyl)-1,2-dimethoxybenzene (S2-v): Obtained as a colorless liquid in 73% yield. Compound purified by silica gel column chromatography eluting with EtOAc and Hexanes. R_f=0.3 (EtOAc : Hexanes= 1:10). ^{1}H NMR (300 MHz, $CDCl_3$) δ 6.79 (s, 3H), 3.89 (s, 3H), 3.85 (s, 3H), 2.10 (d, *J* = 7.5 Hz, 1H), 1.73 (d, *J* = 7.5 Hz, 1H), 1.68 (s, 3H). ^{13}C NMR (75 MHz, $CDCl_3$) δ 148.6, 148.1, 134.9, 120.3, 111.7, 110.7, 55.9, 55.7, 37.1, 35.4, 33.9, 27.6.

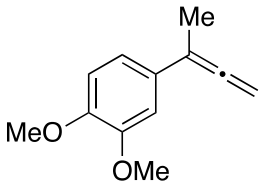

4-(Buta-2,3-dien-2-yl)-1,2-dimethoxybenzene (2v):[1] Obtained as a colorless oil in 89% yield. Compound purified by silica gel column chromatography eluting with EtOAc and Hexanes. R_f=0.45 (EtOAc: Hexanes= 1:10). ^{1}H NMR (300 MHz, $CDCl_3$) δ 6.96 (d, *J* = 1.9 Hz, 1H), 6.89 (dd, *J* = 8.4, 2.0 Hz, 1H), 6.81 (d, *J* = 8.4 Hz, 1H), 5.00 (q, *J* = 3.1 Hz, 2H), 3.86 (s, 3H), 3.85 (s, 3H), 2.06 (t, *J* = 3.1 Hz, 3H). ^{13}C NMR (75 MHz, $CDCl_3$) δ 208.5, 148.7, 148.0, 129.2, 117.5, 110.9, 109.2, 99.5, 76.8, 55.8, 55.7, 16.7.

General procedure for chlorination of allenes:

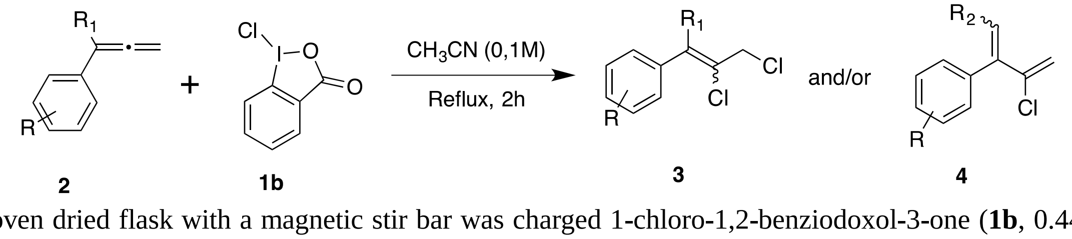

To an oven dried flask with a magnetic stir bar was charged 1-chloro-1,2-benziodoxol-3-one (**1b**, 0.44 mmol, 2.2 equiv) and acetonitrile (1 mL), and the resulting solution was placed under a nitrogen atmosphere. The reaction flask was immersed in a preheated 85 °C oil bath and stirred. To this, a solution of allenes **2a–v** (0.2 mmol, 1.0 equiv) in acetonitrile (1 mL) was added by syringe pump over 60 min. Then the reaction was monitored by TLC analysis, and upon consumption of the allene (≈1 h), the reaction mixture was cooled to room temperature and concentrated under vacuum by rotary evaporation. The resulting crude product mixture was purified by silica gel chromatography to provide the chlorination products **3a–4v**.

Characterization data for chlorination:

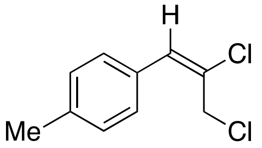

(*E*)-1-(2,3-Dichloroprop-1-en-1-yl)-4-methylbenzene (*E*-3a):[12] Obtained as a white solid in 40% yield. Compound purified by silica gel column chromatography eluting with Hexanes. R_f=0.6 (Hexanes). IR (ATR) 3025, 1738, 1637, 1511, 1366, 1261, 1222, 1187, 1092, 808, 700, 626cm^{-1}. ^{1}H NMR (500 MHz, $CDCl_3$) δ 7.20 (s, 4H), 6.88 (s, 1H), 4.36 (s, 2H), 2.36 (s, 3H). ^{13}C NMR (126 MHz, $CDCl_3$) δ 139.0, 133.7, 132.2, 131.5, 130.2, 128.8, 46.1, 21.9. HRMS-APCI calcd for $C_{10}H_{10}Cl_2$ $[M]^+$ 200.0154, found 200.0155.

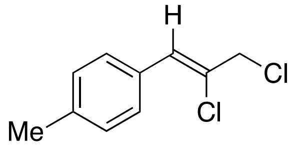

(*Z*)-1-(2,3-Dichloroprop-1-en-1-yl)-4-methylbenzene (*Z*-3a*)*:[12] Obtained as a white solid in 50% yield. Compound purified by silica gel column chromatography eluting with Hexanes. R_f=0.50 (Hexanes). IR (ATR) 2922, 1738, 1609, 1494, 1261, 1216, 1092, 873, 808, 700, 604cm^{-1}. ^{1}H NMR (500 MHz, $CDCl_3$) δ 7.56 (d, *J* = 8.1 Hz, 2H), 7.19 (d, *J* = 7.9 Hz, 2H), 6.80 (s, 1H), 4.35 (s, 2H). ^{13}C NMR (126 MHz, $CDCl_3$) δ 139.5, 131.5, 129.9, 129.7, 129.3, 128.6, 51.4, 22.0. HRMS-APCI calcd for $C_{10}H_{10}Cl_2$ $[M]^+$ 200.0154, found 200.0155.

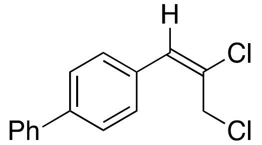

(*E*)-4-(2,3-Dichloroprop-1-en-1-yl)-1,1'-biphenyl (*E*-3b): Obtained as a white solid in 36% yield. Compound purified by silica gel column chromatography eluting with Hexanes. R_f=0.40 (Hexanes). IR (ATR) 2970, 1738, 1606,1435, 1389, 1259, 1073, 1043, 902, 689, 518cm^{-1}. ^{1}H NMR (500 MHz, $CDCl_3$) δ 7.63 (d, *J* = 8.3 Hz, 2H), 7.60 (d, *J* = 7.1 Hz, 2H), 7.46 (t, *J* = 7.6 Hz, 2H), 7.38 (m, 3H), 6.95 (s, 1H), 4.41 (s, 2H). ^{13}C NMR (126 MHz, $CDCl_3$) δ 141.8, 140.8, 134.0, 133.3, 132.2, 129.6, 129.4, 128.4, 128.2, 127.7, 46.1. HRMS-APCI calcd for $C_{15}H_{12}Cl_2$ $[M]^+$ 262.0311, found 262.0312.

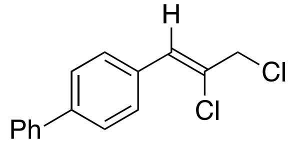

(*Z*)-4-(2,3-Dichloroprop-1-en-1-yl)-1,1'-biphenyl (*Z*-*3b*): Obtained as a white solid in 52% yield. Compound purified by silica gel column chromatography eluting with Hexanes. Rf=0.3 (Hexanes). IR (ATR) 2970, 1738, 1626, 1588, 1487, 1566, 1216, 1108, 1010, 930, 704, 531cm-1. 1H NMR (500 MHz, $CDCl_3$) δ 7.75 (d, J = 8.1 Hz, 2H), 7.62 (d, J = 8.3 Hz, 4H), 7.45 (t, J = 7.6 Hz, 2H), 7.37 (t, J = 7.4 Hz, 1H), 6.88 (s, 1H), 4.38 (s, 2H). 13C NMR (126 MHz, $CDCl_3$) δ 142.1, 141.0, 133.3, 130.4, 129.5, 129.0, 128.3, 127.7, 127.6, 51.3. HRMS-APCI calcd for $C_{15}H_{12}Cl_2$ $[M]^+$ 262.0311, found 262.0312.

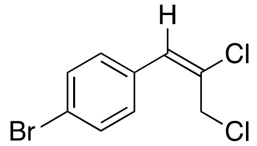

(*E*)-1-Bromo-4-(2,3-dichloroprop-1-en-1-yl)benzene (*E*-3c): Obtained as a colorless liquid in 34% yield. Compound purified by silica gel column chromatography eluting with Hexanes. R_f=0.40 (Hexanes). IR (ATR) 3029, 2970, 1738, 1486, 1365, 1045, 766, 721, 697 cm^{-1}. ^{1}H NMR (500 MHz, $CDCl_3$) δ 7.51 (s, 4H), 6.78 (s, 1H), 4.33 (s, 2H). ^{13}C NMR (126 MHz, $CDCl_3$) δ 133.2, 132.2, 131.4, 130.4, 128.1, 123.4, 50.8. HRMS-APCI calcd for $C_9H_7BrCl_2$ $[M]^+$ 263.9103, found 263.9104.

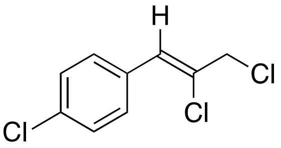

(*Z*)-1-Chloro-4-(2,3-dichloroprop-1-en-1-yl)benzene (*Z*-3d):[12] Obtained as a colorless liquid in 18% yield. Compound purified by silica gel column chromatography eluting with Hexanes. R_f=0.45 (Hexanes). IR (ATR) 2970, 1738, 1365, 1228, 1216, 1092, 588, 527cm^{-1}. ^{1}H NMR (500 MHz, $CDCl_3$) δ 7.58 (d, *J* = 8.5 Hz, 2H), 7.35 (d, *J* = 8.6 Hz, 2H), 6.80 (s, 1H), 4.34 (s, 2H). ^{13}C NMR (126 MHz, $CDCl_3$) δ 135.1, 132.7, 131.2, 130.2, 129.2, 128.0, 50.9. HRMS-APCI calcd for $C_9H_7Cl_3$ $[M]^+$ 219.9608, found 219.9609.

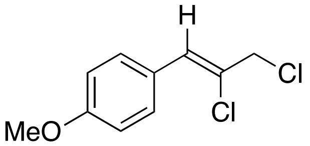

(*Z*)-1-(2,3-Dichloroprop-1-en-1-yl)-4-methoxybenzene (*Z*-3g):[12] Obtained as a colorless liquid in 52% yield. Compound purified by silica gel column chromatography eluting with Hexanes. R_f=0.30 (DCM: Hexanes= 15: 85). IR (ATR) 2957, 1738, 1605, 1510, 1441, 1297, 1251, 1178, 1032, 825, 699, 537cm^{-1}. ^{1}H NMR (500 MHz, $CDCl_3$) δ 7.64 (d, *J* = 8.8 Hz, 2H), 6.90 (d, *J* = 8.8 Hz, 2H), 6.76 (s, 1H), 4.35 (s, 2H), 3.83 (s, 3H). ^{13}C NMR (126 MHz, $CDCl_3$) δ 160.5, 133.4, 131.5, 130.3, 129.0, 114.4, 56.0, 51.7. HRMS-APCI calcd for $C_{10}H_{10}OCl_2$ $[M]^+$ 216.0103, found216.0105.

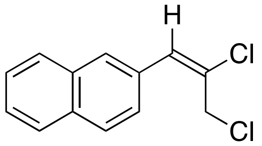

(*E*)-2-(2,3-Dichloroprop-1-en-1-yl)naphthalene (*E*-3h): Obtained as a white solid in 26% yield. Compound purified by silica gel column chromatography eluting with Hexanes. R_f=0.38 (Hexanes). IR (ATR) 2991, 1738, 1598, 1431, 1259, 1216, 1047, 904, 817, 724 cm^{-1}. ^{1}H NMR (500 MHz, $CDCl_3$) δ 7.90 – 7.83 (m, 3H), 7.81 (s, 1H), 7.53 – 7.49 (m, 2H), 7.40 (d, *J* = 8.5 Hz, 1H), 7.07 (s, 1H), 4.42 (s, 2H). ^{13}C NMR (126 MHz, $CDCl_3$) δ 133.9, 133.8, 133.5, 132.5, 132.5, 129.2, 128.9, 128.4, 128.3, 127.4, 127.3, 126.4, 46.2. HRMS-APCI calcd for $C_{13}H_{10}Cl_2$ $[M]^+$ 236.0154, found 236.0154.

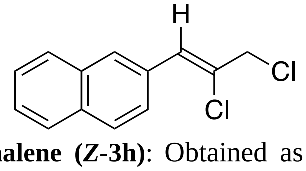

(*Z*)-2-(2,3-Dichloroprop-1-en-1-yl)naphthalene (*Z*-3h): Obtained as a white solid in 67% yield. Compound purified by silica gel column chromatography eluting with Hexanes. R_f=0.30 (Hexanes). IR (ATR) 2927, 1738, 1505, 1365, 1229, 1184, 1018, 868, 744, 700 cm^{-1}. ^{1}H NMR (500 MHz, $CDCl_3$) δ 8.13 (s, 1H), 7.84 (m, 3H), 7.76 (d, *J* = 10.2 Hz, 1H), 7.50 (m, 2H), 7.00 (s, 1H), 4.41 (s, 2H). ^{13}C NMR (126 MHz, $CDCl_3$) δ 133.8, 133.7, 131.8, 129.8, 129.8, 129.4, 129.0, 128.5, 128.3, 127.4, 127.2, 127.0, 51.3. HRMS-APCI calcd for $C_{13}H_{10}Cl_2$ $[M]^+$ 236.0154, found 236.0154.

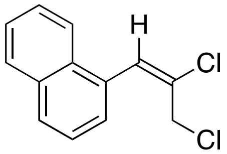

(*E*)-1-(2,3-Dichloroprop-1-en-1-yl)naphthalene (*E*-3i): Obtained as a colorless liquid in 28% yield. Compound purified by silica gel column chromatography eluting with Hexanes. R_f=0.45 (Hexanes). IR (ATR) 2984, 1738, 1428, 1258, 1083, 1036, 931, 803, 723 cm^{-1}. ^{1}H NMR (500 MHz, $CDCl_3$) δ 7.93 – 7.83 (m, 3H), 7.59 – 7.51 (m, 2H), 7.51 – 7.48 (m, 2H), 7.37 (s, 1H), 4.28 (s, 2H). ^{13}C NMR (126 MHz, $CDCl_3$) δ 134.2, 133.5, 132.2, 132.0, 131.9, 129.8, 129.2, 127.4, 127.1, 127.0, 126.1, 125.0, 45.8. HRMS-APCI calcd for $C_{13}H_{10}Cl_2$ $[M]^+$ 236.0154, found 236.0154.

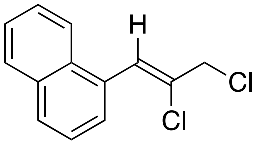

(*Z*)-1-(2,3-Dichloroprop-1-en-1-yl)naphthalene (*Z*-3i): Obtained as a colorless liquid in 50% yield. Compound purified by silica gel column chromatography eluting with Hexanes. R_f=0.35 (Hexanes). IR (ATR) 2926, 1738, 1508, 1366, 1183, 1045, 1014, 860, 696 cm^{-1}. ^{1}H NMR (500 MHz, $CDCl_3$) δ 7.89 (m, 2H), 7.85 (d, *J* = 8.2 Hz, 1H), 7.70 (d, *J* = 7.1 Hz, 1H), 7.57 – 7.47 (m, 3H), 7.40 (s, 1H), 4.48 (s, 2H). ^{13}C NMR (126 MHz, $CDCl_3$) δ 134.1, 132.5, 131.9, 131.6, 129.5, 129.3, 127.9, 127.6, 127.1, 126.7, 125.8, 124.7, 50.0. HRMS-APCI calcd for $C_{13}H_{10}Cl_2$ $[M]^+$ 236.01541, found 236.01544.

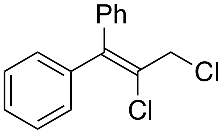

(2,3-Dichloroprop-1-ene-1,1-diyl)dibenzene (3j): Obtained as a colorless quid in 84% yield. Compound purified by silica gel column chromatography eluting with DCM and Hexanes. R_f=0.3 (DCM: Hexanes= 15:85). IR (ATR) 3057, 1738, 1492, 1443, 1262, 1165, 1083, 1001, 899, 764, 696 cm^{-1}. ^{1}H NMR (500 MHz, $CDCl_3$) δ 7.40 – 7.32 (m, 5H), 7.32 – 7.27 (m, 5H), 4.34 (s, 2H). ^{13}C NMR (126 MHz, $CDCl_3$) δ 144.6, 140.5, 140.1, 130.0, 129.6, 129.3, 129.0, 128.7, 128.7, 128.6, 48.6. HRMS-APCI calcd for $C_{15}H_{12}Cl_2$ $[M]^+$ 262.0311, found 262.0313.

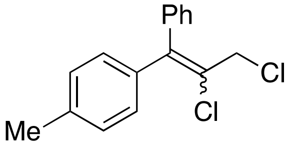

1-(2,3-Dichloro-1-phenylprop-1-en-1-yl)-4-methylbenzene (3k): Obtained as a white solid in 79% yield. Compound purified by silica gel column chromatography eluting with Hexanes. R_f=0.3 (Hexanes). IR (ATR) 2922, 1738, 1609, 1494, 1261, 1216, 1092, 873, 808, 700, 604 cm^{-1}. (***Z*-3j**) ^{1}H NMR (500 MHz, $CDCl_3$) δ 7.40 – 7.31 (m, 2H), 7.31 – 7.24 (m, 3H), 7.21 – 7.14 (m, 4H), 4.33 (s, 2H), 2.35 (s, 3H). ^{13}C NMR (126 MHz, $CDCl_3$) δ 144.6, 140.7, 138.9, 137.6, 130.0, 129.9, 129.6, 129.2, 128.7, 128.2, 48.8, 22.0. ***(E*-3j)** ^{1}H NMR (500 MHz, $CDCl_3$) δ 7.40 – 7.31 (m, 2H), 7.31 – 7.24 (m, 3H), 7.21 – 7.14 (m, 4H), 4.36 (s, 2H), 2.36 (s, 3H). ^{13}C NMR (126 MHz, $CDCl_3$) δ 144.6, 140.4, 138.9, 138.6, 137.3, 130.0, 129.7, 129.4, 128.9, 128.6, 128.1, 48.8, 21.9. HRMS-APCI calcd for $C_{16}H_{14}Cl_2$ $[M]^+$ 276.0467, found 276.0469.

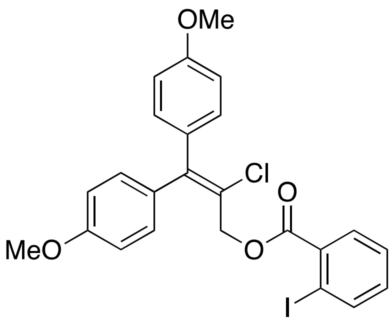

2-Chloro-3,3-bis(4-methoxyphenyl)allyl 2-iodobenzoate (3m"): Obtained as a white solid in 57% yield. Compound purified by silica gel column chromatography eluting with Hexanes. R_f=0.3 (Hexanes). IR (ATR) 2922, 1738, 1609, 1494, 1261, 1216, 1092, 873, 808, 700, 604 cm^{-1}. ^{1}H NMR (500 MHz, $CDCl_3$) δ 8.01 (d, *J* = 7.9 Hz, 1H), 7.92 (d, *J* = 7.8 Hz, 1H), 7.43 (t, *J* = 8.1 Hz, 1H), 7.26 (d, *J* = 8.8 Hz, 2H), 7.17 (t, *J* = 7.7 Hz, 1H), 7.13 (d, *J* = 8.7 Hz, 2H), 6.89 – 6.82 (m, 4H), 5.08 (s, 2H), 3.81 (s, 3H), 3.80 (s, 3H). ^{13}C NMR (126 MHz, $CDCl_3$) δ 166.6, 160.2, 159.8, 145.0, 142.1, 135.4, 133.5, 133.2, 132.8, 131.9, 131.8, 131.4, 129.5, 128.6, 124.9, 114.5, 114.0, 94.9, 68.0, 56.0, 55.9. HRMS-ESI calcd for $C_{24}H_{20}ClIO_4$ $[M]^+$ 534.0089, found 534.0089.

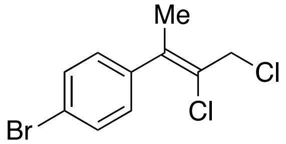

(*Z*)-1-Bromo-4-(3,4-dichlorobut-2-en-2-yl)benzene (*Z*-3n): Obtained as a colorless liquid in 23% yield. Compound purified by silica gel column chromatography eluting with Hexanes. R_f=0.3 (Hexanes). IR (ATR) 2924, 1738, 1487, 1075, 1009, 769, 621, 514 cm^{-1}. ^{1}H NMR (500 MHz, $CDCl_3$) δ 7.52 (d, *J* = 8.3 Hz, 2H), 7.14 (d, *J* = 8.3 Hz, 2H), 4.12 (s, 2H), 2.19 (s, 3H). ^{13}C NMR (126 MHz, $CDCl_3$) δ 139.7, 139.4, 132.5, 129.8, 128.4, 122.8, 47.7, 23.4. HRMS-APCI calcd for $C_{10}H_9BrCl_2$ $[M]^+$ 277.9259, found 277.9260.

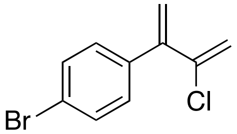

1-Bromo-4-(3-chlorobuta-1,3-dien-2-yl)benzene (4n): Obtained as a colorless liquid in 31% yield. Compound purified by silica gel column chromatography eluting with Hexanes. R_f=0.7 (Hexanes). IR (ATR) 2926, 1738, 1587, 1487, 1228, 1073, 831, 766, 527 cm^{-1}. ^{1}H NMR (500 MHz, $CDCl_3$) δ 7.48 (d, *J* = 8.5 Hz, 2H), 7.20 (d, *J* = 8.5 Hz, 2H), 5.78 (s, 1H), 5.54 (s, 1H), 5.35 (s, 1H), 5.28 (s, 1H). ^{13}C NMR (126 MHz, $CDCl_3$) δ 146.0, 140.5, 138.6, 132.1, 130.8, 122.8, 119.8, 118.0. HRMS-APCI calcd for $C_{10}H_8BrCl$ $[M+H]^+$ 242.9580, found 242.9575.

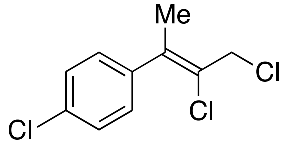

(*Z*)-1-Chloro-4-(3,4-dichlorobut-2-en-2-yl)benzene (*Z*-3o): Obtained as a colorless liquid in 34% yield. Compound purified by silica gel column chromatography eluting with Hexanes. R_f=0.35 (Hexanes). IR (ATR) 2925, 1738, 1593, 1489, 1373, 1217, 1091, 1014, 831, 712 cm^{-1}. ^{1}H NMR (500 MHz, $CDCl_3$) δ 7.36 (d, *J* = 8.4 Hz, 2H), 7.20 (d, *J* = 8.4 Hz, 2H), 4.12 (s, 2H), 2.19 (s, 3H). ^{13}C NMR (126 MHz, $CDCl_3$) δ 139.4, 139.2, 134.7, 129.6, 129.5, 128.4, 47.7, 23.5. HRMS-APCI calcd for $C_{10}H_9Cl_3$ $[M]^+$ 233.9764, found 233.9765.

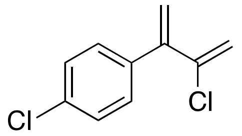

1-chloro-4-(3-chlorobuta-1,3-dien-2-yl)benzene (4o): Obtained as a colorless liquid in 38% yield. Compound purified by silica gel column chromatography eluting with Hexanes. R_f=0.45 (Hexanes). IR (ATR) 2927, 1738, 1593, 1489, 1365, 1228, 1091, 1014, 906, 832, 527 cm^{-1}. ^{1}H NMR (500 MHz, $CDCl_3$) δ 7.32 (d, *J* = 8.5 Hz, 2H), 7.26 (d, *J* = 8.5 Hz, 2H), 5.78 (s, 1H), 5.54 (s, 1H), 5.34 (s, 1H), 5.28 (s, 1H). ^{13}C NMR (126 MHz, $CDCl_3$) δ 146.0, 140.6, 138.1, 134.6, 130.5, 129.1, 119.8, 117.9. HRMS-APCI calcd for $C_{10}H_8Cl_2$ $[M]^+$ 197.9998, found 197.9998.

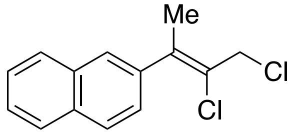

(*Z*)-2-(3,4-Dichlorobut-2-en-2-yl)naphthalene (*Z*-3p): Obtained as a colorless liquid in 37% yield. Compound purified by silica gel column chromatography eluting with dichloromethane and Hexanes. R_f=0.35 (DCM: Hexanes= 15:85). IR (ATR) 2980, 1738, 1429, 1372, 1222, 819, 748, 699 cm^{-1}. ^{1}H NMR (500 MHz, $CDCl_3$) δ 7.87 – 7.84 (m, 3H), 7.75 (s, 1H), 7.58 – 7.48 (m, 2H), 7.36 (d, *J* = 9.8 Hz, 1H), 4.21 (s, 2H), 2.30 (s, 3H). ^{13}C NMR (126 MHz, $CDCl_3$) δ 140.6, 138.2, 133.7, 133.4, 129.1, 128.8, 128.4, 128.2, 127.3, 127.2, 127.1, 126.0, 48.0, 23.6. HRMS-APCI calcd for $C_{10}H_{10}Cl_2$ $[M]^+$ 200.0154, found 200.0155.

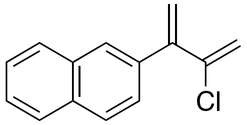

2-(3-Chlorobuta-1,3-dien-2-yl)naphthalene (4p): Obtained as a colorless liquid in 58% yield. Compound purified by silica gel column chromatography eluting with dichloromethane and Hexanes. R_f=0.65 (DCM:Hexanes = 15:85). IR (ATR) 2970, 1738, 1434, 1229, 1216, 1107, 860, 750, 478 cm^{-1}. ^{1}H NMR (500 MHz, $CDCl_3$) δ 7.88 – 7.79 (m, 4H), 7.54 – 7.47 (m, 2H), 7.45 (d, *J* = 10.0 Hz, 1H), 5.87 (s, 1H), 5.58 (s, 1H), 5.48 (s, 1H), 5.34 (s, 1H). ^{13}C NMR (126 MHz, $CDCl_3$) δ 147.1, 140.9, 137.2, 133.8, 133.6, 128.7, 128.4, 128.3, 128.2, 127.2, 127.0, 126.9, 119.8, 118.0. HRMS-APCI calcd for $C_{14}H_{11}Cl$ $[M]^+$ 214.0544, found 214.0545.

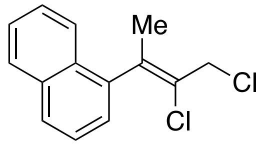

(*Z*)-1-(3,4-Dichlorobut-2-en-2-yl)naphthalene (*Z*-3q): Obtained as a colorless liquid in 20% yield. Compound purified by silica gel column chromatography eluting with dichloromethane and Hexanes. R_f=0.35 (DCM: Hexanes= 15:85). IR (ATR) 2922, 1738, 1506, 1427, 1371, 1216, 1198, 1019, 801, 777, 701 cm^{-1}. ^{1}H NMR (500 MHz, $CDCl_3$) δ 7.88 (dd, *J* = 6.5, 3.1 Hz, 1H), 7.83 (d, *J* = 8.3 Hz, 1H), 7.76 – 7.70 (m, 1H), 7.55 – 7.50 (m, 2H), 7.48 (t, *J* = 7.6 Hz, 1H), 7.31 (d, *J* = 7.0 Hz, 1H), 3.98 (s, 2H), 2.28 (s, 3H). ^{13}C NMR (126 MHz, $CDCl_3$) δ 139.2, 138.3, 134.5, 131.1, 129.2, 129.0, 129.0, 127.3, 126.9, 126.2, 125.8, 125.6, 47.5, 23.6. HRMS-APCI calcd for $C_{14}H_{12}Cl_2$ $[M]^+$ 250.0311, found 250.0313.

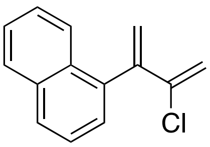

1-(3-Chlorobuta-1,3-dien-2-yl)naphthalene (4q): Obtained as a colorless liquid in 65% yield. Compound purified by silica gel column chromatography eluting with dichloromethane and Hexanes. R_f=0.65 (DCM:Hexanes= 15:85). IR (ATR) 2926, 1738, 1506, 1400, 1370, 1216, 1152, 1089, 888, 802, 778 cm^{-1}. ^{1}H

NMR (500 MHz, $CDCl_3$) δ 7.85 (m, 3H), 7.47 (m, 3H), 7.37 (d, *J* = 7.0 Hz, 1H), 6.18 (s, 1H), 5.44 (s, 2H), 4.90 (s, 1H). ^{13}C NMR (126 MHz, $CDCl_3$) δ 144.9, 140.8, 137.9, 134.1, 132.4, 128.9, 128.8, 127.6, 126.8, 126.6, 126.5, 125.9, 121.7, 118.3. HRMS-APCI calcd for $C_{14}H_{11}Cl$ $[M]^+$ 214.0544, found 214.0541.

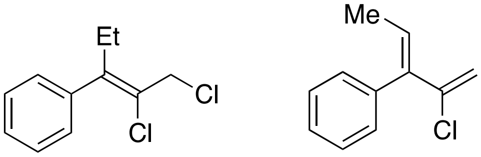

(*Z*)-(1,2-Dichloropent-2-en-3-yl)benzene (*Z*-3r) and **(*E*)-(2-chloropenta-1,3-dien-3-yl)benzene (4r):** Obtained as a colorless liquid in 40% yield of ***Z*-3r** and 25% yield of **4r**. Compound purified by silica gel column chromatography eluting with Hexanes. R_f=0.45 (Hexanes). ^1H NMR (500 MHz, $CDCl_3$) δ 7.41 (d, J = 7.3 Hz, 3H), 7.38 (d, J = 7.5 Hz, 3H), 7.33 (t, J = 7.4 Hz, 3H), 7.28 (d, J = 7.1 Hz, 1H), 7.23 – 7.20 (m, 3H), 6.10 (q, J = 7.0 Hz, 1H, **4r**), 5.72 (s, 1H, **4r**), 5.38 (s, 1H, **4r**), 4.09 (s, 3H, ***Z*-3r**), 2.61 (q, J = 7.5 Hz, 3H, ***Z*-3r**), 1.95 (d, J = 7.0 Hz, 3H, **4r**), 0.96 (t, J = 7.5 Hz, 5H, ***Z*-3r**). ^{13}C NMR (126 MHz, $CDCl_3$) δ (***Z*-3r**):140.9, 139.5, 129.2, 128.6, 127.7, 127.1, 48.1, 30.0, 11.9; (**4r**): 146.2, 139.1, 137.3, 129.0, 128.6, 128.2, 127.2, 118.6, 16.3.

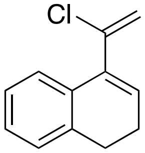

4-(1-Chlorovinyl)-1,2-dihydronaphthalene (4t): Obtained as a colorless liquid in 74% yield. Compound purified by silica gel column chromatography eluting with Hexanes. R_f=0.45 (Hexanes). IR (ATR) 2936, 1737, 1489, 1366, 1233, 1217, 894, 759, 736, 589 cm^{-1}. ^1H NMR (500 MHz, $CDCl_3$) δ 7.33 (d, *J* = 7.2 Hz, 1H), 7.24 – 7.19 (m, 1H), 7.17 (td, *J* = 6.9, 3.3 Hz, 2H), 6.30 (t, *J* = 4.7 Hz, 1H), 5.53 (s, 1H), 5.50 (s, 1H), 2.78 (t, *J* = 8.0 Hz, 2H), 2.34 (m, 2H). ^{13}C NMR (126 MHz, $CDCl_3$) δ 140.1, 138.4, 137.0, 132.6, 130.6, 128.3, 128.2, 127.1, 125.9, 116.4, 28.3, 23.8. HRMS-APCI calcd for $C_{12}H_{11}Cl$ $[M]^+$ 190.0544, found190.0541.

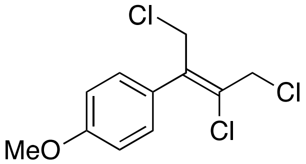

(*E*)-1-Methoxy-4-(1,3,4-trichlorobut-2-en-2-yl)benzene (5u): Obtained as a colorless liquid in 53% yield. Compound purified by silica gel column chromatography eluting with dichloromethane and Hexanes. R_f=0.30 (DCM: Hexanes= 15:85). IR (ATR) 2931, 1738, 1606, 1510, 1288, 1248, 1177, 1094, 835, 705, 603 cm^{-1}.^1H NMR (500 MHz, , $CDCl_3$) δ 7.26 (d, *J* = 8.8 Hz, 2H), 6.94 (d, *J* = 8.8 Hz, 2H), 4.47 (s, 2H), 4.14 (s, 2H), 3.83 (s, 3H). ^{13}C NMR (126 MHz, $CDCl_3$) δ 160.6, 139.2, 132.6, 130.1, 129.3, 114.9, 56.0, 47.3, 46.3. HRMS-APCI calcd for $C_{11}H_{11}OCl_3$ $[M]^+$ 263.9870, found 263.9870.

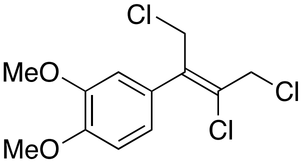

(*E*)-1,2-Dimethoxy-4-(1,3,4-trichlorobut-2-en-2-yl)benzene (5v): Obtained as a white solid in 30% yield. Compound purified by silica gel column chromatography eluting with EtOAc and Hexanes. R_f=0.45 (EtOAc: Hexanes= 1:10). IR (ATR) 2879, 2183, 1589, 1481, 1292, 1111.7, 1050, 949, 596 cm^{-1}. ^1H NMR (500 MHz, $CDCl_3$) δ 6.93 (s, 2H), 6.92 (s, 1H), 4.51 (s, 2H), 4.20 (s, 2H), 3.94 (s, 3H), 3.93 (s, 4H). ^{13}C NMR (126 MHz, $CDCl_3$) δ 149.4, 149.0, 138.7, 132.1, 129.0, 120.5, 111.3, 111.2, 56.0, 55.9, 46.9, 45.5. HRMS-APCI calcd for $C_{12}H_{13}O_2Cl_3$ $[M-Cl]^+$ 224.0599, found 224.0601.

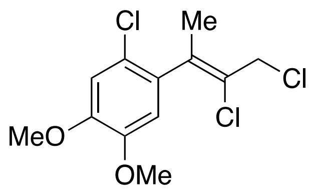

(*Z*)-1-Chloro-2-(3,4-dichlorobut-2-en-2-yl)-4,5-dimethoxybenzene (6v): Obtained as a white solid in 37% yield. Compound purified by silica gel column chromatography eluting with EtOAc and Hexanes. R_f=0.35 (EtOAc: Hexanes= 1:10). IR (ATR) 2997, 2833, 1738, 1605, 1508, 1489, 1333, 1148, 841, 571 cm^{-1}.^{1}H NMR (500 MHz, $CDCl_3$) δ 6.96 (s, 1H), 6.79 (s, 1H), 3.92 (s, 3H), 3.89 (s, 3H), 3.43 (s, 2H), 2.09 (s, 3H). ^{13}C NMR (126 MHz, $CDCl_3$) δ 149.1, 147.9, 138.3, 135.0, 133.1, 129.9, 108.5, 103.4, 57.0, 56.9, 42.9, 11.1. HRMS-APCI calcd for $C_{12}H_{13}O_2Cl_3$ $[M]^+$ 293.9981, found 293.9981.

Synthesis of 4-(propa-1,2-dien-1-yl-3,3-d$_2$)-1,1'-biphenyl) ([D$_2$]-2b)[1]:

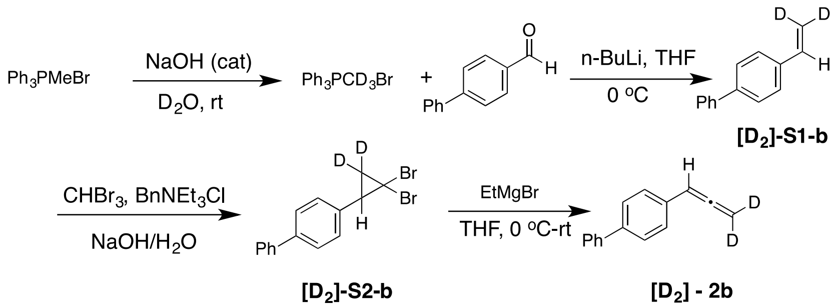

Ph_3PCD_3Br:[10] To a flask with a magnetic stir bar was added Ph_3PMeBr (530mg, 1.5 mmol, 1.0 equiv), D_2O (2 mL), and NaOH (30 mg, 0.75 mmol, 0.5 equiv). The reaction mixture was stirred under nitrogen atmosphere, and after 24 h, CH_2Cl_2 (5 mL) was added to the solution and organic layer was collected and dried with Mg_2SO_4. Then added hexanes (10 mL) to the resulting solution and storing at −5 °C for 24 h afforded colorless crystals (460 mg, 86% yield). ^{1}H NMR (300 MHz, $CDCl_3$) δ 7.71 (m, 15H). ^{2}H NMR (46 MHz, $CHCl_3$) δ 3.41 (s, 3H).

[D$_2$]–S1-b.[10] In an oven dried flask, methyl triphenylphosphonium bromide (1.0 mmol, 356 mg, 1.0 equiv) was added with THF (2.5 mL/mmol). Then 2.6M *n*-BuLi (1.2 mmol, 0.4 mL, 1.2 equiv) was added and the resulting yellow suspension was stirred in an ice-bath for 60 minutes. To this suspension, a solution of [1,1'-biphenyl]-4-carbaldehyde (1.0 mmol, 182 mg, 1.0 equiv.) was added in one portion and the resulting mixture was warmed to room temperature and further stirred at room temperature overnight. Water and DCM were added to the reaction mixture, separated and the aqueous phase was extracted with DCM (3 × 15 mL). The combined organic phases were washed with saturated NaCl solution, dried over Na_2SO_4 and the solvent removed under reduced pressure. The reaction mixture was purified by column chromatography over silica gel (200–300 mesh) using Hexanes as eluent afforded colorless solid **[D$_2$]-S1-b** (135 mg) in 74% yield. ^{1}H NMR (500 MHz, $CDCl_3$) δ 7.60 (d, *J* = 7.6 Hz, 2H), 7.57 (d, *J* = 7.9 Hz, 2H), 7.49 (d, *J* = 8.0 Hz, 2H), 7.44 (t, *J* = 7.6 Hz, 2H), 7.35 (t, *J* = 7.5 Hz, 1H), 6.75 (s, 1H). ^{13}C NMR (126 MHz, $CDCl_3$) δ 141.4, 141.2, 137.2, 137.0, 136.9, 129.4, 128.0, 127.9, 127.6, 127.3. ^{2}H NMR (46 MHz, $CHCl_3$) δ 5.95 (d, *J* = 2.7 Hz, 1H), 5.42 (d, *J* = 2.7 Hz, 1H).

[D$_2$]–S2-b and **[D$_2$]-2b** were prepared following the general procedure for allene synthesis given above[2].

4-(2,2-Dibromocyclopropyl-3,3-*d*$_2$)-1,1'-biphenyl ([D$_2$]-S2-b): Obtained as white solid in 99% yield; ^{1}H NMR (300 MHz, $CDCl_3$) δ 7.61 – 7.57 (m, 4H), 7.43 (t, *J* = 7.5 Hz, 2H), 7.36 – 7.30 (m, 3H), 6.81 (s, 1H). ^{13}C NMR (75 MHz, $CDCl_3$) δ 140.4, 140.3, 134.9, 129.2, 128.7, 127.3, 126.9, 126.9, 35.4, 28.2, 9.6. ^{2}H NMR (46

MHz, $CHCl_3$) δ 2.21 (d, *J* = 5.6 Hz, 2H).

4-(Propa-1,2-dien-1-yl-3,3-*d*$_2$)-1,1'-biphenyl) ([D$_2$]-2b): Obtained as white solid in 72% yield ^{1}H NMR (300 MHz, $CDCl_3$) δ 7.60 (d, *J* = 7.7 Hz, 2H), 7.55 (d, *J* = 7.9 Hz, 2H), 7.39 – 7.31 (m, 3H), 6.22 (s, 1H). ^{13}C NMR (75 MHz, $CDCl_3$) δ 210.0, 140.7, 139.6, 132.9, 128.7, 127.2, 127.1, 127.0, 126.8, 93.7, 78.8. ^{2}H NMR (46 MHz, $CHCl_3$) δ 5.32 (s, 2H).

Synthesis of 4-(2,3-dichloroprop-1-en-1-yl-3,3-d$_2$)-1,1'-biphenyl ([D$_2$]-3b):

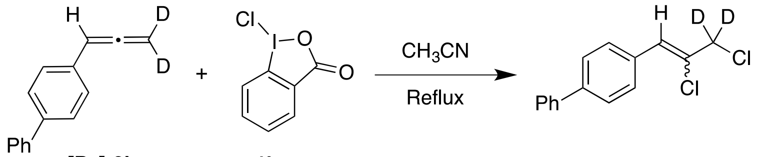

[D$_2$]-2b **1b** **[D$_2$] - 3b** 79% *E/Z*=1/2

To an oven dried flask with a magnetic stir bar was charged 4-(propa-1,2-dien-1-yl-3,3-*d*$_2$)-1,1'-biphenyl) (**[D$_2$]-2b**, 0.2 mmol, 39 mg, 1.0 equiv), 1-chloro-1,2-benziodoxol-3-one (**1b**, 0.44 mmol, 133 mg, 2.2 equiv) and acetonitrile (2 mL), and the resulting solution was placed under a nitrogen atmosphere. The reaction flask was immersed in a preheated 85 °C oil bath, and stirring. The reaction was monitored by TLC analysis, and upon consumption of the **[D$_2$]-2b** (10–30 min), the reaction mixture was cooled to room temperature and concentrated by under vacuum by rotary evaporation. The resulting crude product mixture was purified by silica gel chromatography to provide **[D$_2$]-3b** in 79% yield (*E/Z*=1/2). **(*E*)-[D$_2$]-3b:** ^{1}H NMR (300 MHz, $CDCl_3$) δ 7.67 – 7.48 (m, 4H), 7.52 – 7.30 (m, 5H), 6.94 (s, 1H). ***(Z)*-[D$_2$]-3b:** ^{1}H NMR (300 MHz, $CDCl_3$) δ 7.73 (d, *J* = 8.0 Hz, 2H), 7.60 (d, *J* = 7.9 Hz, 4H), 7.44 (t, *J* = 7.5 Hz, 2H), 7.40 – 7.30 (m, 1H), 6.86 (s, 1H).

1. Z. Zhao, L. Racicot and G. K. Murphy, *Angew. Chem., Int. Ed.*, 2017, **56**, 11620.
2. J. An, X. Tang, J. Moore, W. Lewis and R. M. Denton, *Tetrahedron*, 2013, **69**, 8769.
3. J. Liu, Z. Han, X. Wang, Z. Wang and K. Ding, *J. Am. Chem. Soc.*, 2015, **137**, 15346.
4. (a) M. Bergeron, T. Johnson and J. F. Paquin, *Angew. Chem.*, 2011, **123**, 11308; (b) J. Yang, A. Mao, Z. Yue, W. Zhu, X. Luo, C. Zhu, Y. Xiao and J. Zhang, *Chem. Commun.*, 2015, **51**, 8326.
5. J. Liu, Q. Liu, R. Franke, R. Jackstell and M. Beller, *J. Am. Chem. Soc.*, 2015, **137**, 8556.
6. X. Yang, Y. She, Y. Chong, H. Zhai, H. Zhu, B. Chen, G. Huang and R. Yan, *Adv. Synth. Catal.*, 2016, **358**, 3130.
7. S. Ma and A. Zhang, *J. Org. Chem.*, 1998, **63**, 9601.
8. S. Yamazaki, Y. Yamamoto, Y. Fukushima, M. Takebayashi, T. Ukai and Y. Mikata, *J. Org. Chem.*, 2010, **75**, 5216.
9. G. Zhang, T. Xiong, Z. Wang, G. Xu, X. Wang and Q. Zhang, *Angew. Chem., Int. Ed.*, 2015, **54**, 12649.
10. S. Fortier, J. R. Walensky, G. Wu and T. W. Hayton, *J. Am. Chem. Soc.*, 2011, **133**, 6894.
11. L. Liu, J.; Nie, M.; Zhou, Q.; Gao, S.; Jiang, W.; Chung, L. W.; Tang, W.; Ding, K., *Chemical Science* **2017**, *8*, 5161-5165.
12. Laali, K. K.; Nandi, G. C.; Bunge, S. D., *Tetrahedron Lett.* **2014**, *55*, 2401-2405.

^{1}H NMR, ^{19}F and ^{13}C NMR spectra:

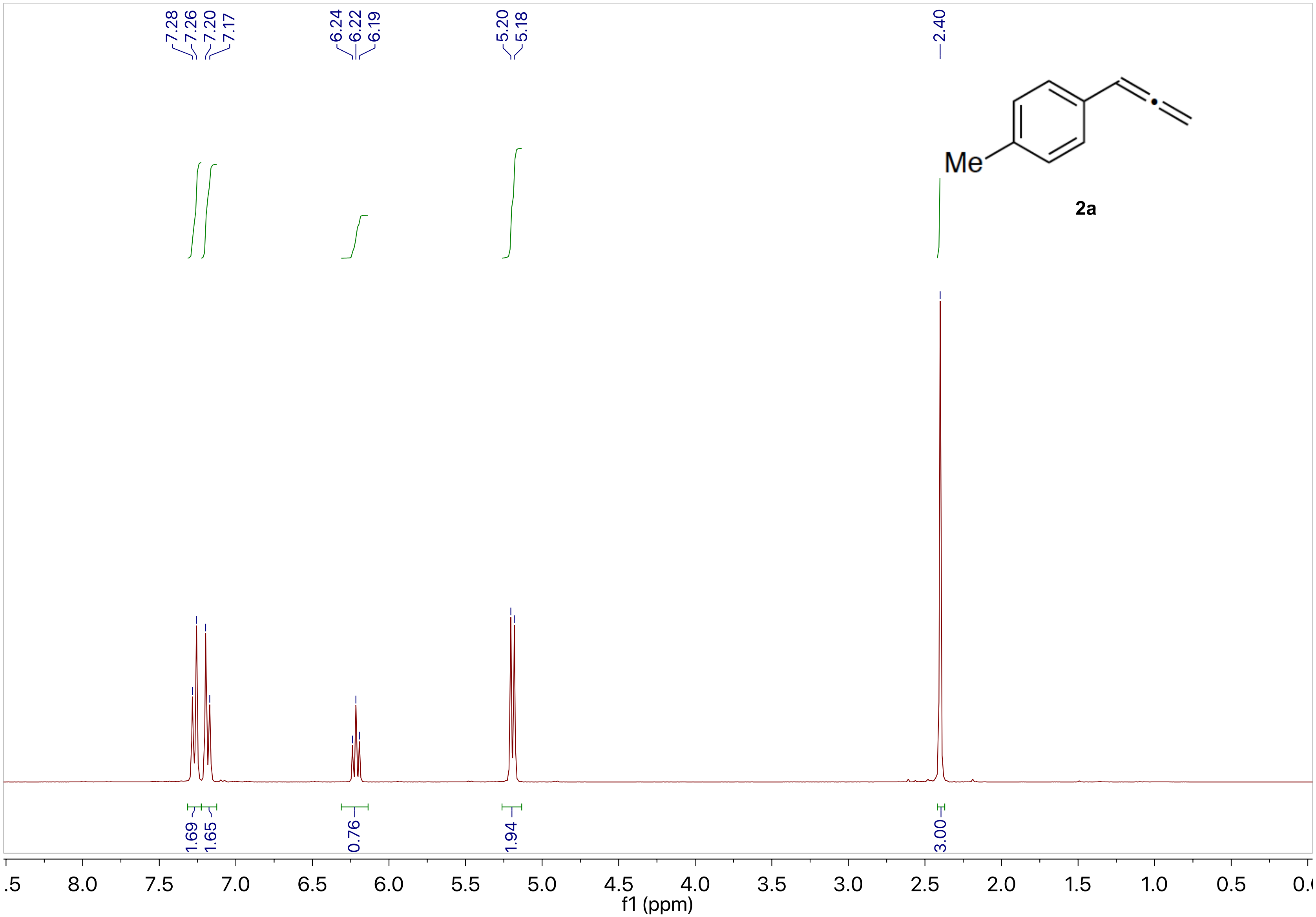
7.28
7.26
7.20
7.17
6.24
6.22
6.19
5.20
5.18
2.40
Me
2a
1.69
1.65
0.76
1.94
3.00
.5
8.0
7.5
7.0
6.5
6.0
5.5
5.0
4.5
4.0
3.5
3.0
2.5
2.0
1.5
1.0
0.5
f1 (ppm)

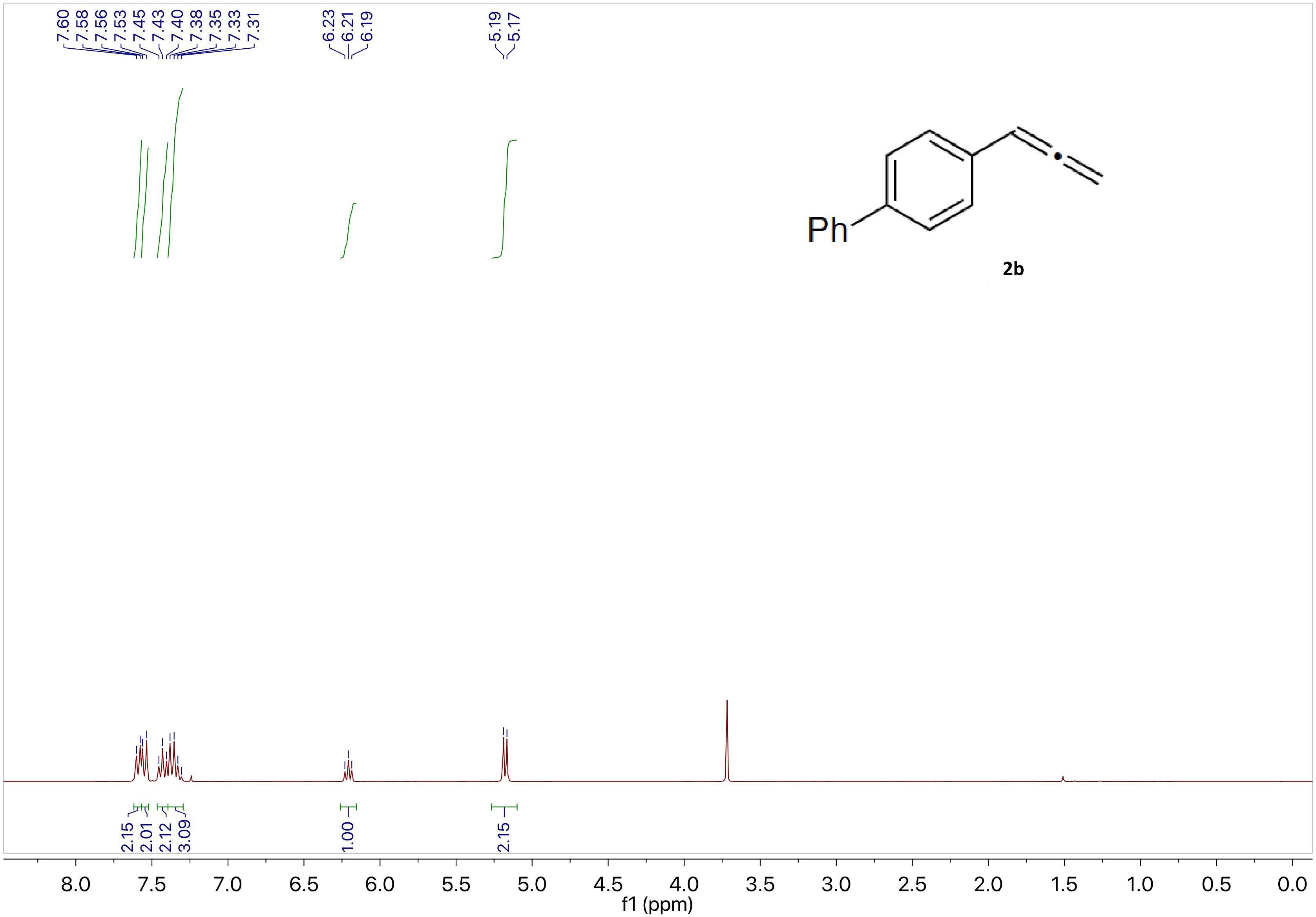
7.60
7.58
7.56
7.53
7.45
7.43
7.40
7.38
7.35
7.33
7.31
6.23
6.21
6.19
5.19
5.17
Ph
2.15
2.01
2.12
3.09
1.00
2.15
8.0
7.5
7.0
6.5
6.0
5.5
5.0
4.5
4.0
3.5
3.0
2.5
2.0
1.5
1.0
0.5
0.0
f1 (ppm)

2b

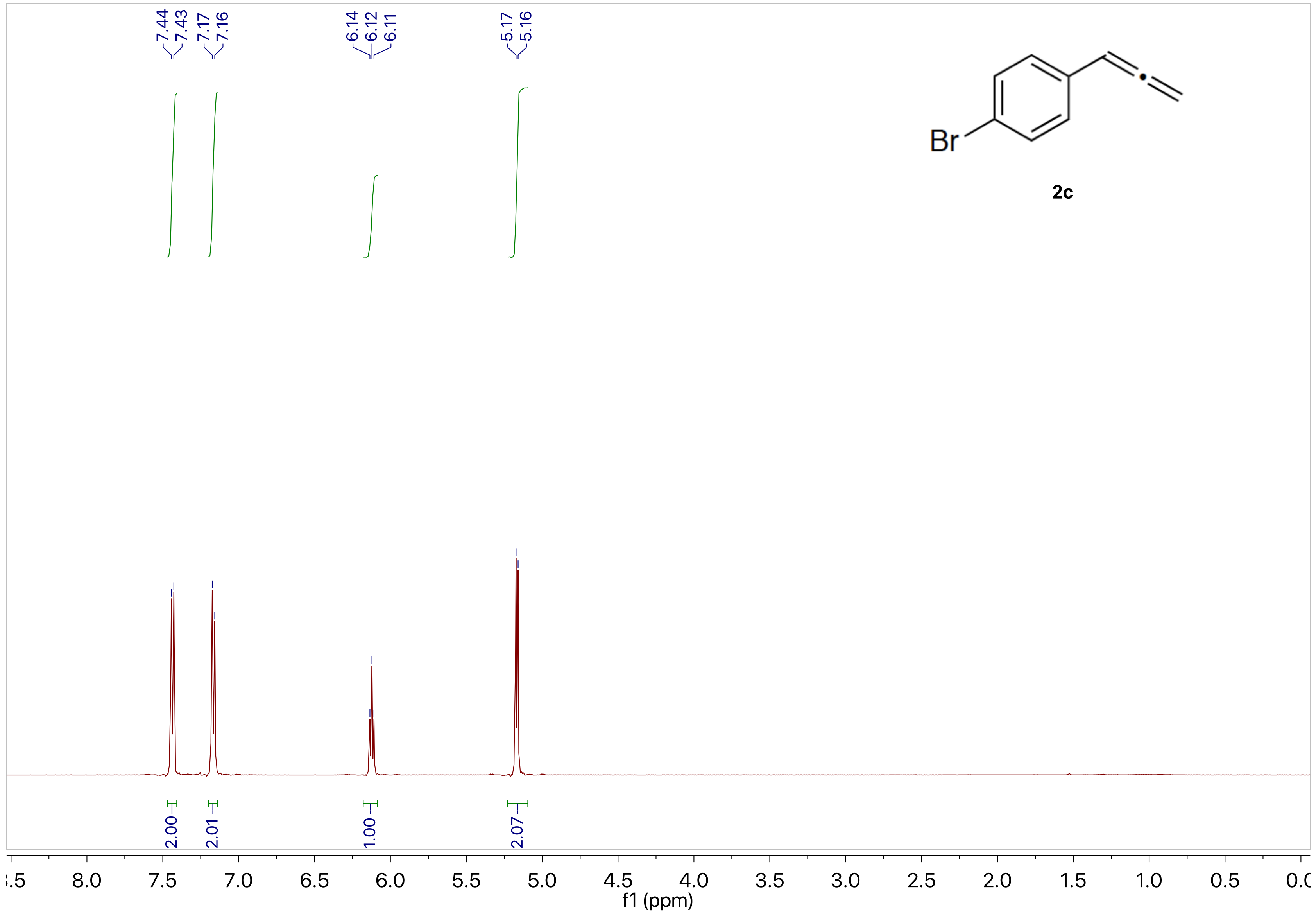

7.44
7.43
7.17
7.16
6.14
6.12
6.11
5.17
5.16
Br
2c
2.00
2.01
1.00
2.07
f1 (ppm)

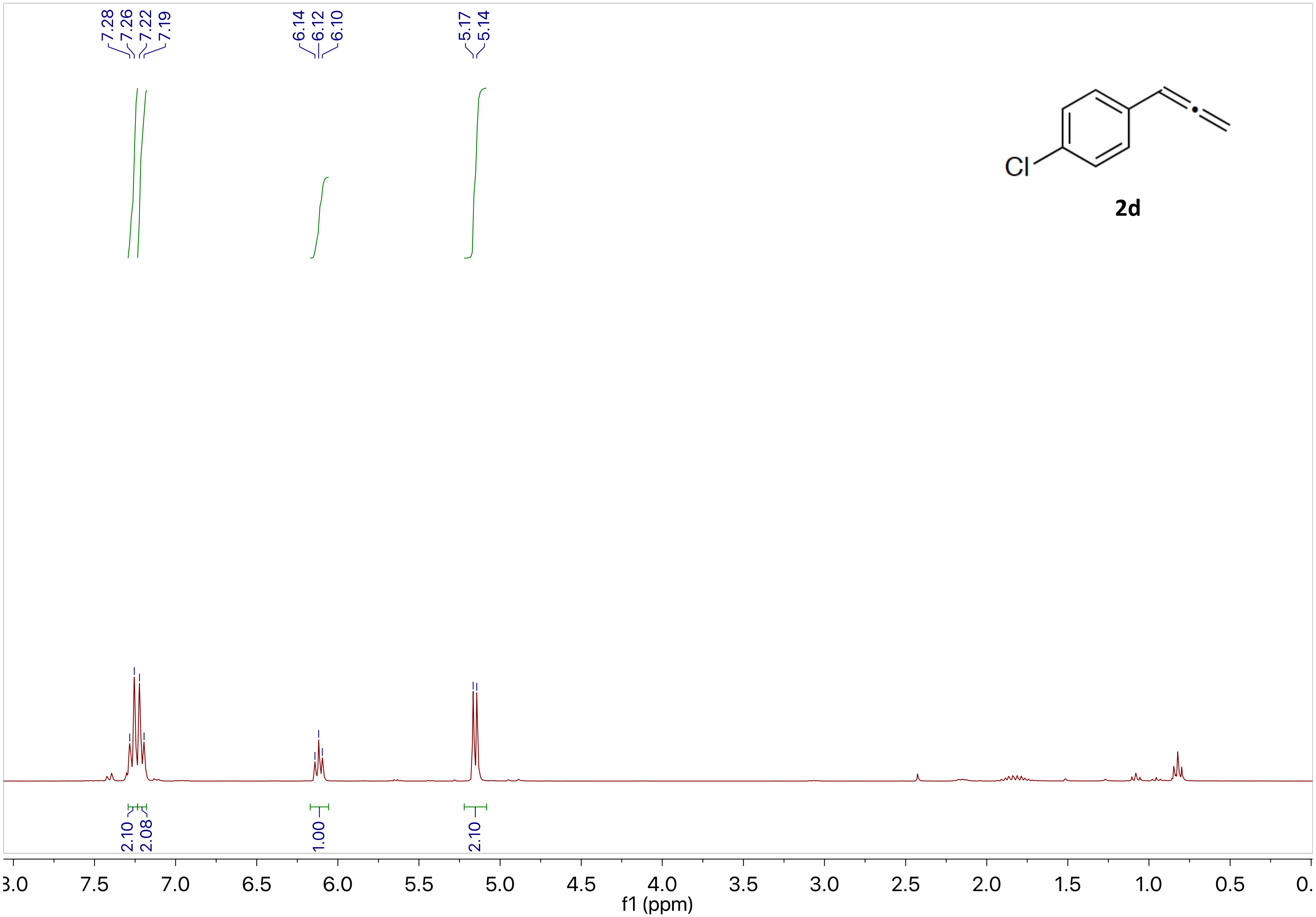
7.28
7.26
7.22
7.19
6.14
6.12
6.10
5.17
5.14
Cl
2d
2.10
2.08
1.00
2.10
8.0
7.5
7.0
6.5
6.0
5.5
5.0
4.5
4.0
3.5
3.0
2.5
2.0
1.5
1.0
0.5
0.
f1 (ppm)

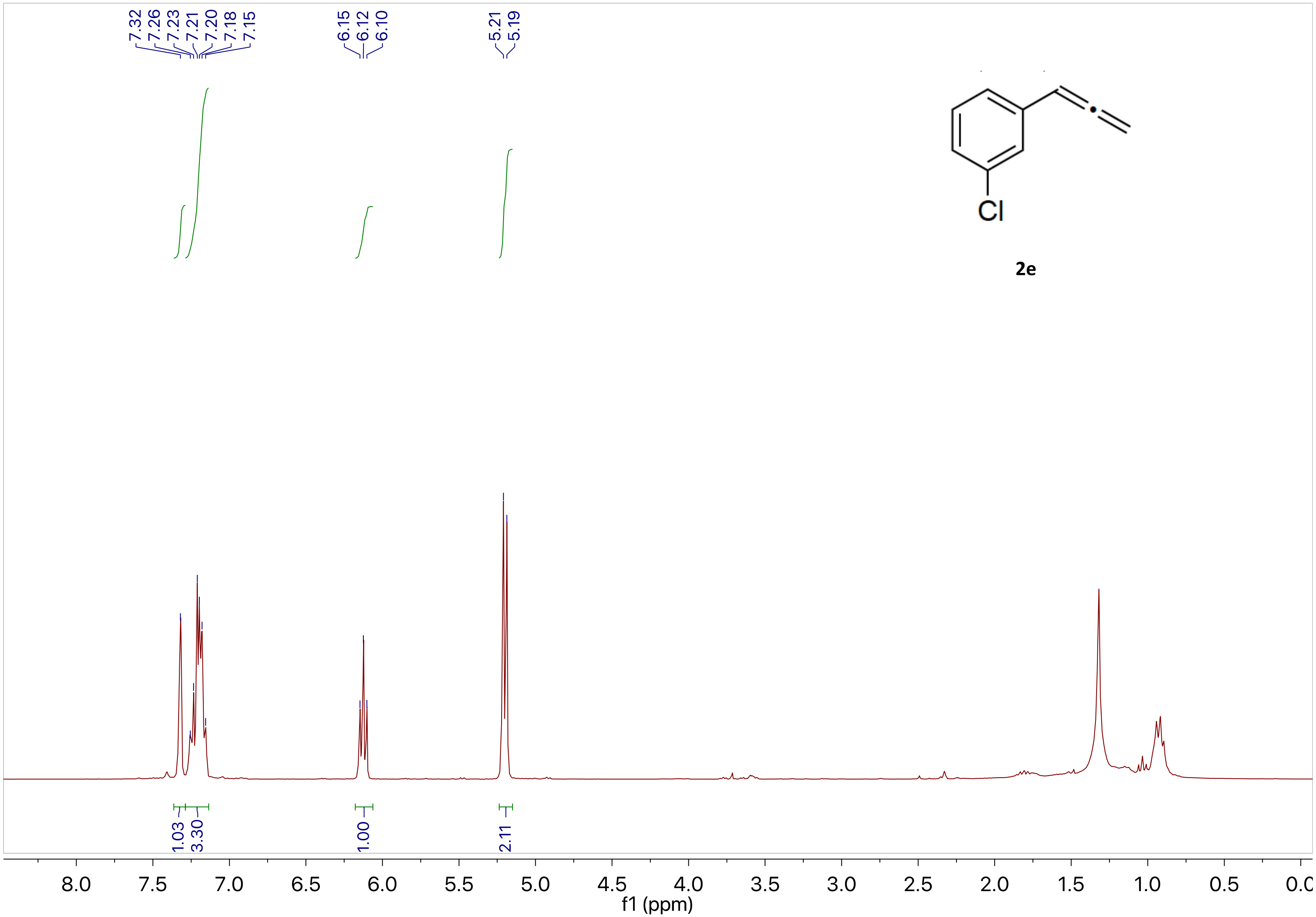

7.32
7.26
7.23
7.21
7.20
7.18
7.15
6.15
6.12
6.10
5.21
5.19
Cl
2e
1.03
3.30
1.00
2.11
8.0
7.5
7.0
6.5
6.0
5.5
5.0
4.5
4.0
3.5
3.0
2.5
2.0
1.5
1.0
0.5
f1 (ppm)

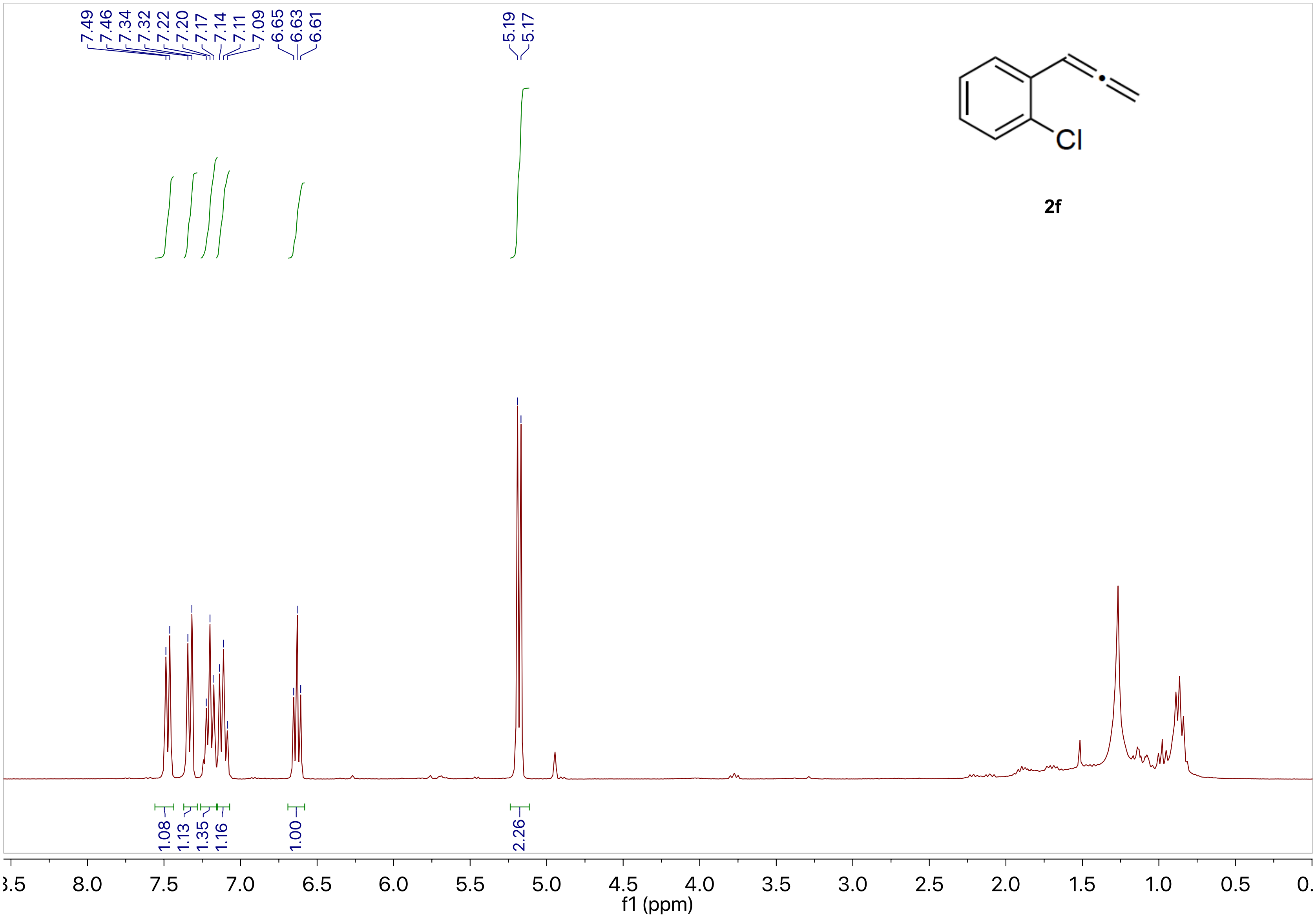
7.49
7.46
7.34
7.32
7.22
7.20
7.17
7.14
7.11
7.09
6.65
6.63
6.61
5.19
5.17
Cl
2f
1.08
1.13
1.35
1.16
1.00
2.26
8.5
8.0
7.5
7.0
6.5
6.0
5.5
5.0
4.5
4.0
3.5
3.0
2.5
2.0
1.5
1.0
0.5
0.
f1 (ppm)

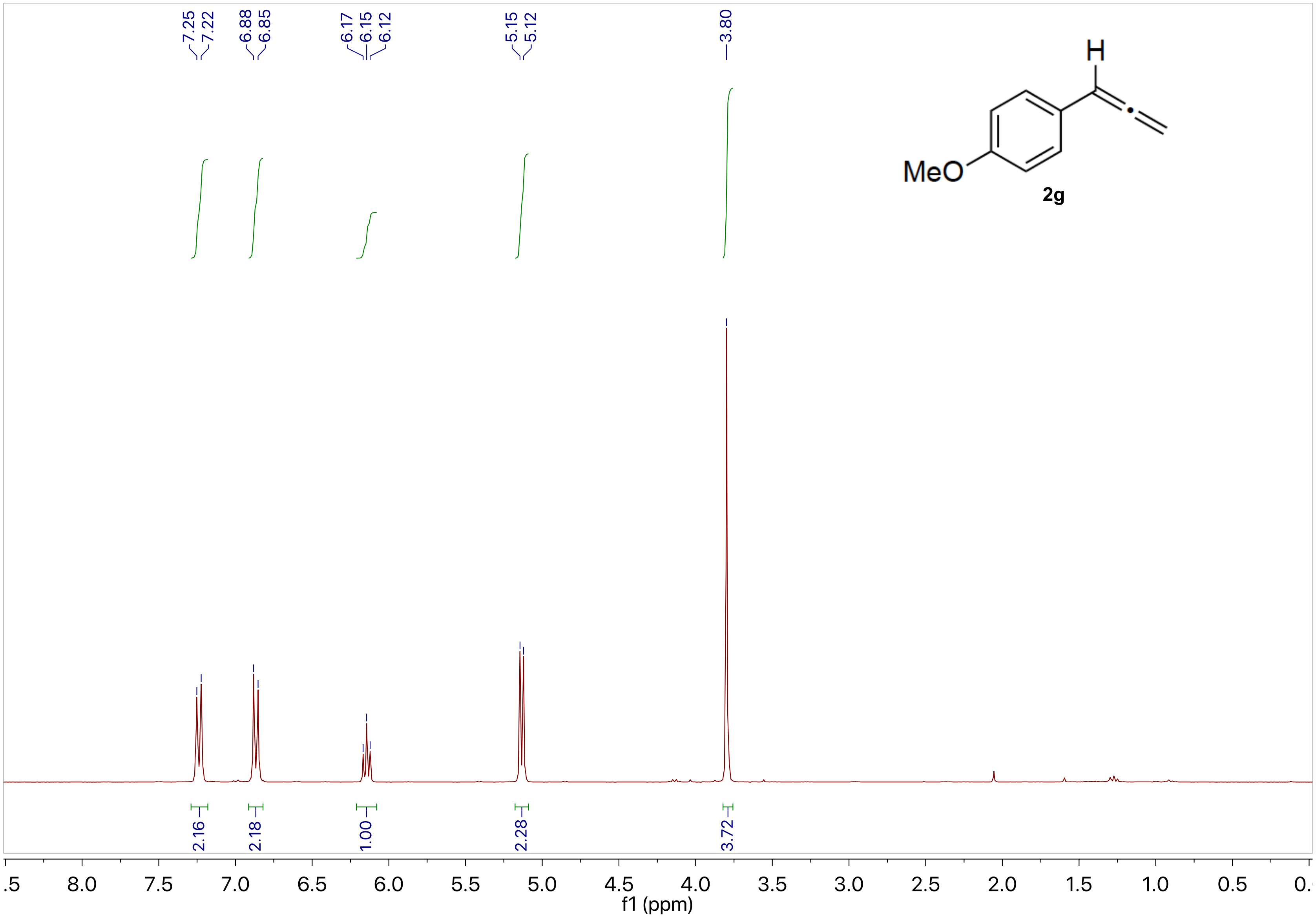
7.25
7.22
6.88
6.85
6.17
6.15
6.12
5.15
5.12
3.80
MeO
H
2g
2.16
2.18
1.00
2.28
3.72
.5
8.0
7.5
7.0
6.5
6.0
5.5
5.0
4.5
4.0
3.5
3.0
2.5
2.0
1.5
1.0
0.5
0.
f1 (ppm)

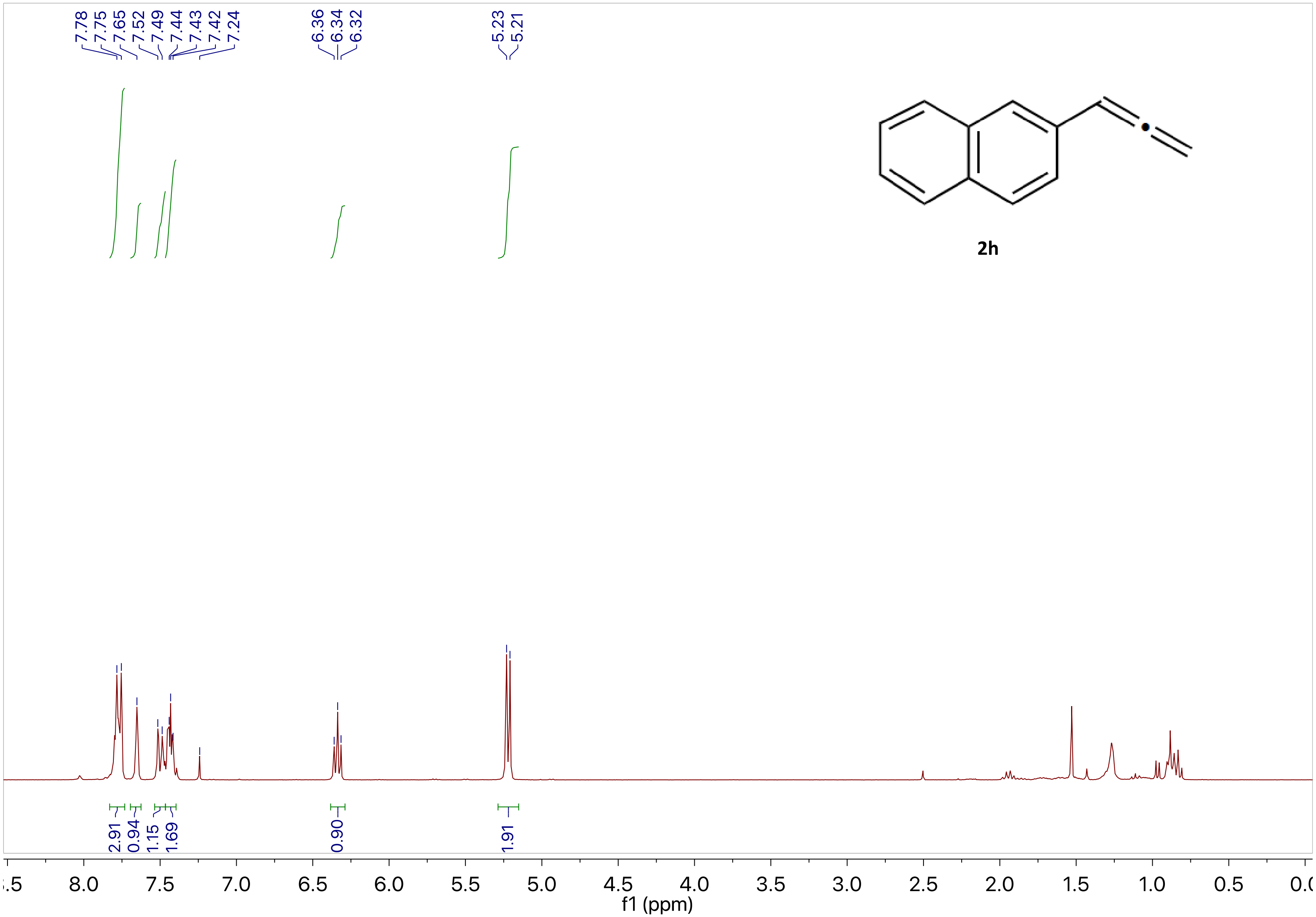
7.78
7.75
7.65
7.52
7.49
7.44
7.43
7.42
7.24
6.36
6.34
6.32
5.23
5.21
2.91
0.94
1.15
1.69
0.90
1.91
8.0
7.5
7.0
6.5
6.0
5.5
5.0
4.5
4.0
3.5
3.0
2.5
2.0
1.5
1.0
0.5
f1 (ppm)

2h

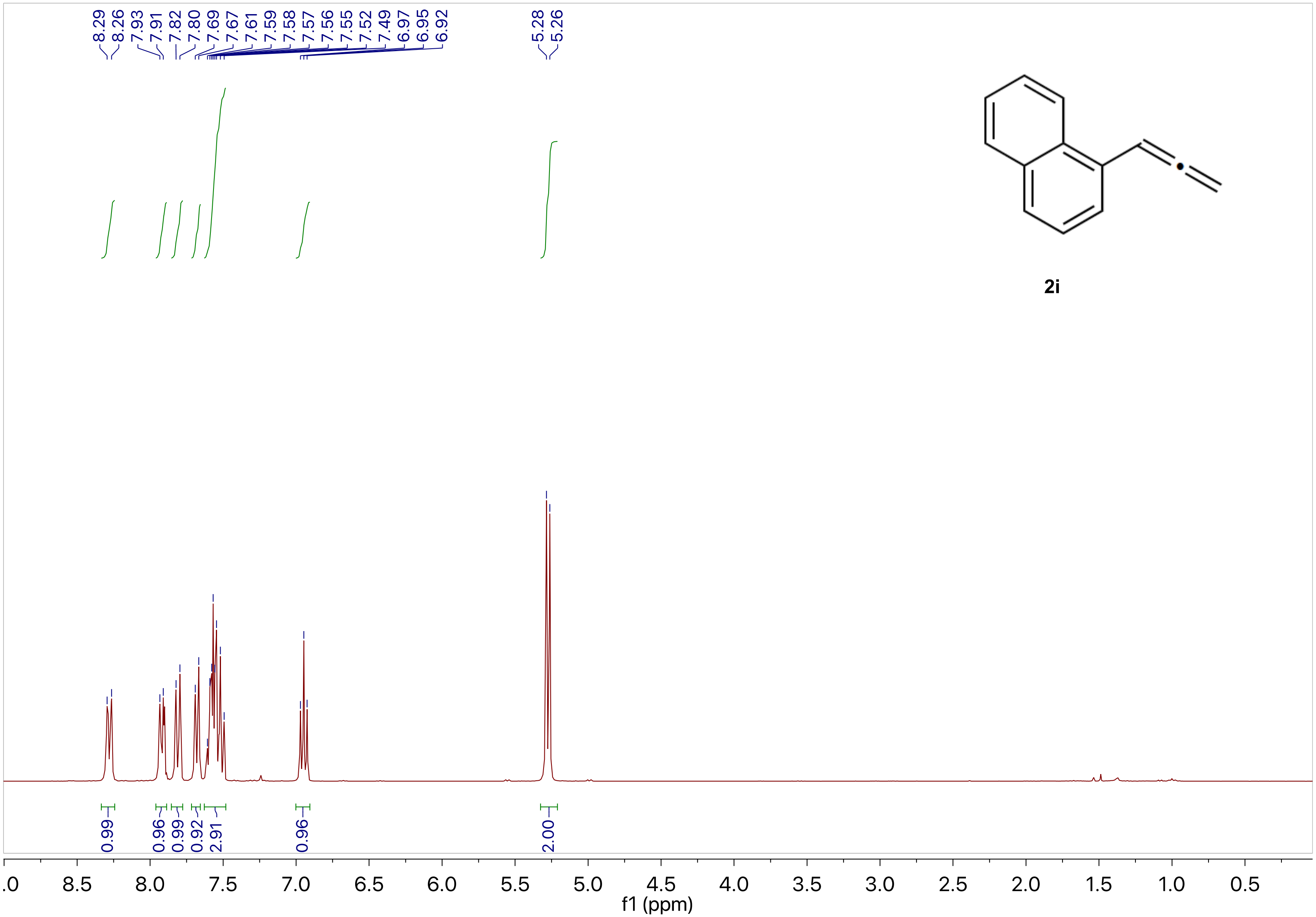

8.29
8.26
7.93
7.91
7.82
7.80
7.69
7.67
7.61
7.59
7.58
7.57
7.56
7.55
7.52
7.49
6.97
6.95
6.92
5.28
5.26
0.99
0.96
0.99
0.92
2.91
0.96
2.00
.0 8.5 8.0 7.5 7.0 6.5 6.0 5.5 5.0 4.5 4.0 3.5 3.0 2.5 2.0 1.5 1.0 0.5
f1 (ppm)

2i

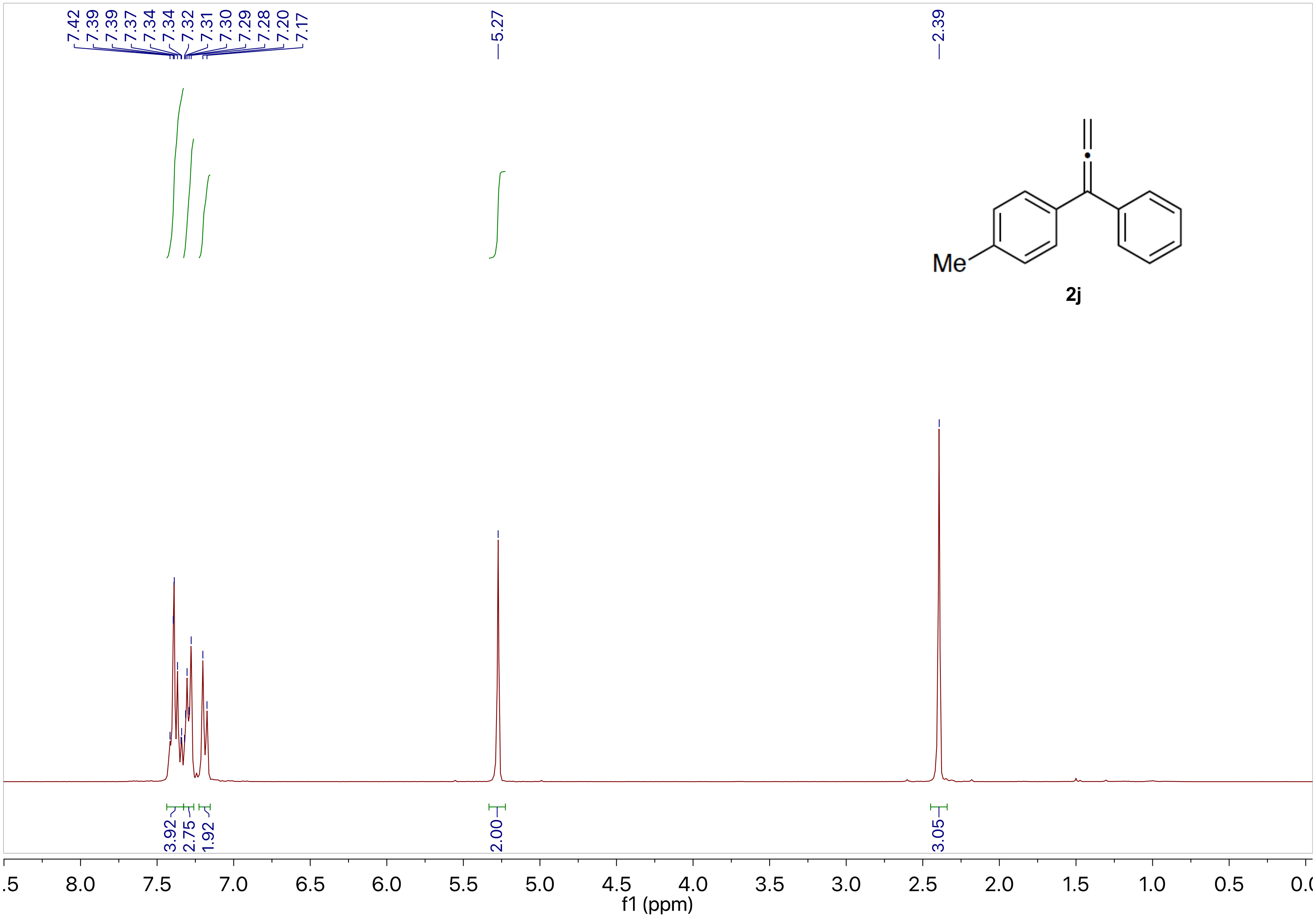
7.42
7.39
7.39
7.37
7.34
7.34
7.32
7.31
7.30
7.29
7.28
7.20
7.17
5.27
2.39
Me
2j
3.92
2.75
1.92
2.00
3.05
.5
8.0
7.5
7.0
6.5
6.0
5.5
5.0
4.5
4.0
3.5
3.0
2.5
2.0
1.5
1.0
0.5
f1 (ppm)

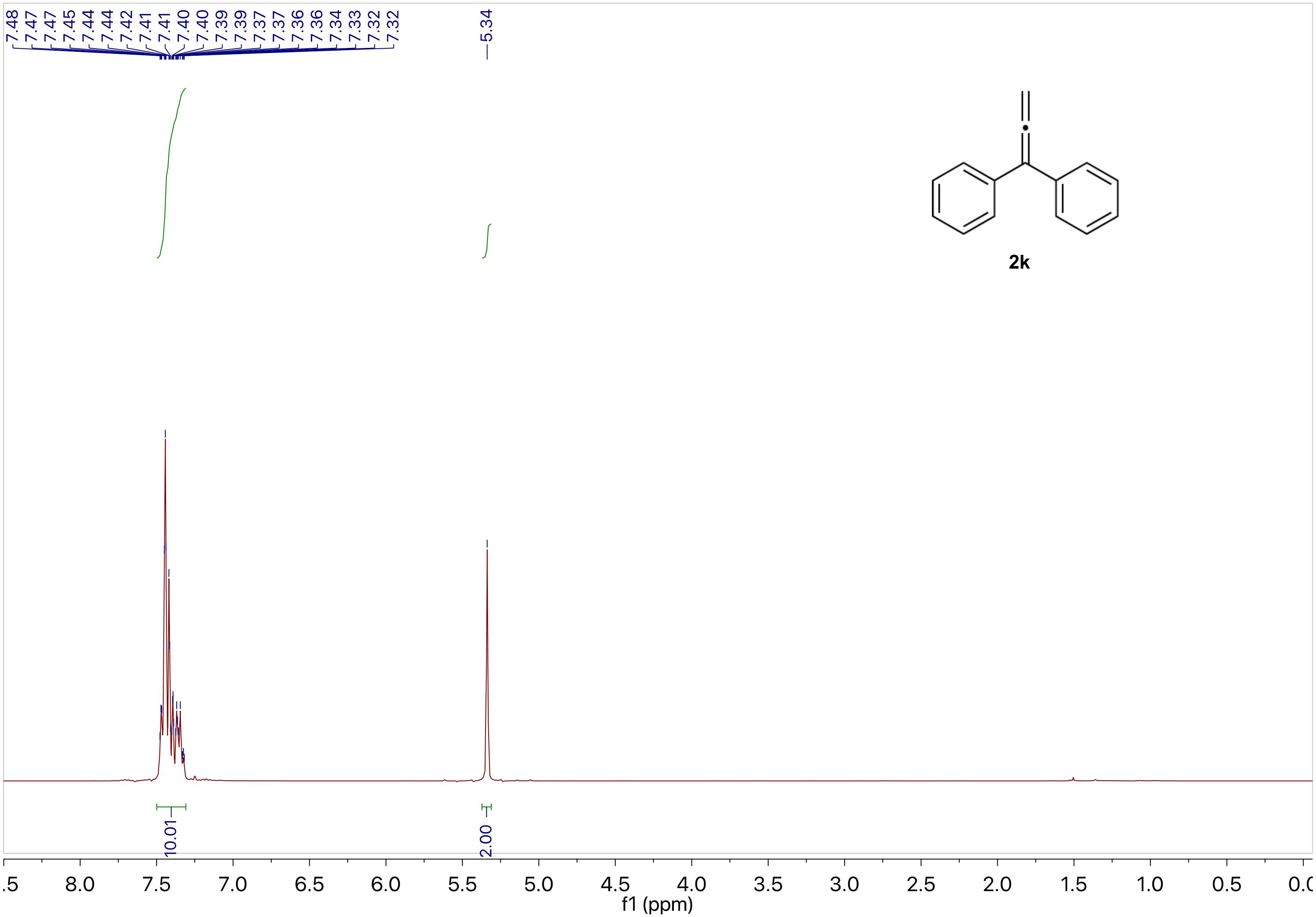
7.48
7.47
7.47
7.45
7.44
7.44
7.42
7.41
7.41
7.40
7.40
7.39
7.39
7.37
7.37
7.36
7.36
7.34
7.33
7.32
7.32
5.34
10.01
2.00
.5
8.0
7.5
7.0
6.5
6.0
5.5
5.0
4.5
4.0
3.5
3.0
2.5
2.0
1.5
1.0
0.5
0.0
f1 (ppm)

2k

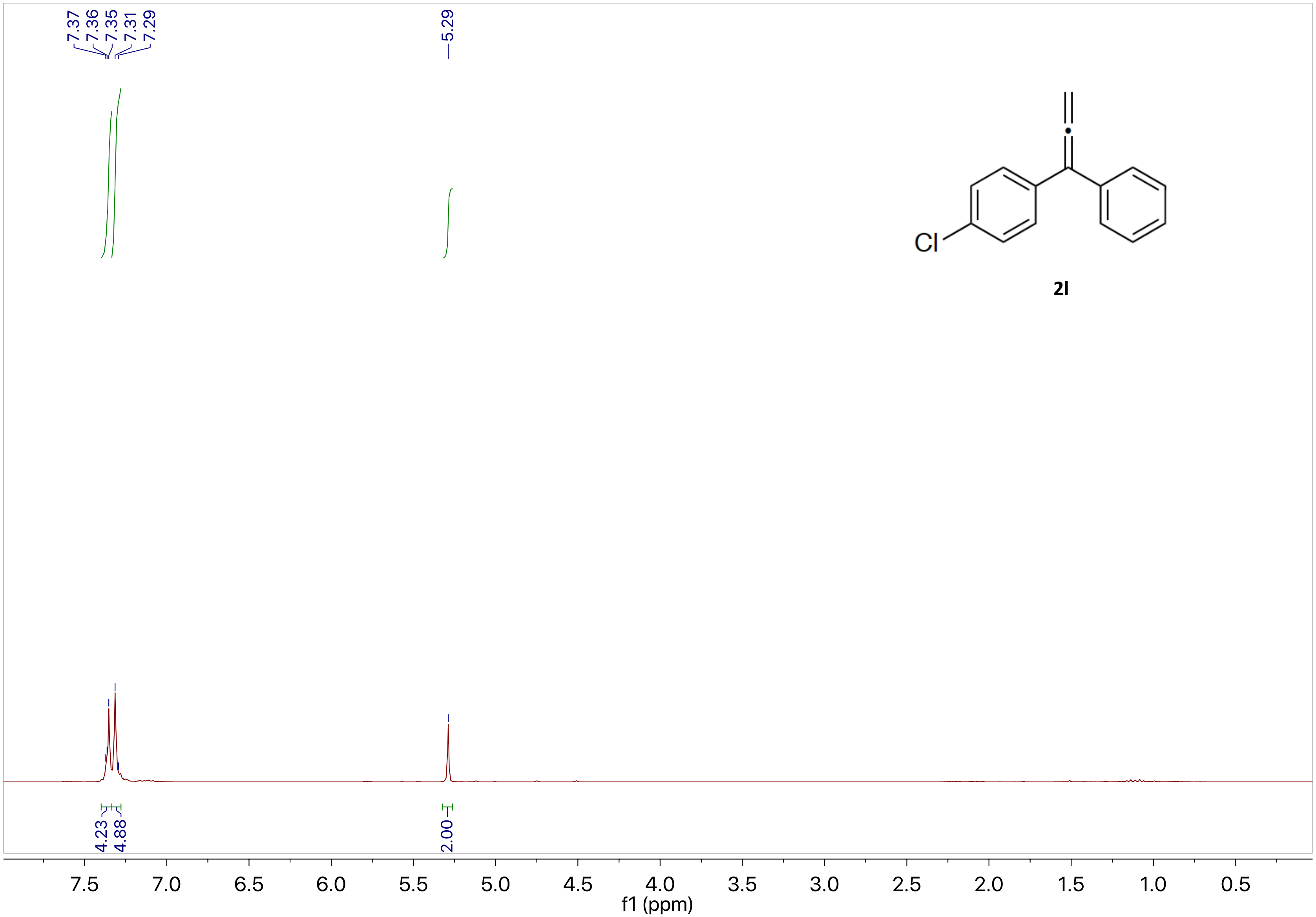
7.37
7.36
7.35
7.31
7.29
5.29
Cl
2l
4.23
4.88
2.00
7.5
7.0
6.5
6.0
5.5
5.0
4.5
4.0
3.5
3.0
2.5
2.0
1.5
1.0
0.5
f1 (ppm)

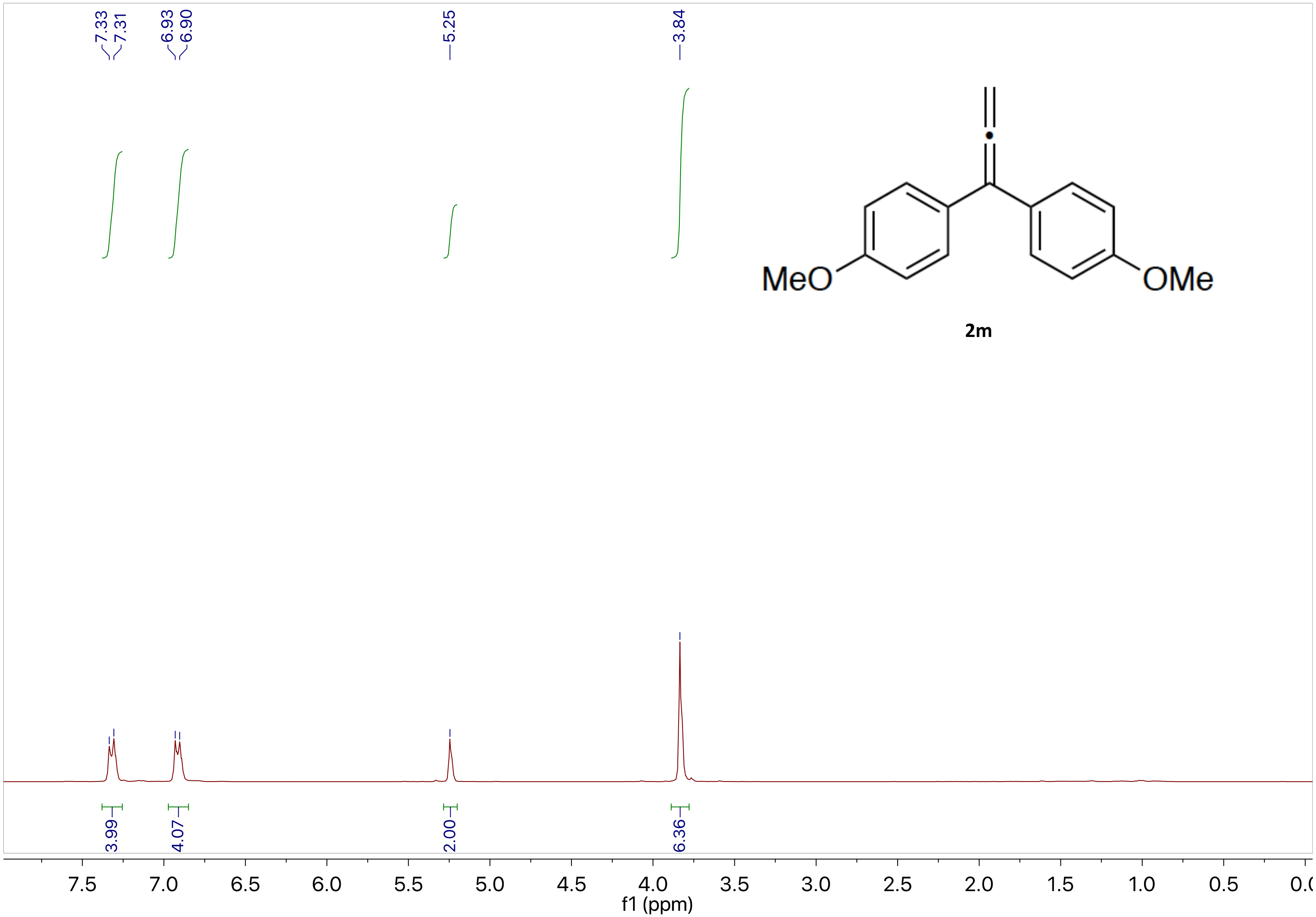
7.33
7.31
6.93
6.90
5.25
3.84
MeO
OMe
2m
3.99
4.07
2.00
6.36
7.5
7.0
6.5
6.0
5.5
5.0
4.5
4.0
3.5
3.0
2.5
2.0
1.5
1.0
0.5
f1 (ppm)

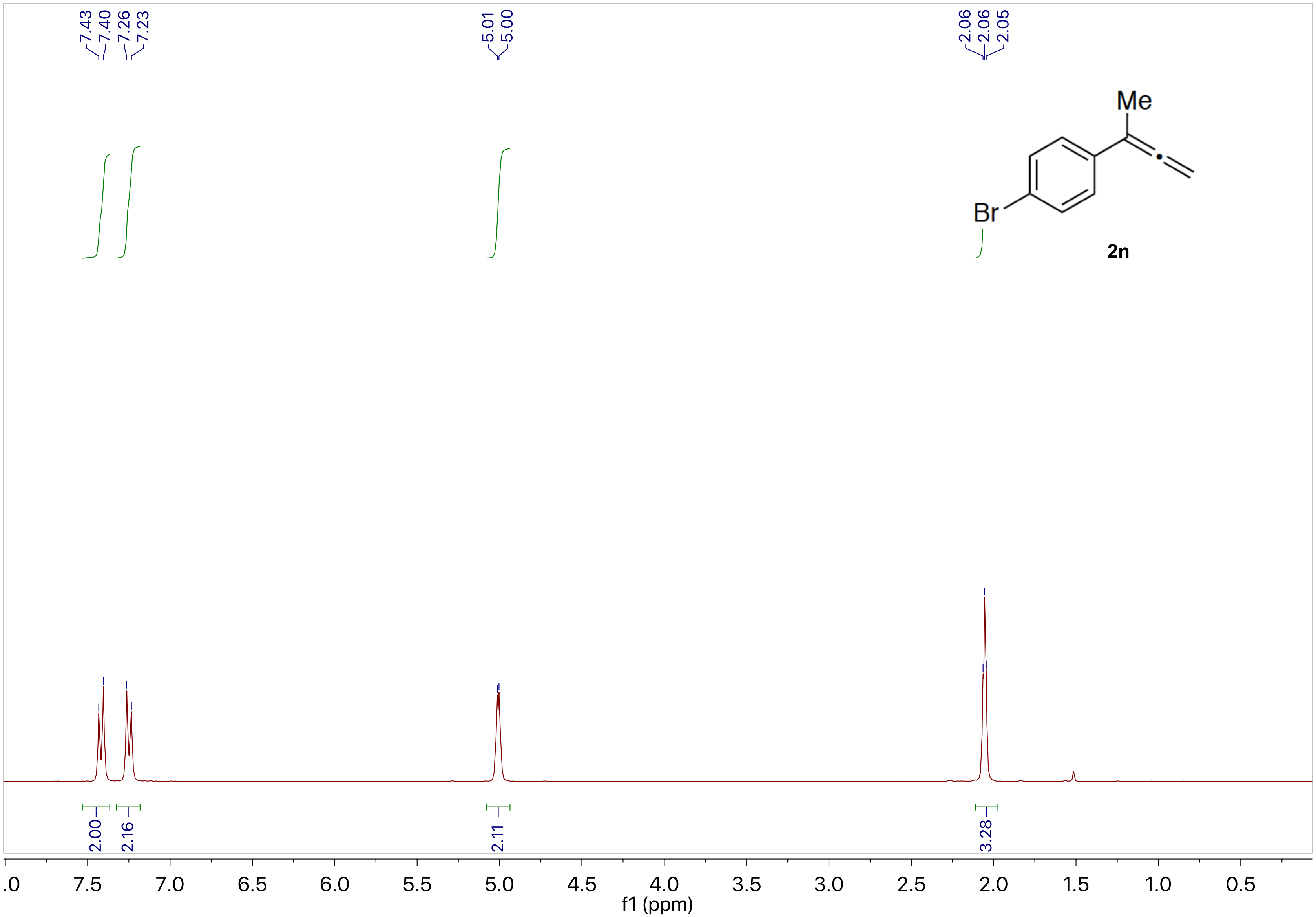
7.43
7.40
7.26
7.23
5.01
5.00
2.06
2.06
2.05
Me
Br
2n
2.00
2.16
2.11
3.28
.0
7.5
7.0
6.5
6.0
5.5
5.0
4.5
4.0
3.5
3.0
2.5
2.0
1.5
1.0
0.5
f1 (ppm)

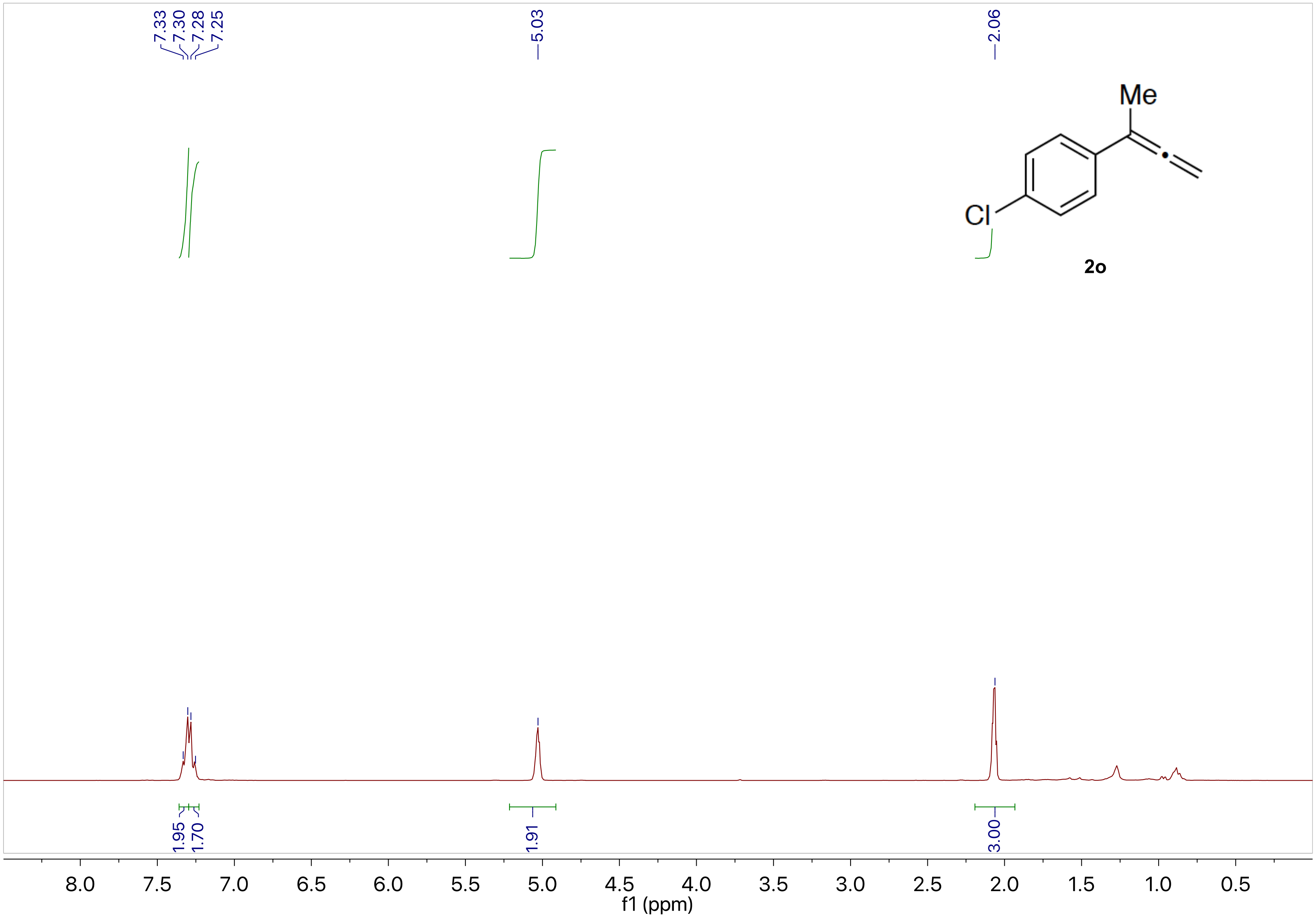
7.33
7.30
7.28
7.25
5.03
2.06
Me
Cl
2o
1.95
1.70
1.91
3.00
8.0
7.5
7.0
6.5
6.0
5.5
5.0
4.5
4.0
3.5
3.0
2.5
2.0
1.5
1.0
0.5
f1 (ppm)

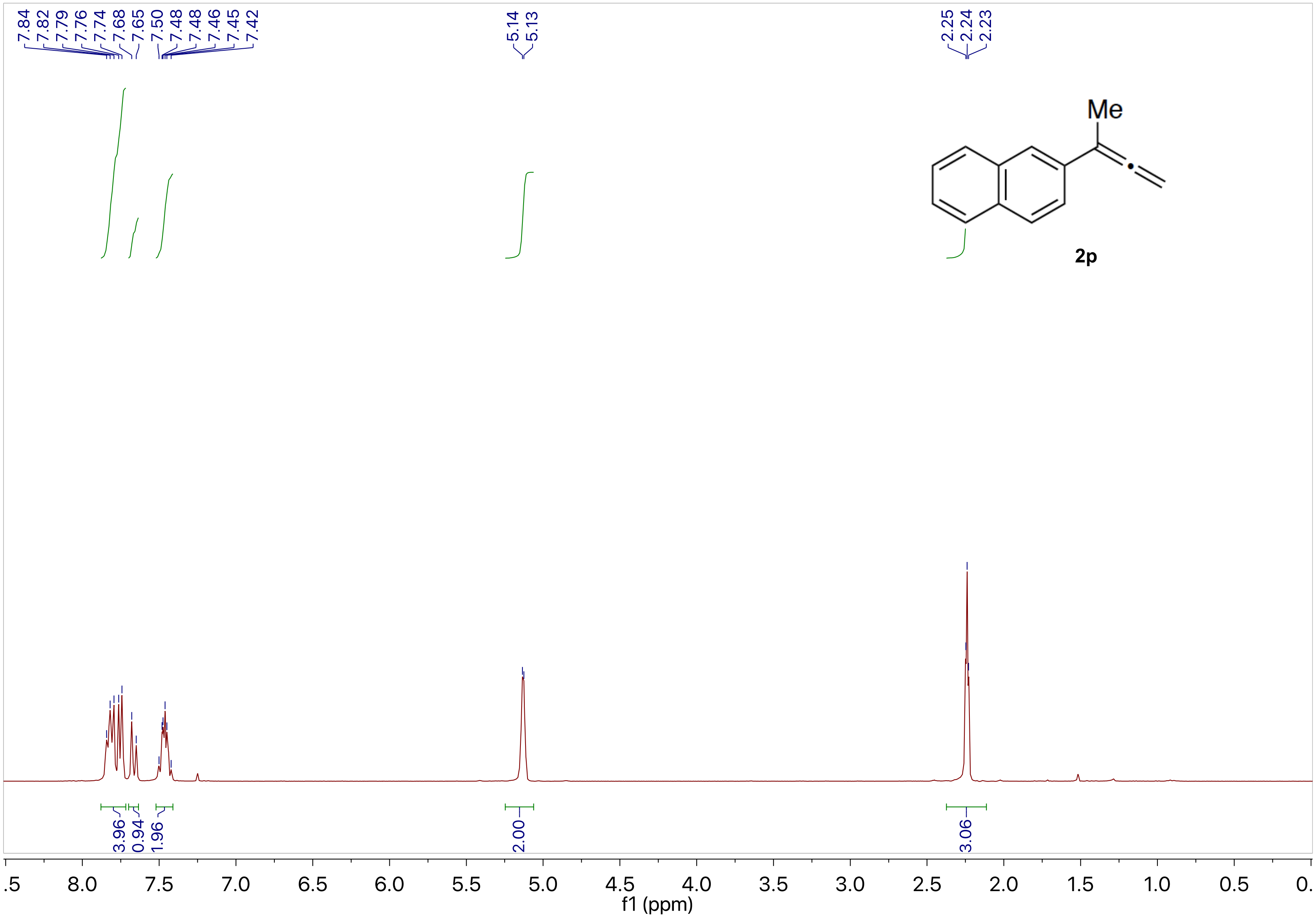

7.84
7.82
7.79
7.76
7.74
7.68
7.65
7.50
7.48
7.48
7.46
7.45
7.42
5.14
5.13
2.25
2.24
2.23
Me
3.96
0.94
1.96
2.00
3.06
.5
8.0
7.5
7.0
6.5
6.0
5.5
5.0
4.5
4.0
3.5
3.0
2.5
2.0
1.5
1.0
0.5
0.
f1 (ppm)

2p

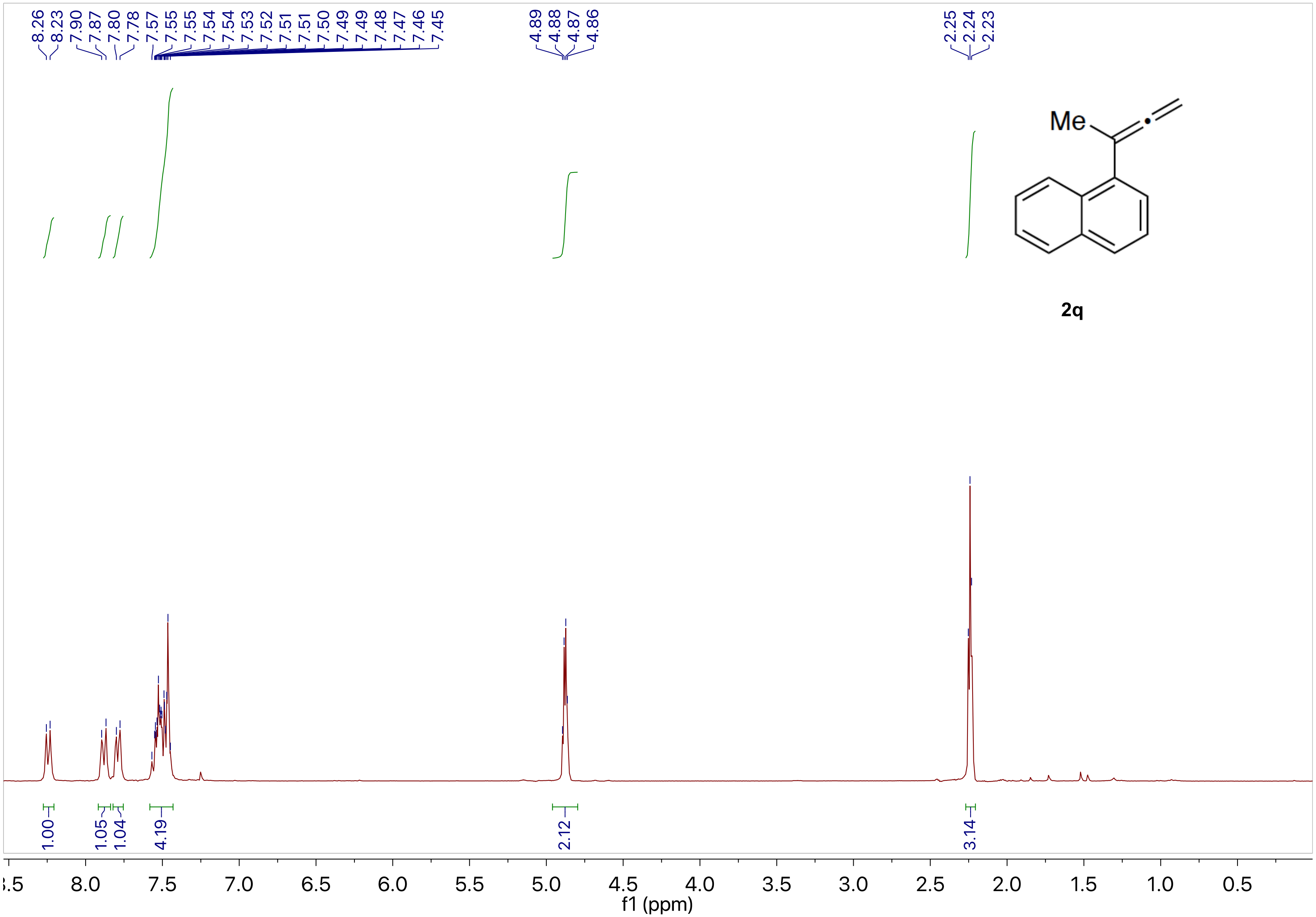

8.26
8.23
7.90
7.87
7.80
7.78
7.57
7.55
7.55
7.54
7.54
7.53
7.52
7.51
7.51
7.50
7.49
7.49
7.48
7.47
7.46
7.45
4.89
4.88
4.87
4.86
2.25
2.24
2.23
Me
1.00
1.05
1.04
4.19
2.12
3.14
8.0
7.5
7.0
6.5
6.0
5.5
5.0
4.5
4.0
3.5
3.0
2.5
2.0
1.5
1.0
0.5
f1 (ppm)

2q

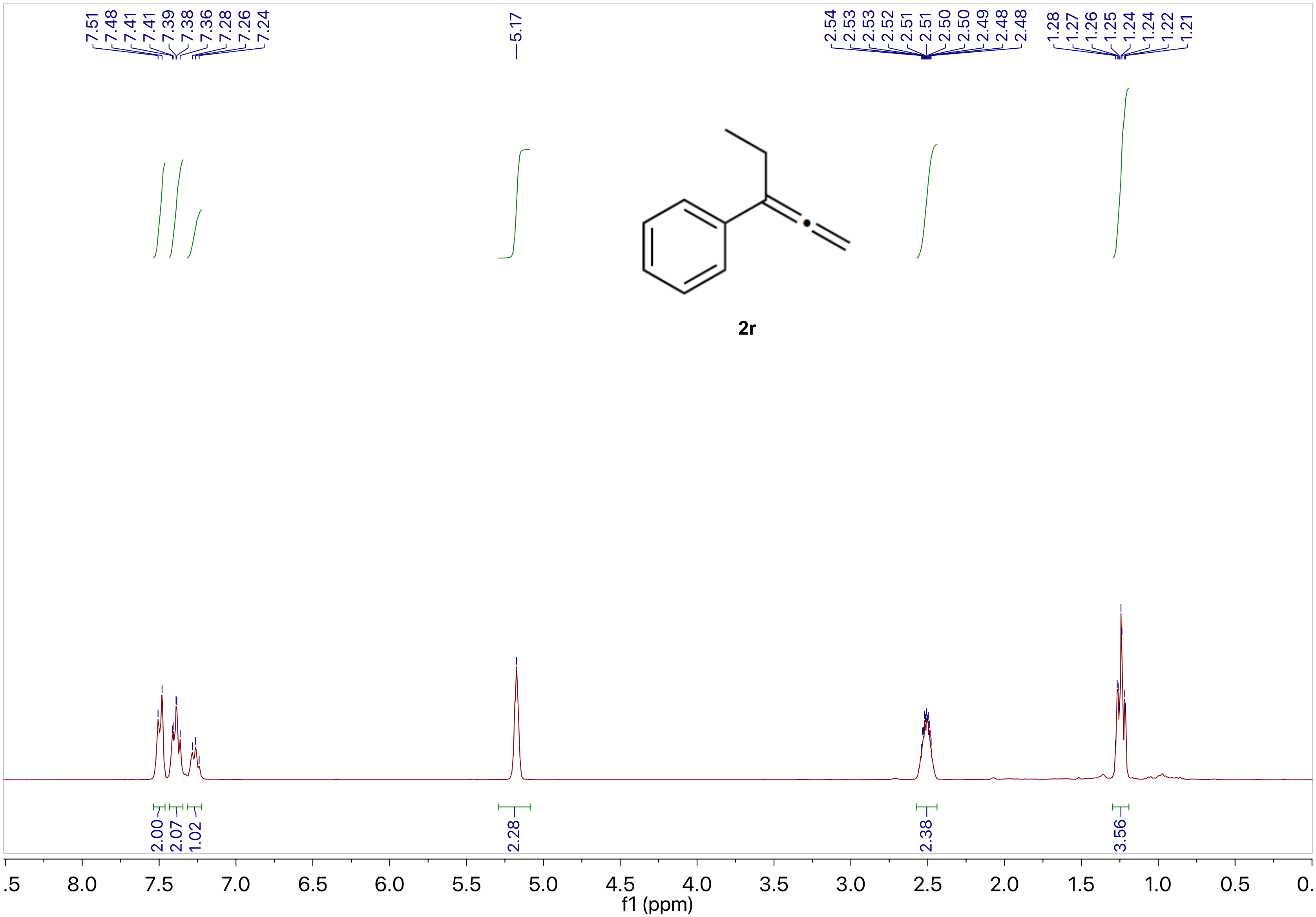
7.51
7.48
7.41
7.41
7.39
7.38
7.36
7.28
7.26
7.24
5.17
2.54
2.53
2.53
2.52
2.51
2.51
2.50
2.50
2.49
2.48
2.48
1.28
1.27
1.26
1.25
1.24
1.24
1.22
1.21
2.00
2.07
1.02
2.28
2.38
3.56
.5
8.0
7.5
7.0
6.5
6.0
5.5
5.0
4.5
4.0
3.5
3.0
2.5
2.0
1.5
1.0
0.5
0.
f1 (ppm)

2r

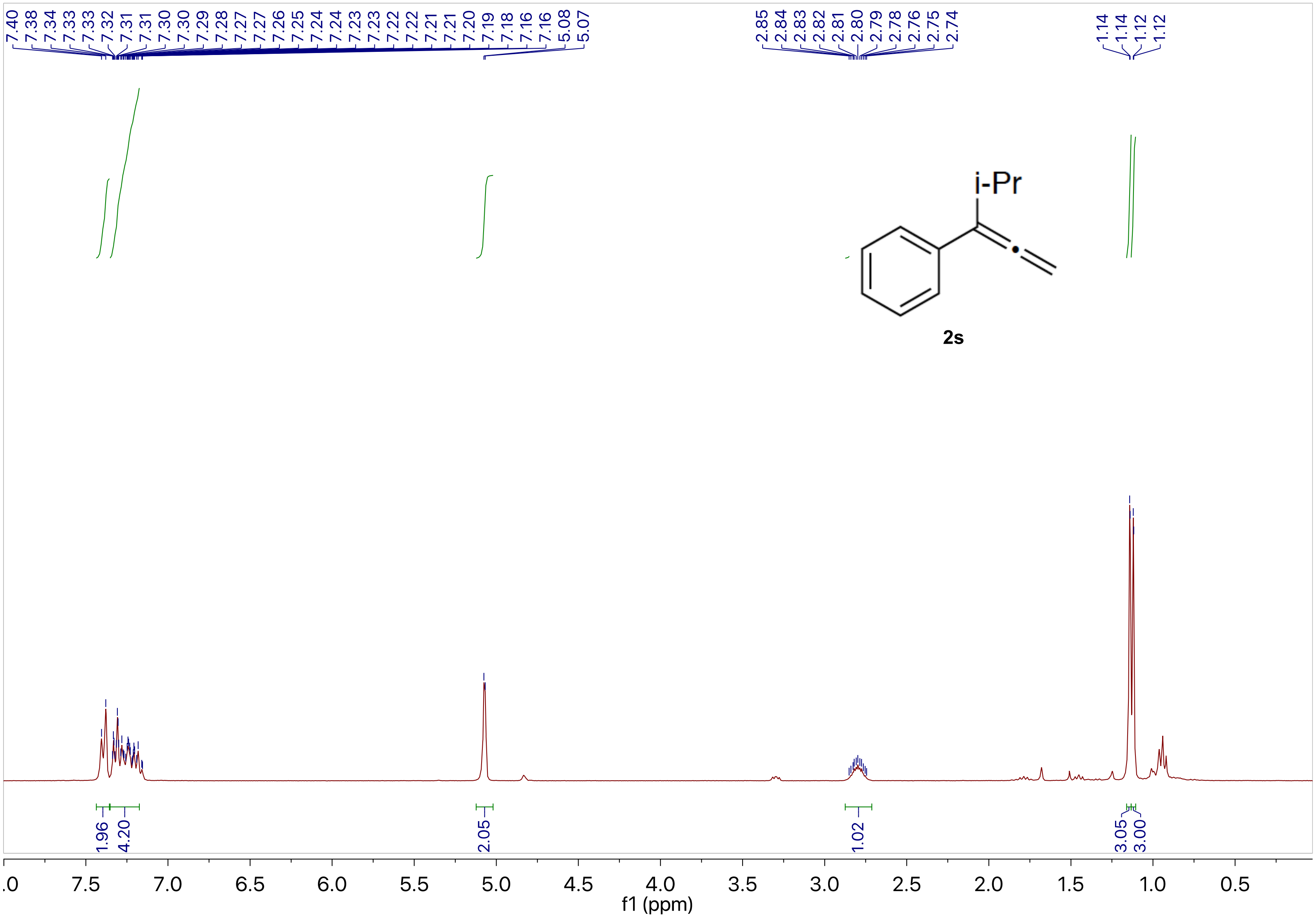
7.40
7.38
7.34
7.33
7.33
7.32
7.31
7.31
7.30
7.30
7.29
7.28
7.27
7.27
7.26
7.25
7.24
7.24
7.23
7.23
7.22
7.22
7.21
7.21
7.20
7.19
7.18
7.16
7.16
5.08
5.07
2.85
2.84
2.83
2.82
2.81
2.80
2.79
2.78
2.76
2.75
2.74
1.14
1.14
1.12
1.12
i-Pr
1.96
4.20
2.05
1.02
3.05
3.00
.0
7.5
7.0
6.5
6.0
5.5
5.0
4.5
4.0
3.5
3.0
2.5
2.0
1.5
1.0
0.5
f1 (ppm)

2s

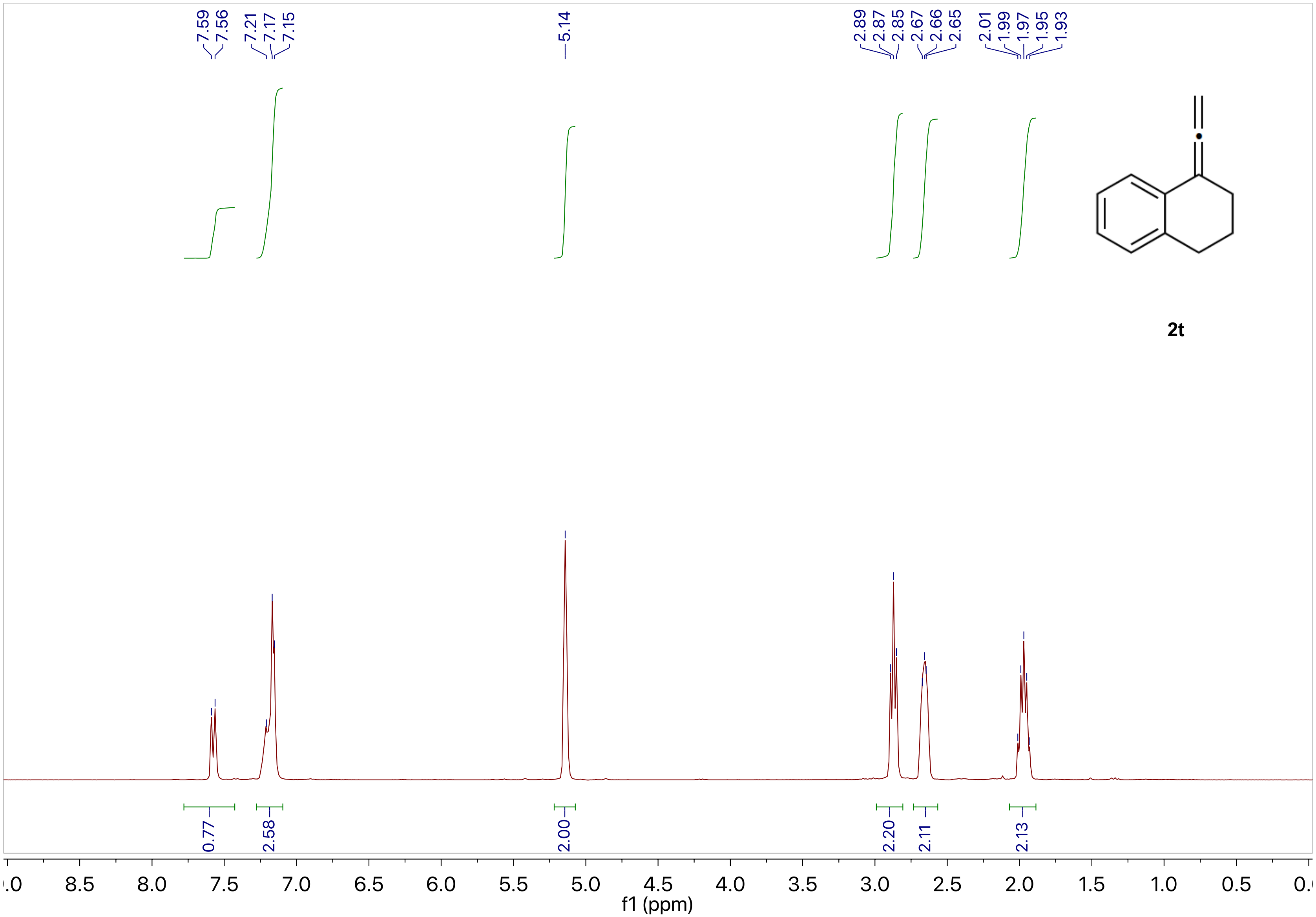
7.59
7.56
7.21
7.17
7.15
5.14
2.89
2.87
2.85
2.67
2.66
2.65
2.01
1.99
1.97
1.95
1.93
0.77
2.58
2.00
2.20
2.11
2.13
2t
8.5
8.0
7.5
7.0
6.5
6.0
5.5
5.0
4.5
4.0
3.5
3.0
2.5
2.0
1.5
1.0
0.5
f1 (ppm)

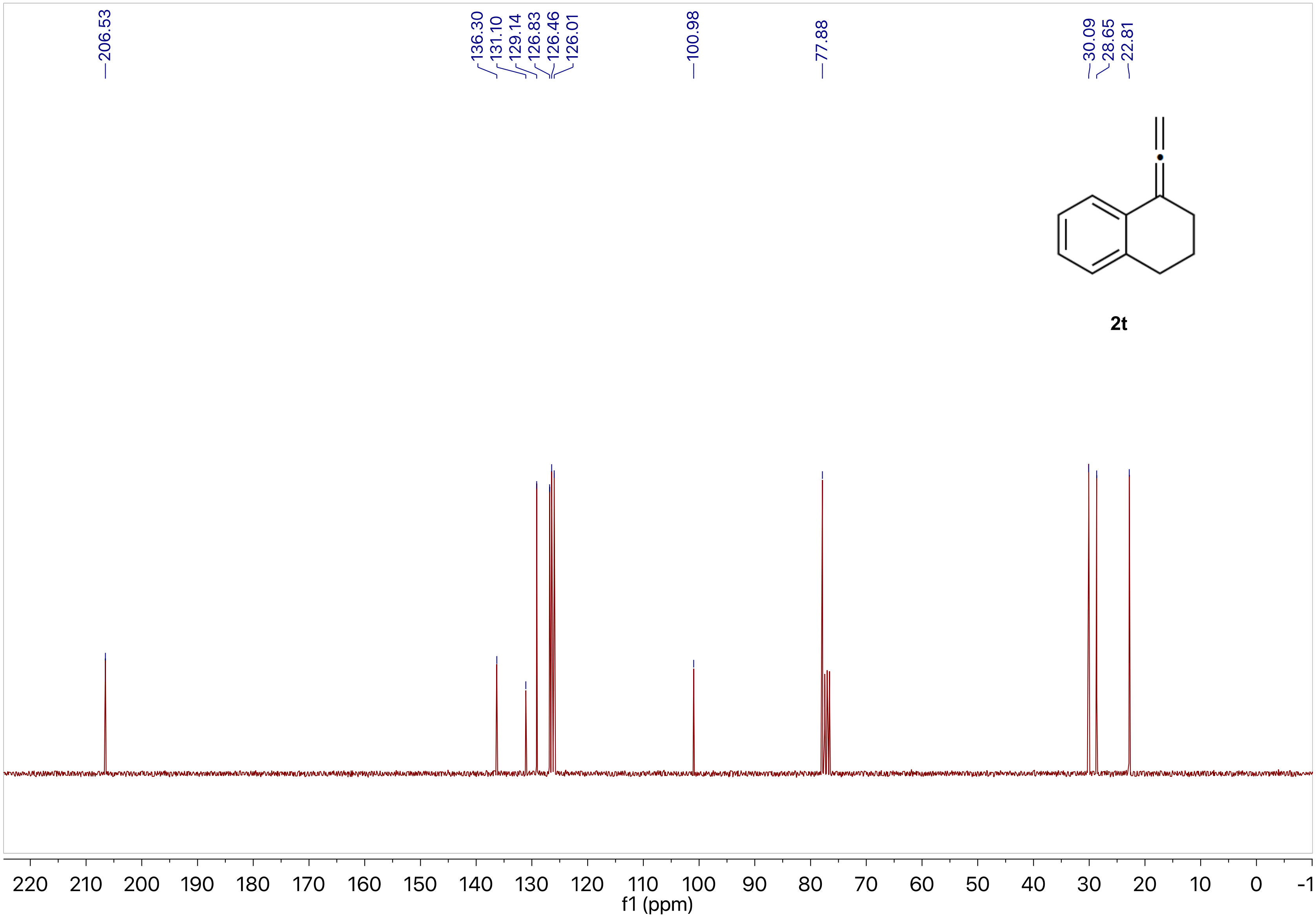
206.53
136.30
131.10
129.14
126.83
126.46
126.01
100.98
77.88
30.09
28.65
22.81
2t
220 210 200 190 180 170 160 150 140 130 120 110 100 90 80 70 60 50 40 30 20 10 0 -1
f1 (ppm)

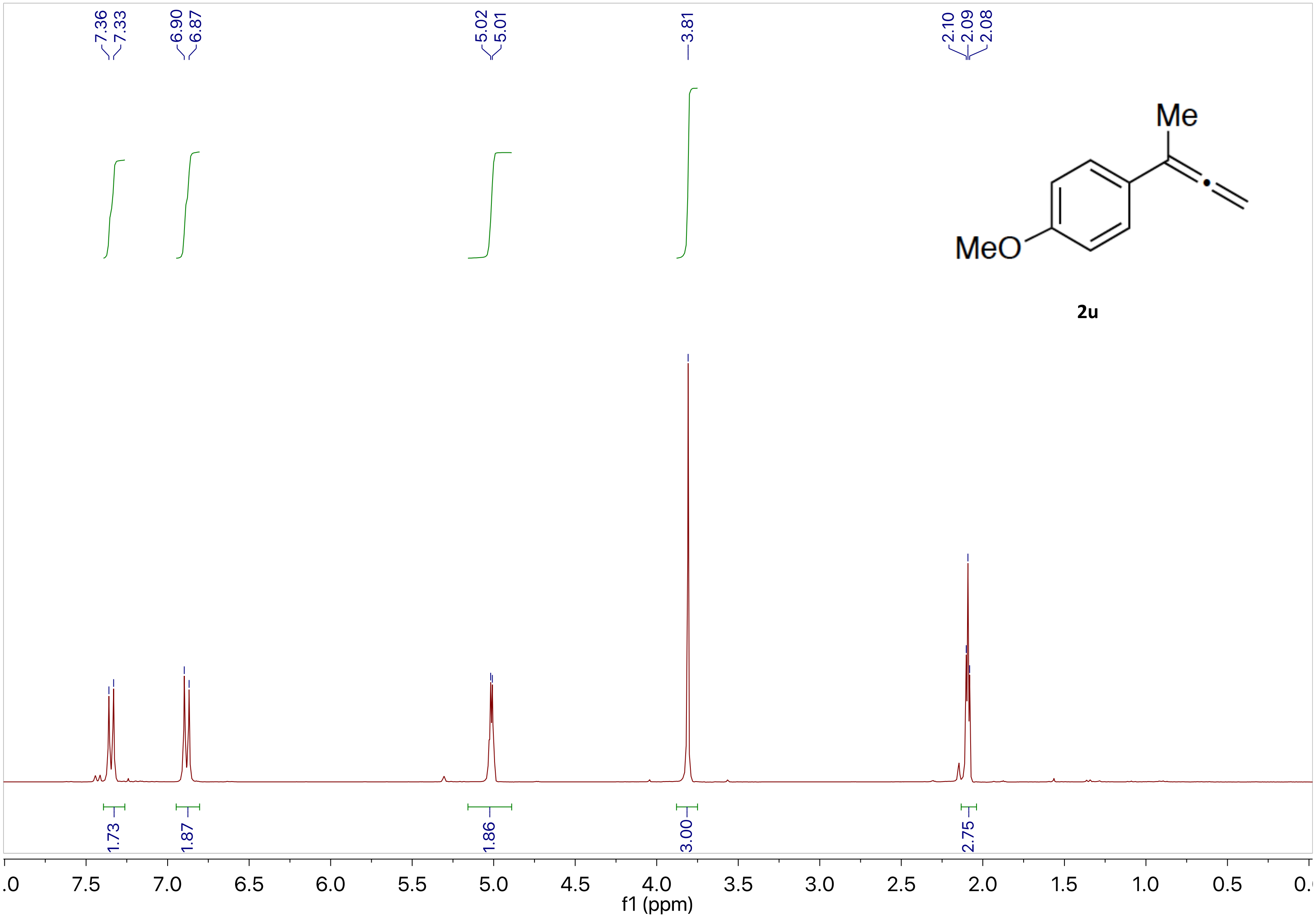
7.36
7.33
6.90
6.87
5.02
5.01
3.81
2.10
2.09
2.08
Me
MeO
1.73
1.87
1.86
3.00
2.75
7.5
7.0
6.5
6.0
5.5
5.0
4.5
4.0
3.5
3.0
2.5
2.0
1.5
1.0
0.5
f1 (ppm)

2u

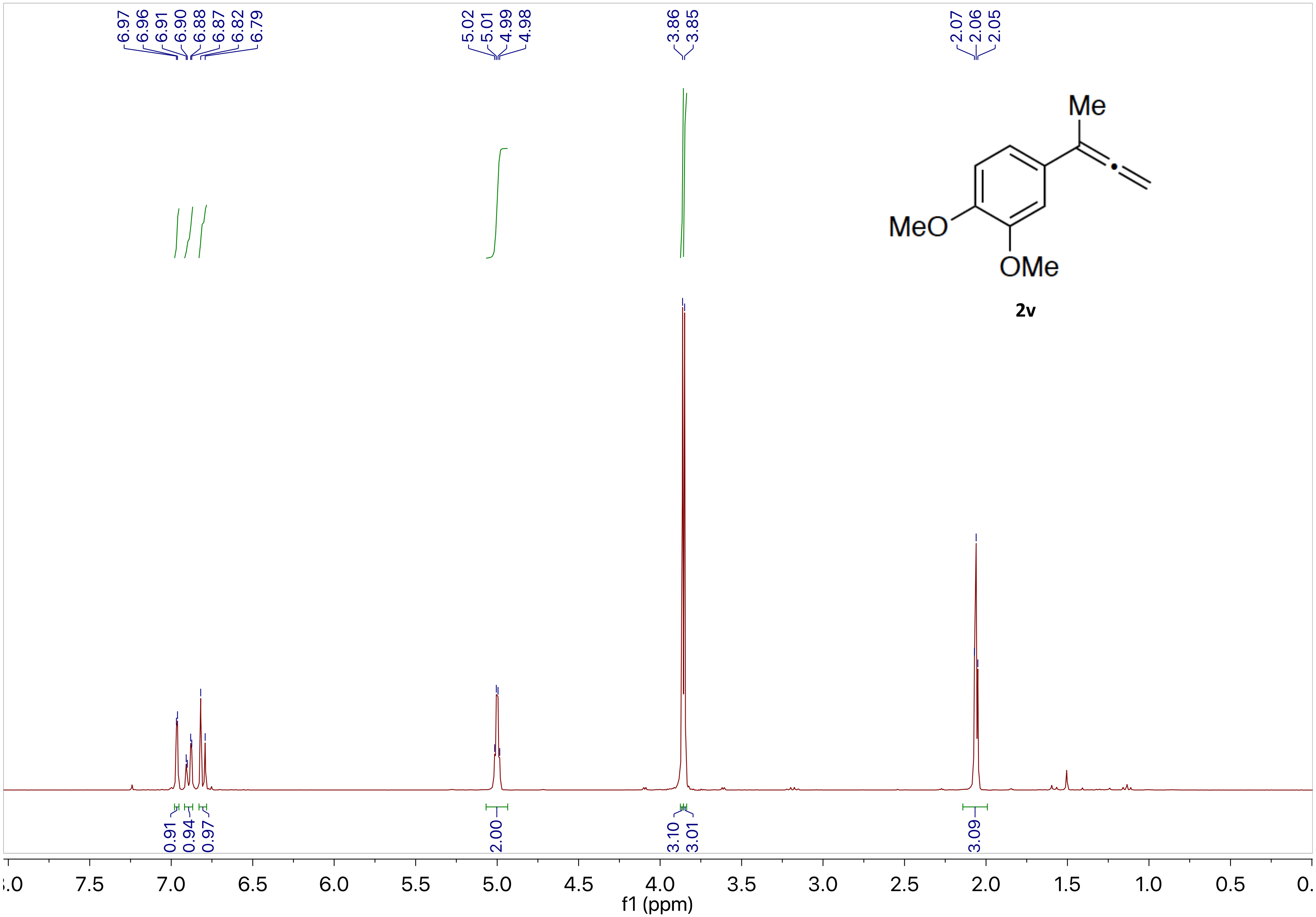

6.97
6.96
6.91
6.90
6.88
6.87
6.82
6.79
5.02
5.01
4.99
4.98
3.86
3.85
2.07
2.06
2.05
Me
MeO
OMe
2v
0.91
0.94
0.97
2.00
3.10
3.01
3.09
f1 (ppm)

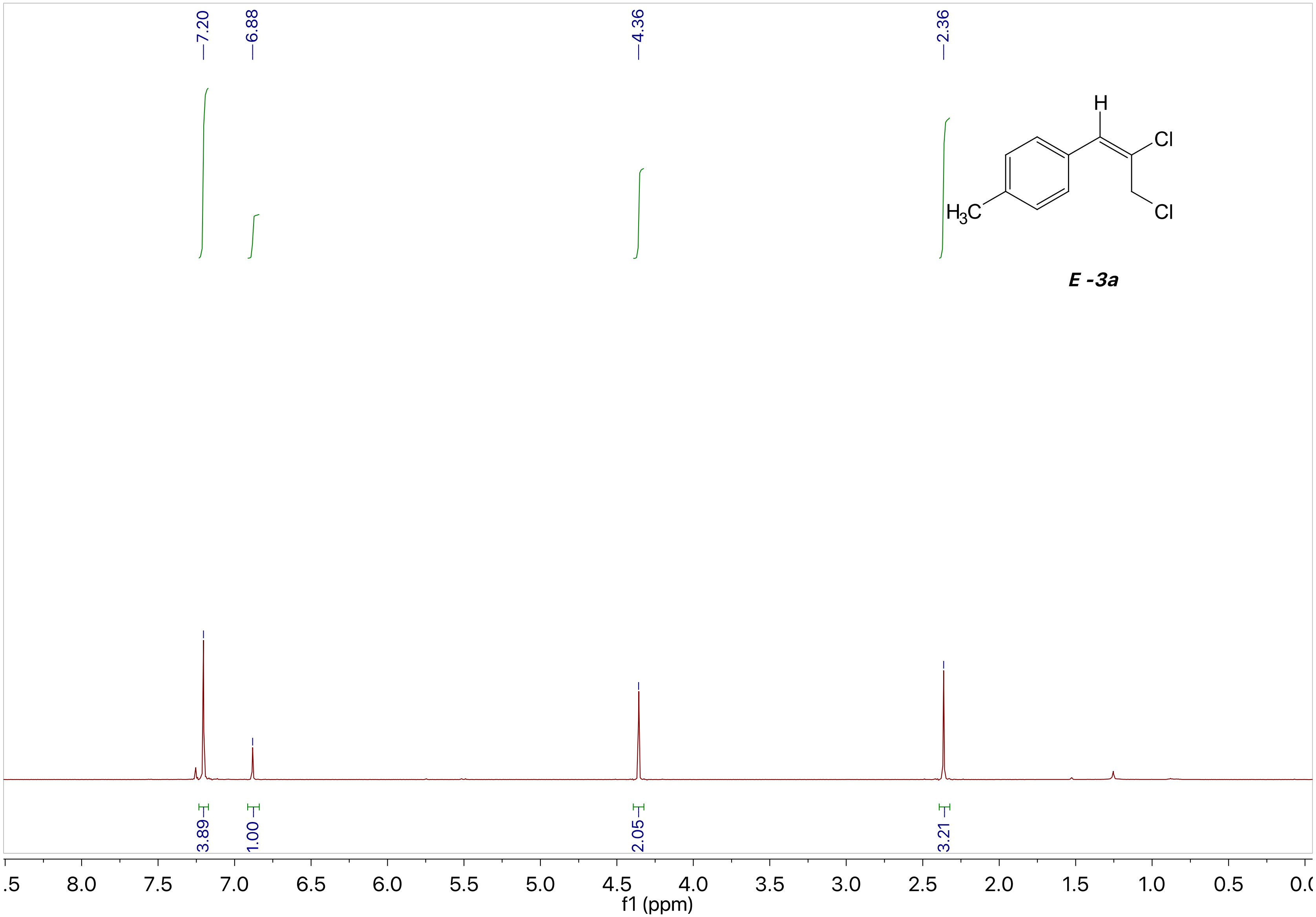
7.20
6.88
4.36
2.36
H
Cl
Cl
H3C
3.89
1.00
2.05
3.21
.5
8.0
7.5
7.0
6.5
6.0
5.5
5.0
4.5
4.0
3.5
3.0
2.5
2.0
1.5
1.0
0.5
f1 (ppm)

***E*-3a**

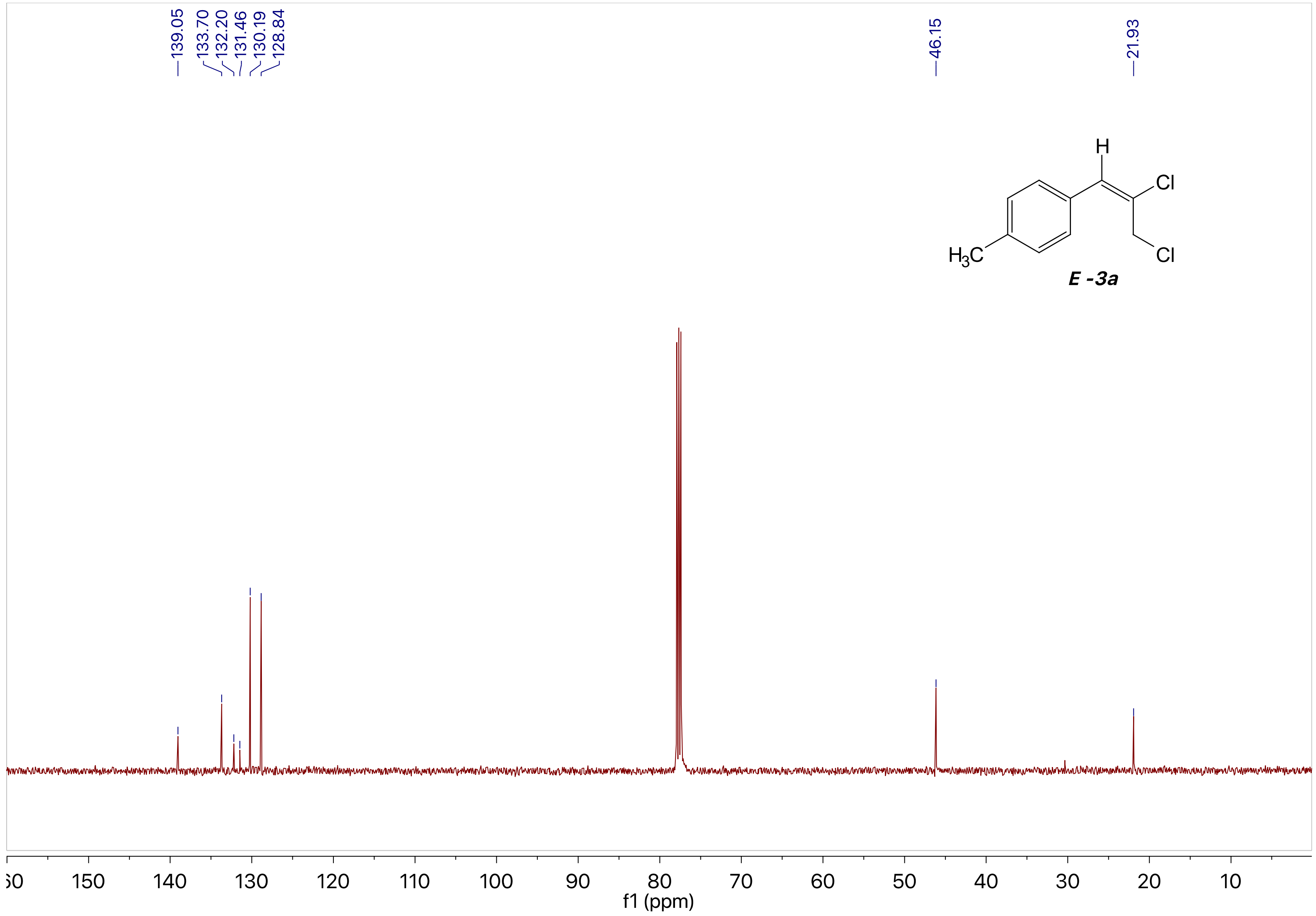
139.05
133.70
132.20
131.46
130.19
128.84
46.15
21.93
H
Cl
Cl
H_3C
E -3a
f1 (ppm)

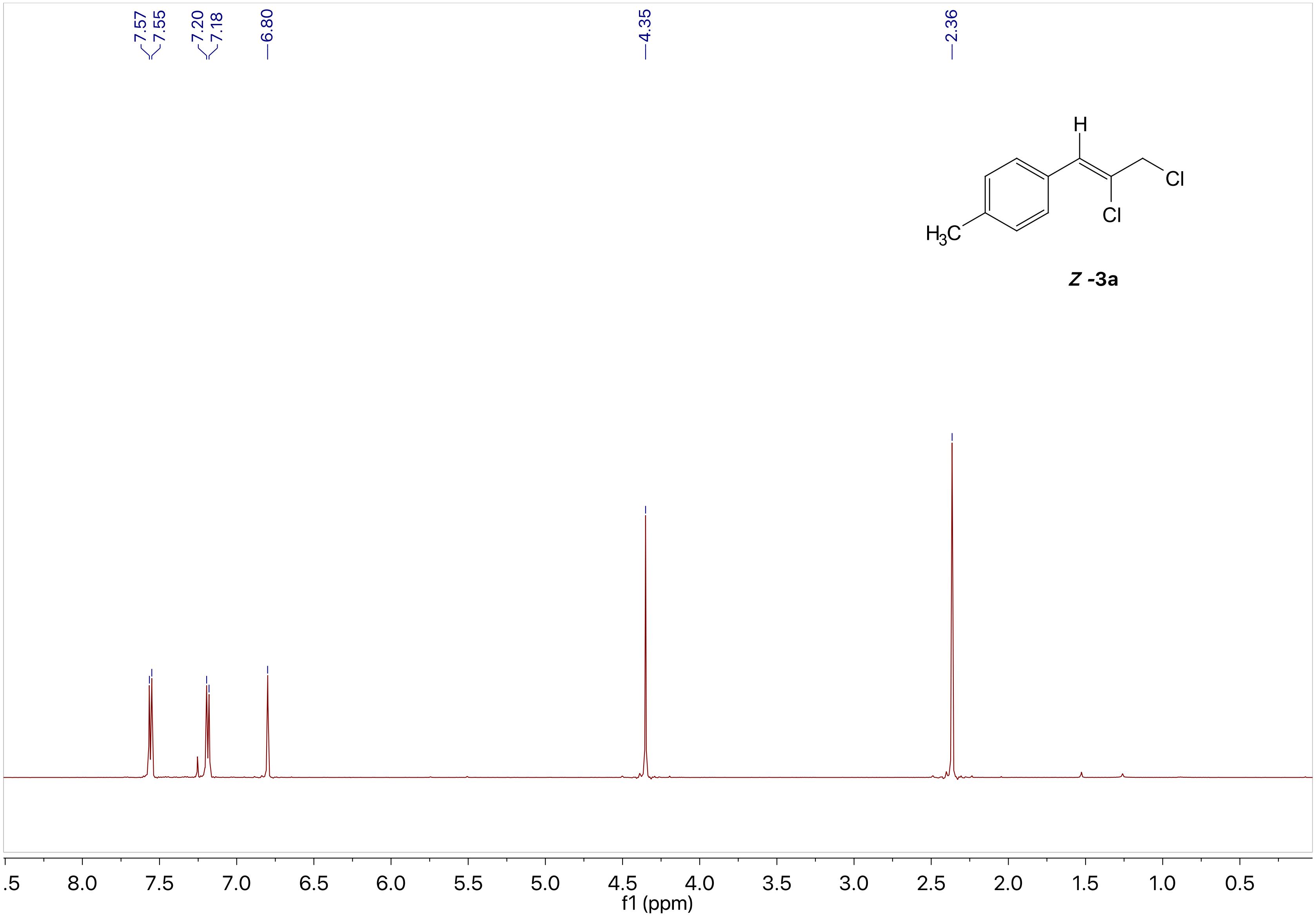

7.57
7.55
7.20
7.18
6.80
4.35
2.36
H
Cl
Cl
H3C
Z -3a
.5
8.0
7.5
7.0
6.5
6.0
5.5
5.0
4.5
4.0
3.5
3.0
2.5
2.0
1.5
1.0
0.5
f1 (ppm)

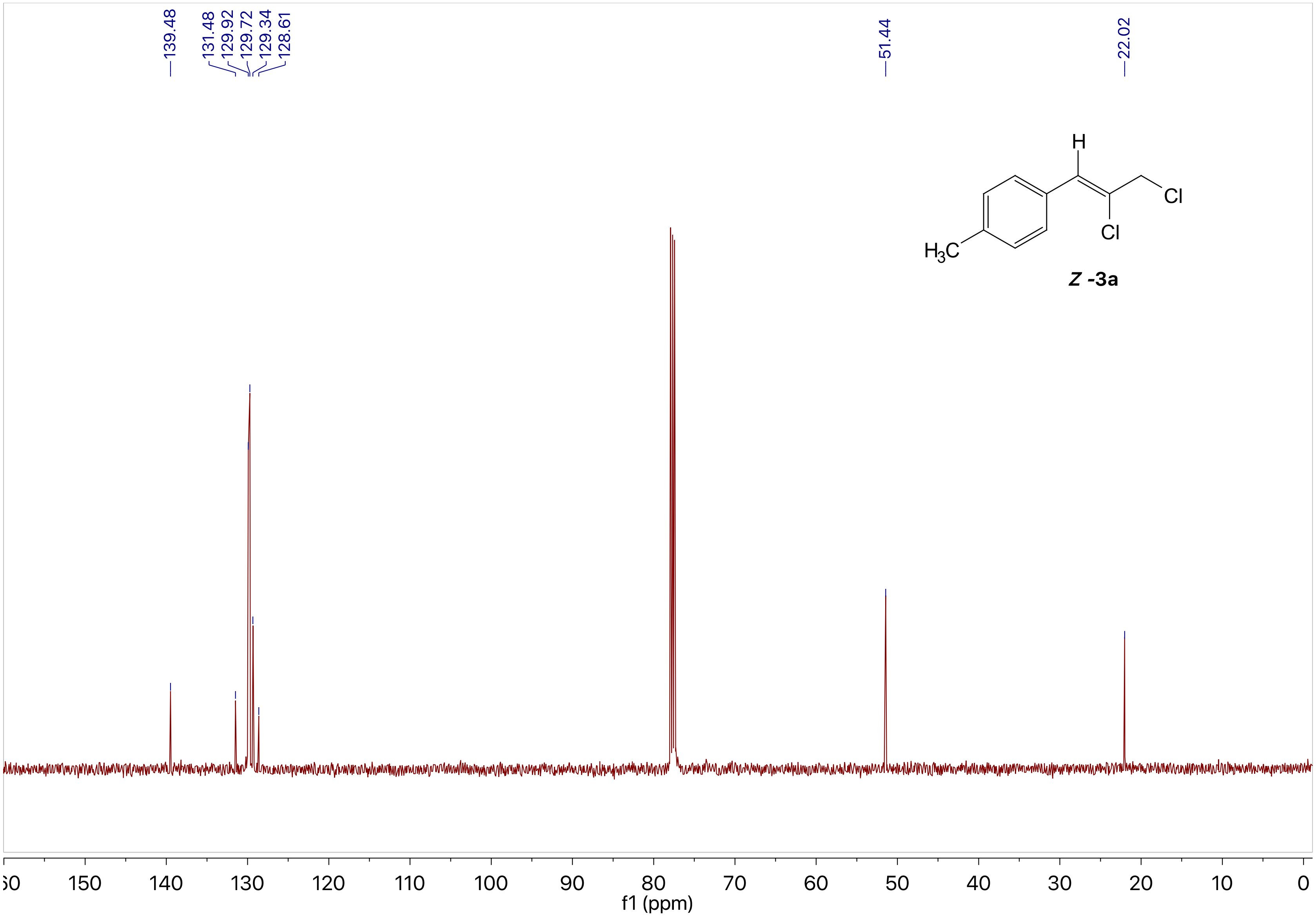
139.48
131.48
129.92
129.72
129.34
128.61
51.44
22.02
H
Cl
Cl
H3C
Z -3a
150
140
130
120
110
100
90
80
70
60
50
40
30
20
10
0
f1 (ppm)

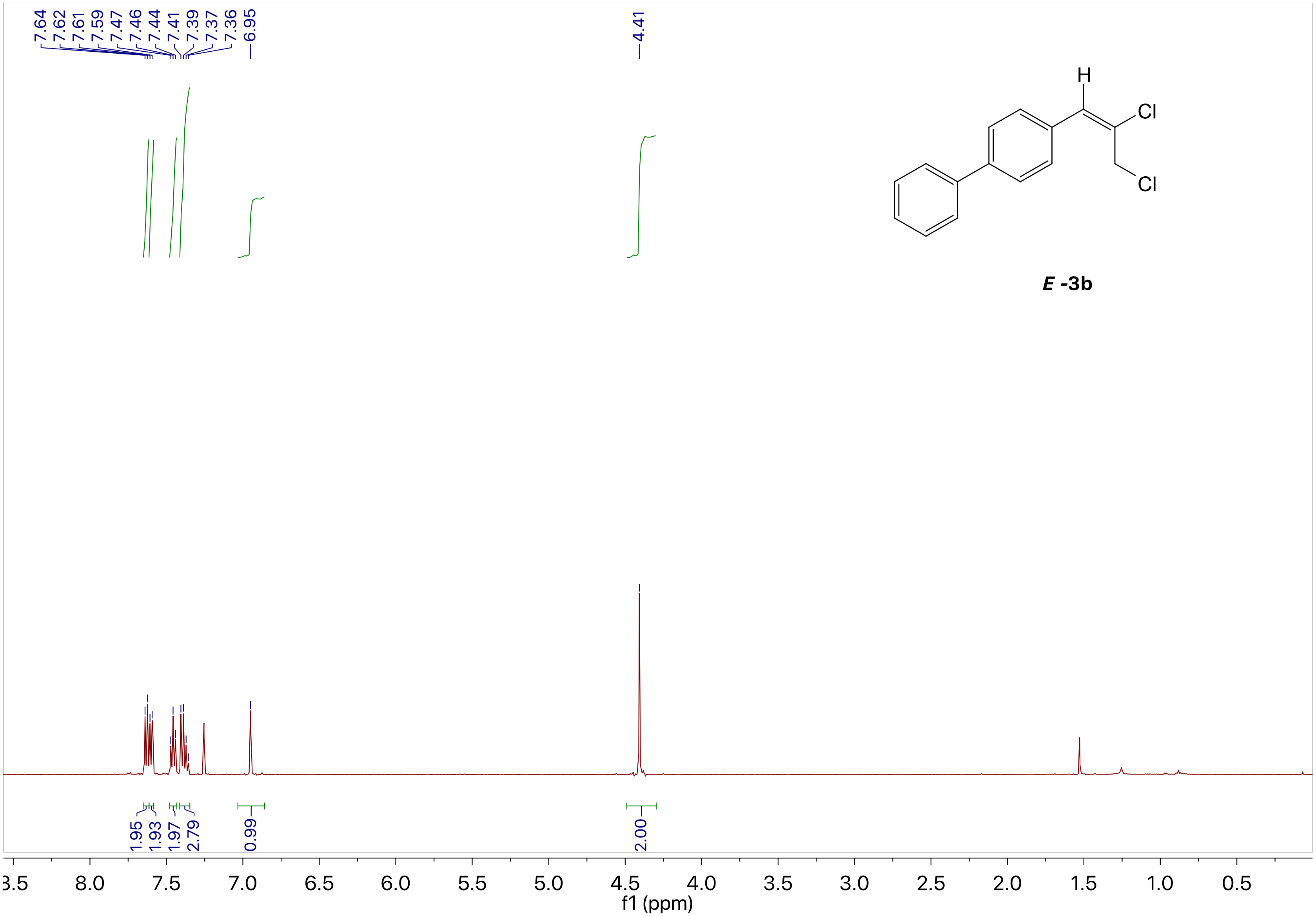
7.64
7.62
7.61
7.59
7.47
7.46
7.44
7.41
7.39
7.37
7.36
6.95
4.41
1.95
1.93
1.97
2.79
0.99
2.00
8.5
8.0
7.5
7.0
6.5
6.0
5.5
5.0
4.5
4.0
3.5
3.0
2.5
2.0
1.5
1.0
0.5
f1 (ppm)
H
Cl
Cl

***E* -3b**

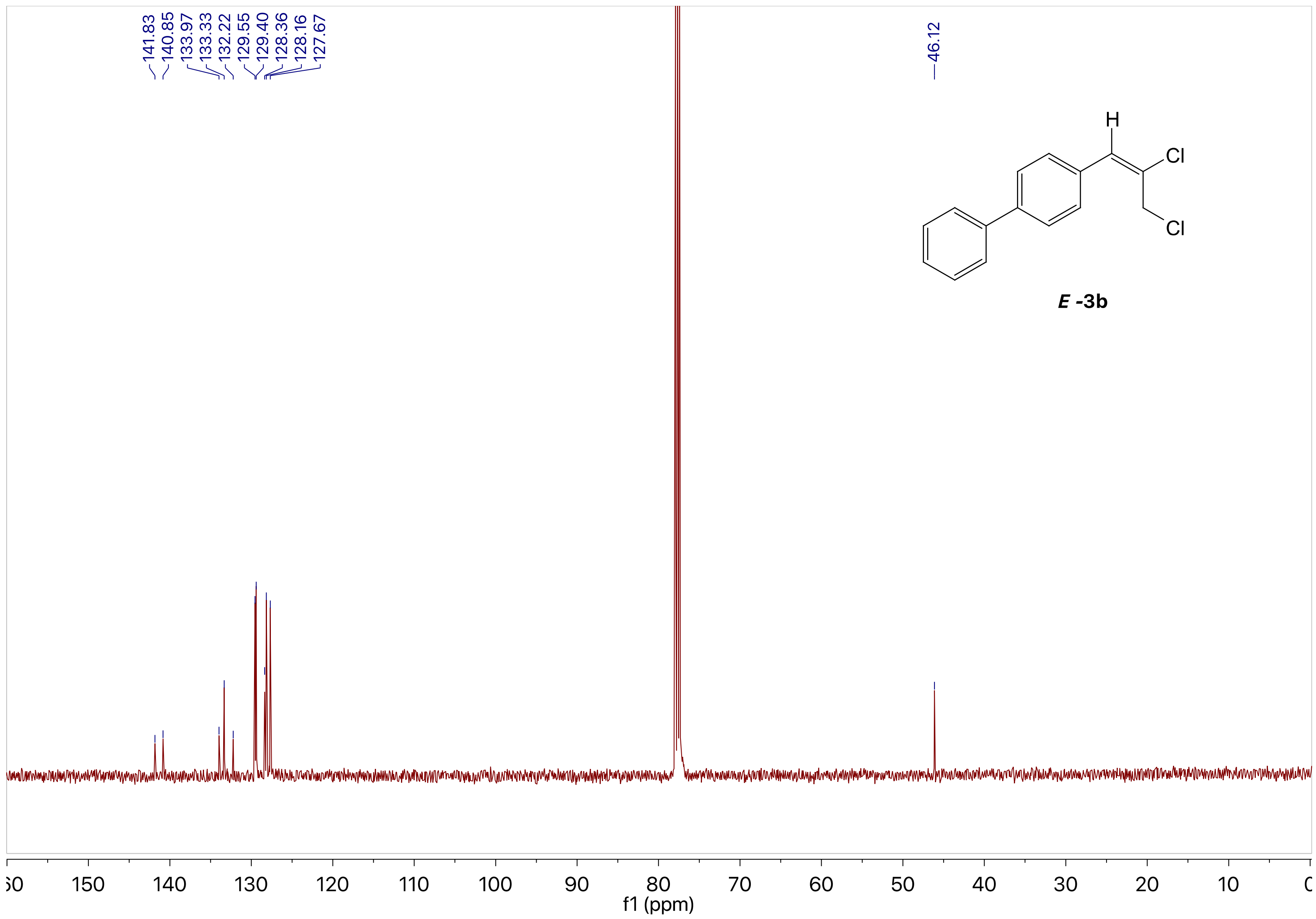
141.83
140.85
133.97
133.33
132.22
129.55
129.40
128.36
128.16
127.67
46.12
H
Cl
Cl
E -3b
150
140
130
120
110
100
90
80
70
60
50
40
30
20
10
f1 (ppm)

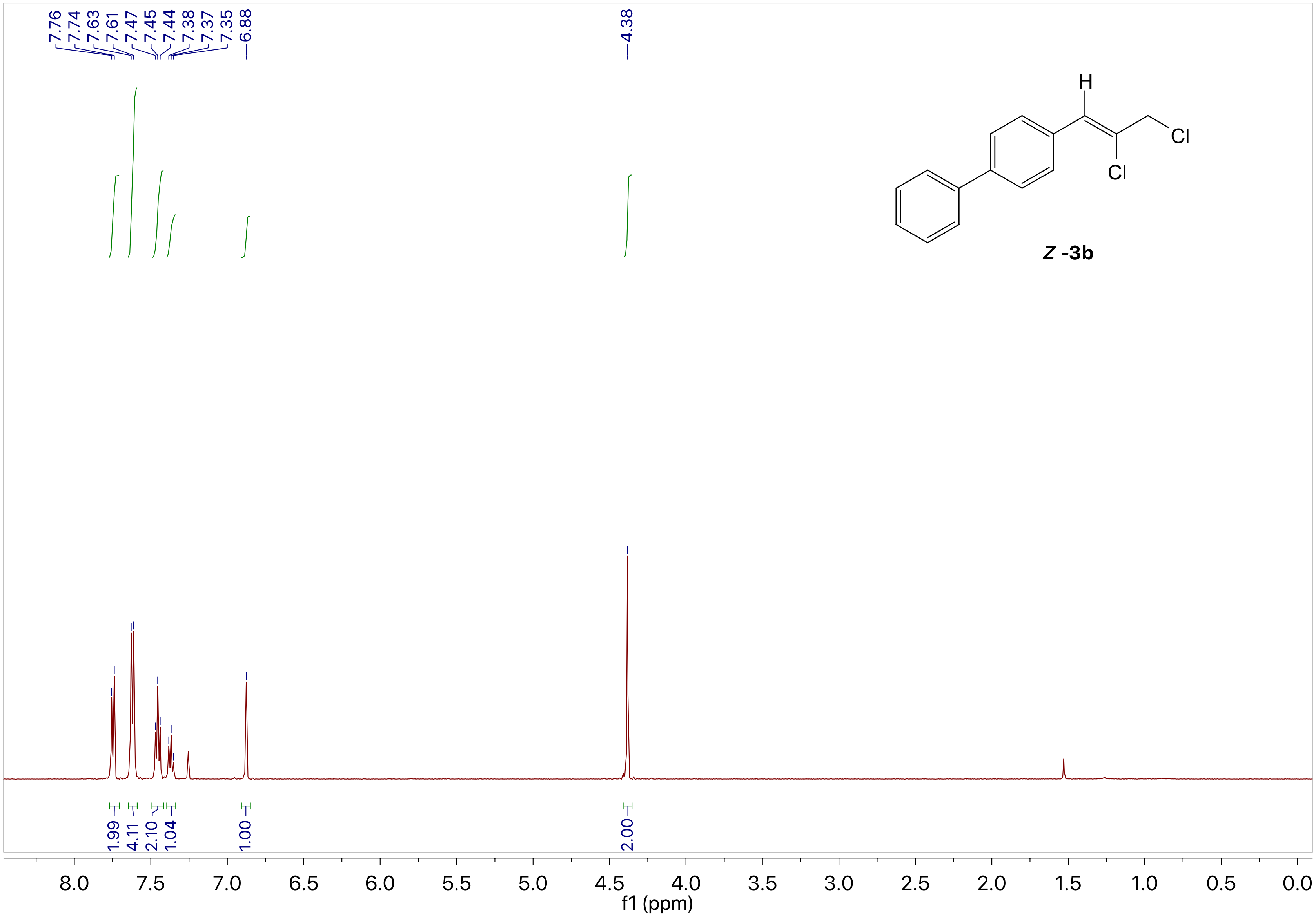
7.76
7.74
7.63
7.61
7.47
7.45
7.44
7.38
7.37
7.35
6.88
4.38
H
Cl
Cl
Z -3b
1.99
4.11
2.10
1.04
1.00
2.00
8.0
7.5
7.0
6.5
6.0
5.5
5.0
4.5
4.0
3.5
3.0
2.5
2.0
1.5
1.0
0.5
0.0
f1 (ppm)

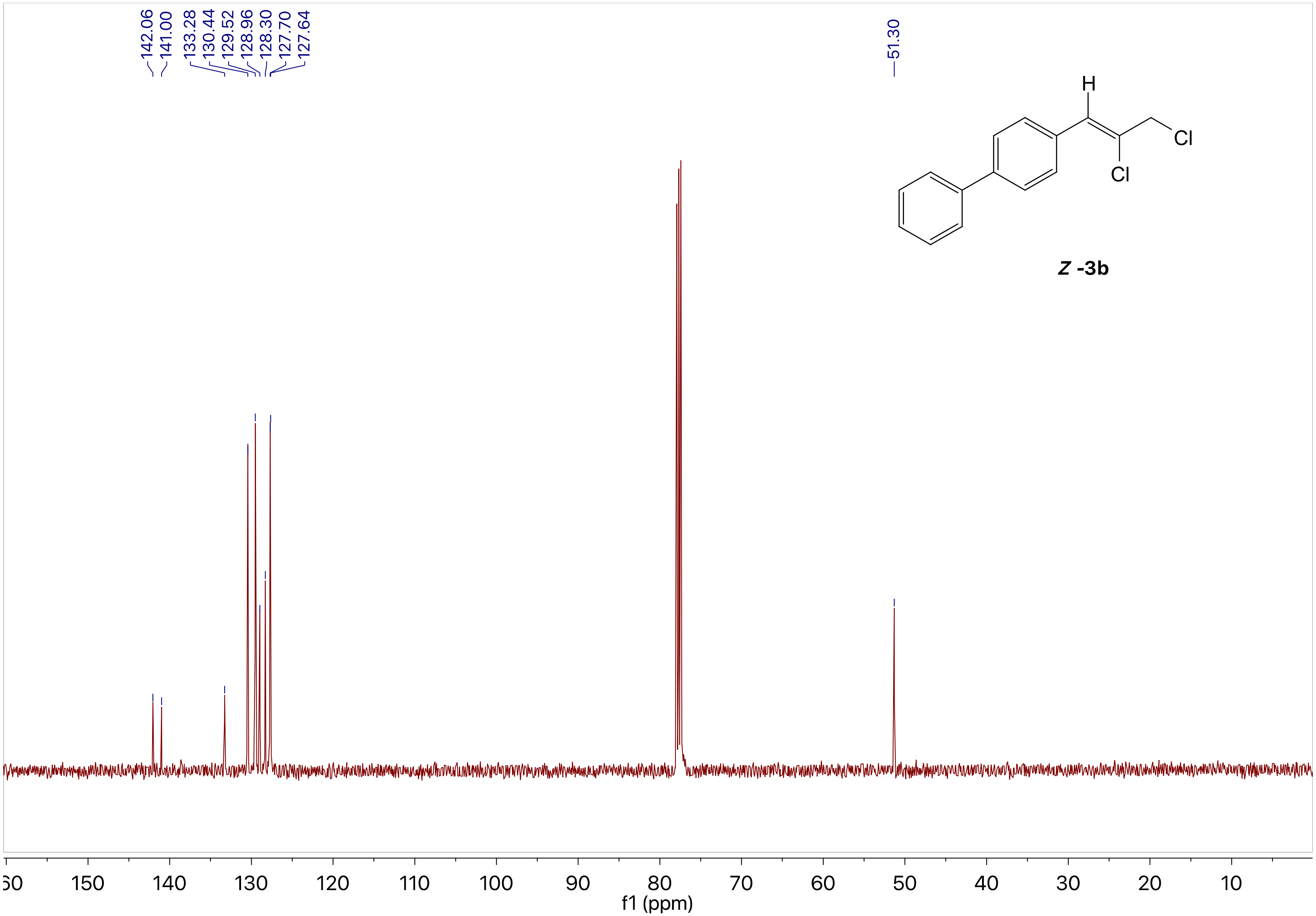
142.06
141.00
133.28
130.44
129.52
128.96
128.30
127.70
127.64
51.30
H
Cl
Cl
Z -3b
150
140
130
120
110
100
90
80
70
60
50
40
30
20
10
f1 (ppm)

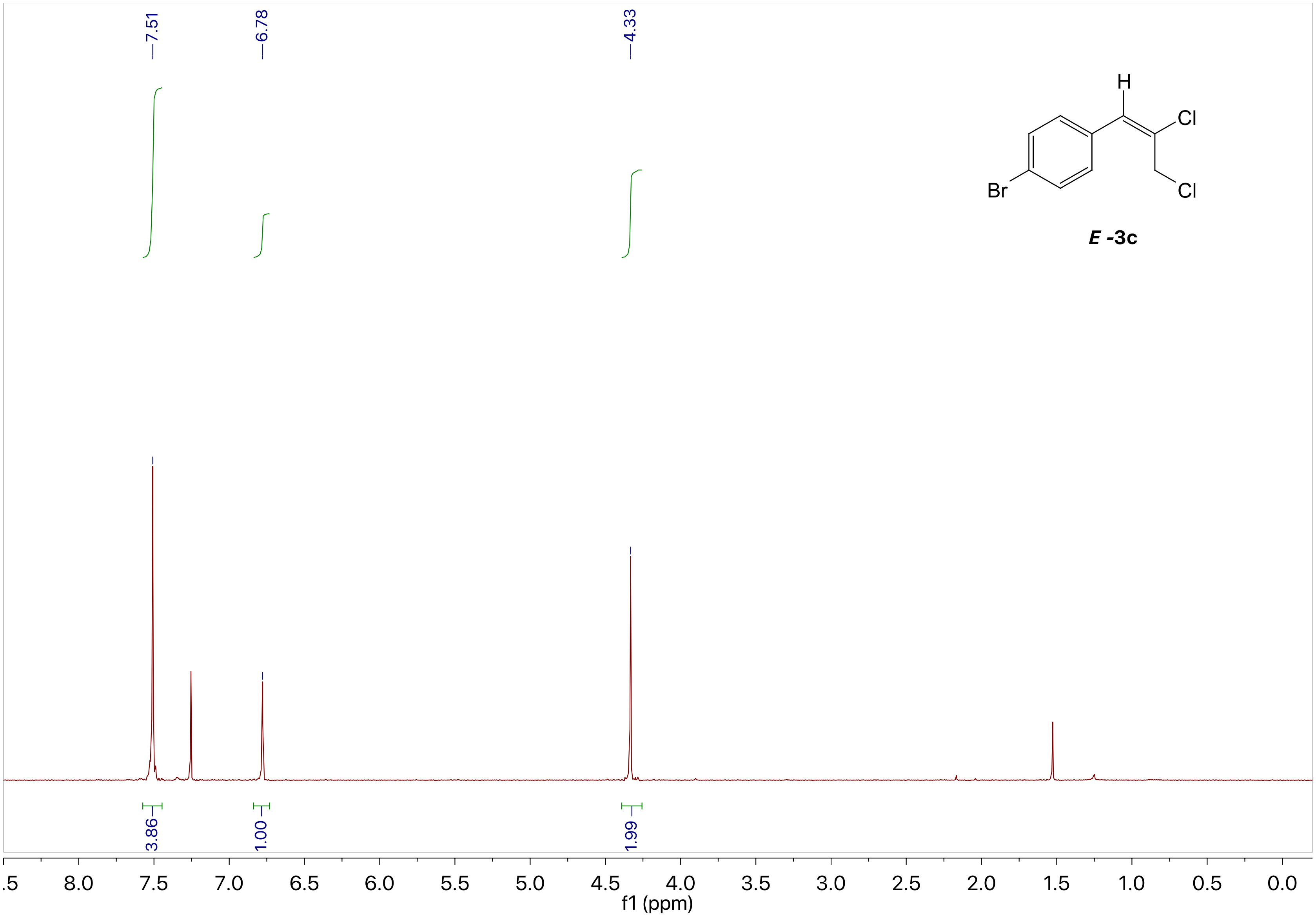
7.51
6.78
4.33
H
Cl
Br
Cl
3.86
1.00
1.99
.5
8.0
7.5
7.0
6.5
6.0
5.5
5.0
4.5
4.0
3.5
3.0
2.5
2.0
1.5
1.0
0.5
0.0
f1 (ppm)

***E*-3c**

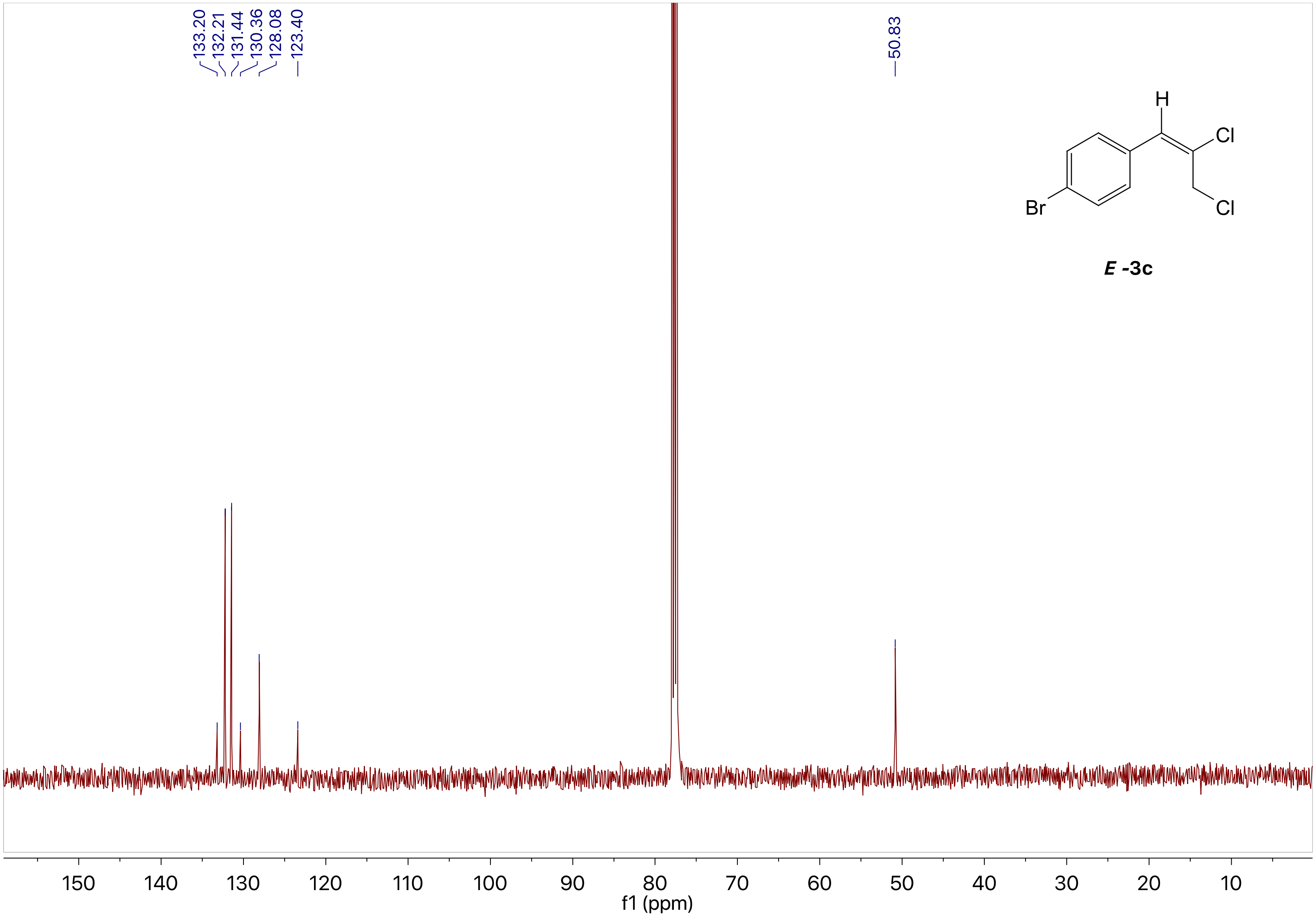
133.20
132.21
131.44
130.36
128.08
123.40
50.83
H
Cl
Br
Cl
E -3c
150
140
130
120
110
100
90
80
70
60
50
40
30
20
10
f1 (ppm)

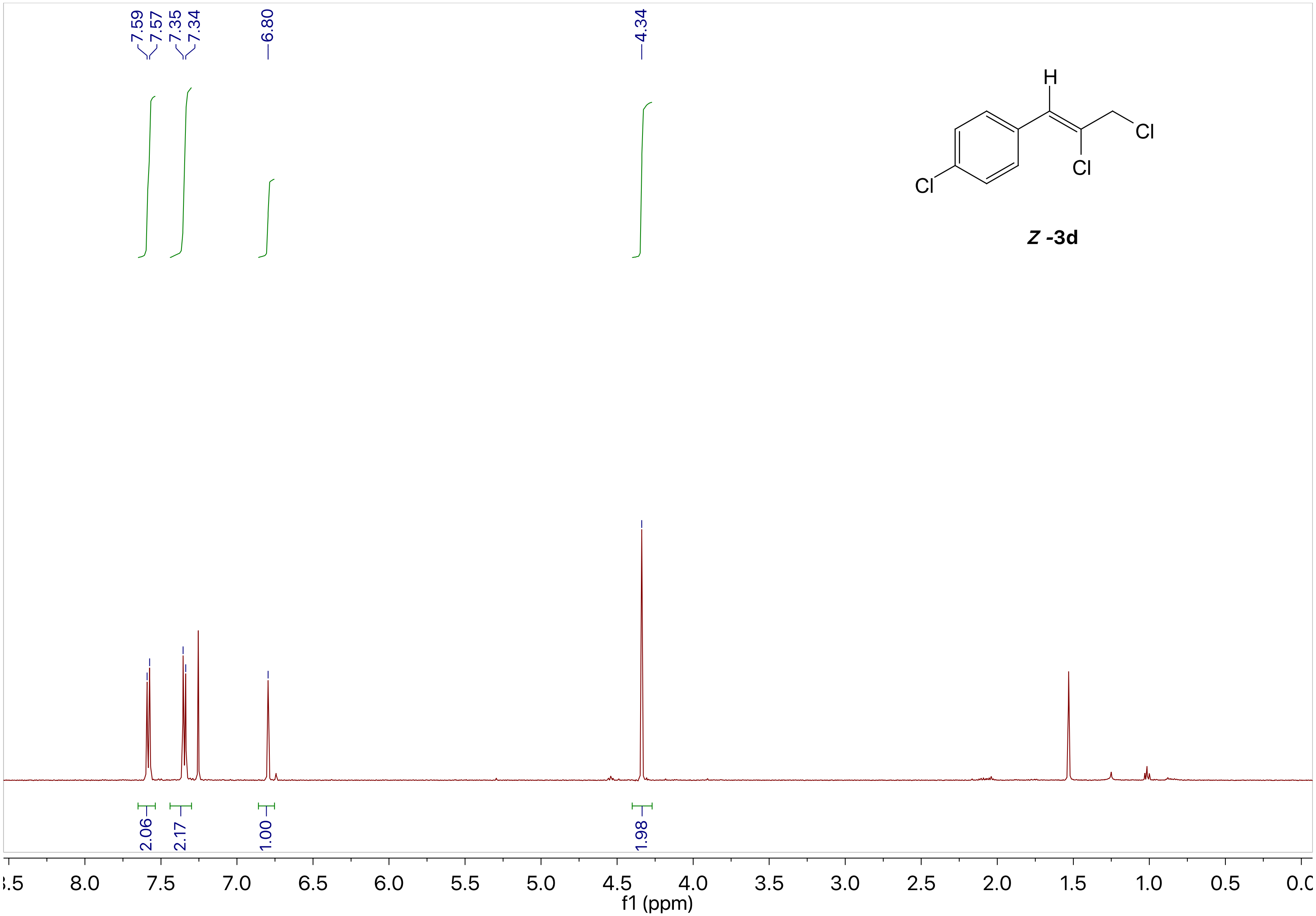

7.59
7.57
7.35
7.34
6.80
4.34
H
Cl
Cl
Cl
2.06
2.17
1.00
1.98
8.0
7.5
7.0
6.5
6.0
5.5
5.0
4.5
4.0
3.5
3.0
2.5
2.0
1.5
1.0
0.5
f1 (ppm)

***Z*-3d**

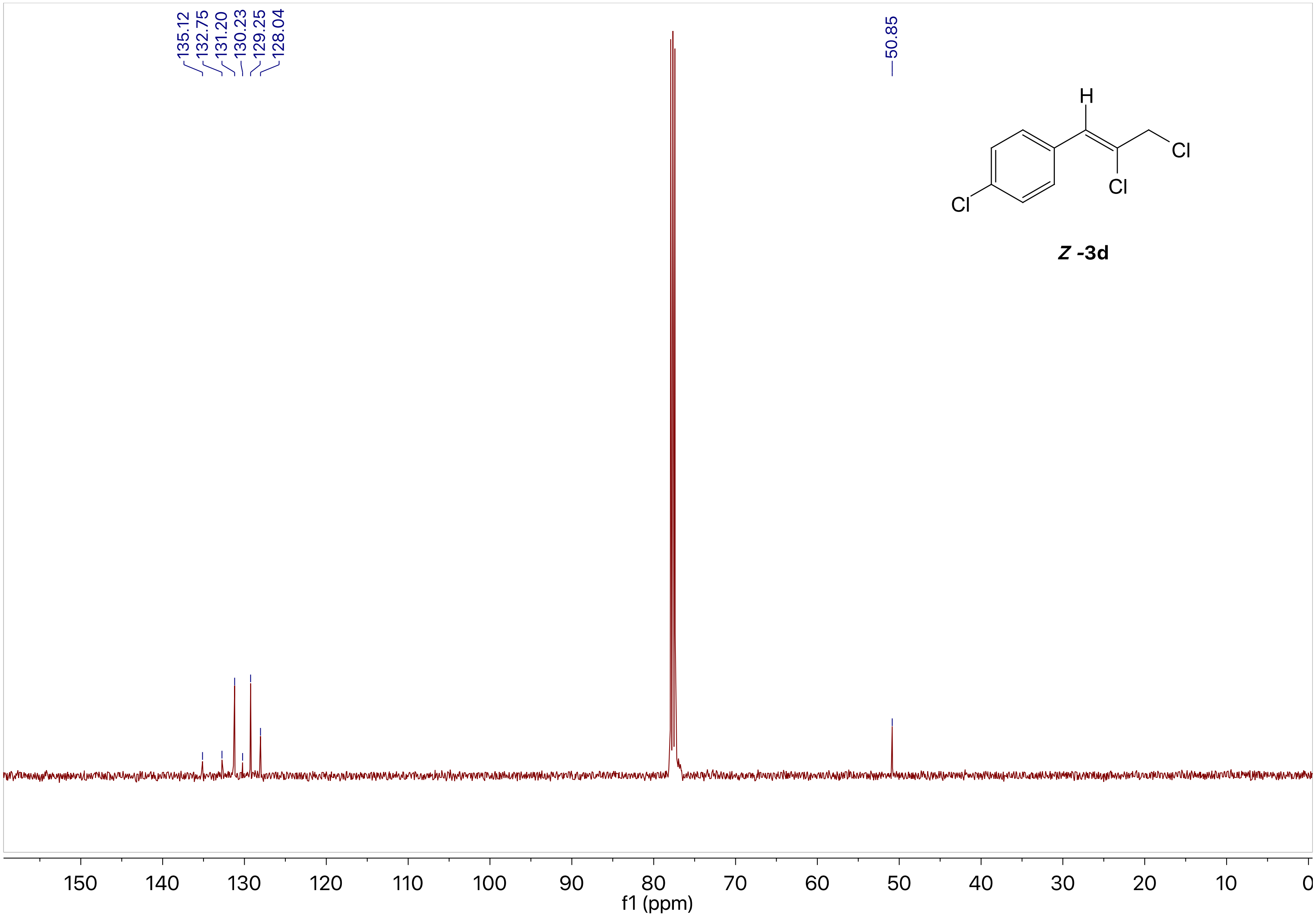
135.12
132.75
131.20
130.23
129.25
128.04
50.85
H
Cl
Cl
Cl
Z -3d
150
140
130
120
110
100
90
80
70
60
50
40
30
20
10
0
f1 (ppm)

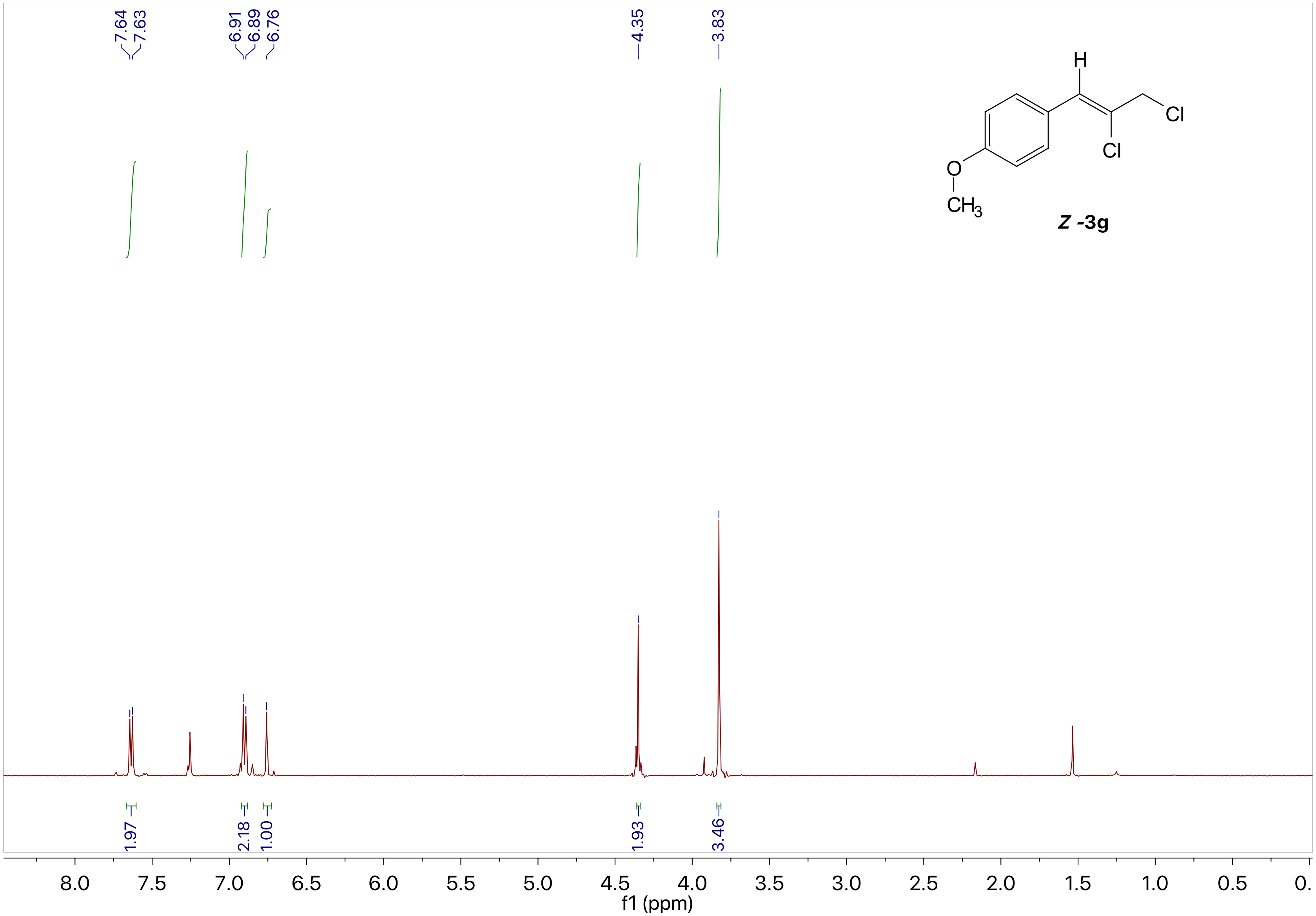
7.64
7.63
6.91
6.89
6.76
4.35
3.83
H
Cl
Cl
O
CH3
Z -3g
1.97
2.18
1.00
1.93
3.46
8.0
7.5
7.0
6.5
6.0
5.5
5.0
4.5
4.0
3.5
3.0
2.5
2.0
1.5
1.0
0.5
0.
f1 (ppm)

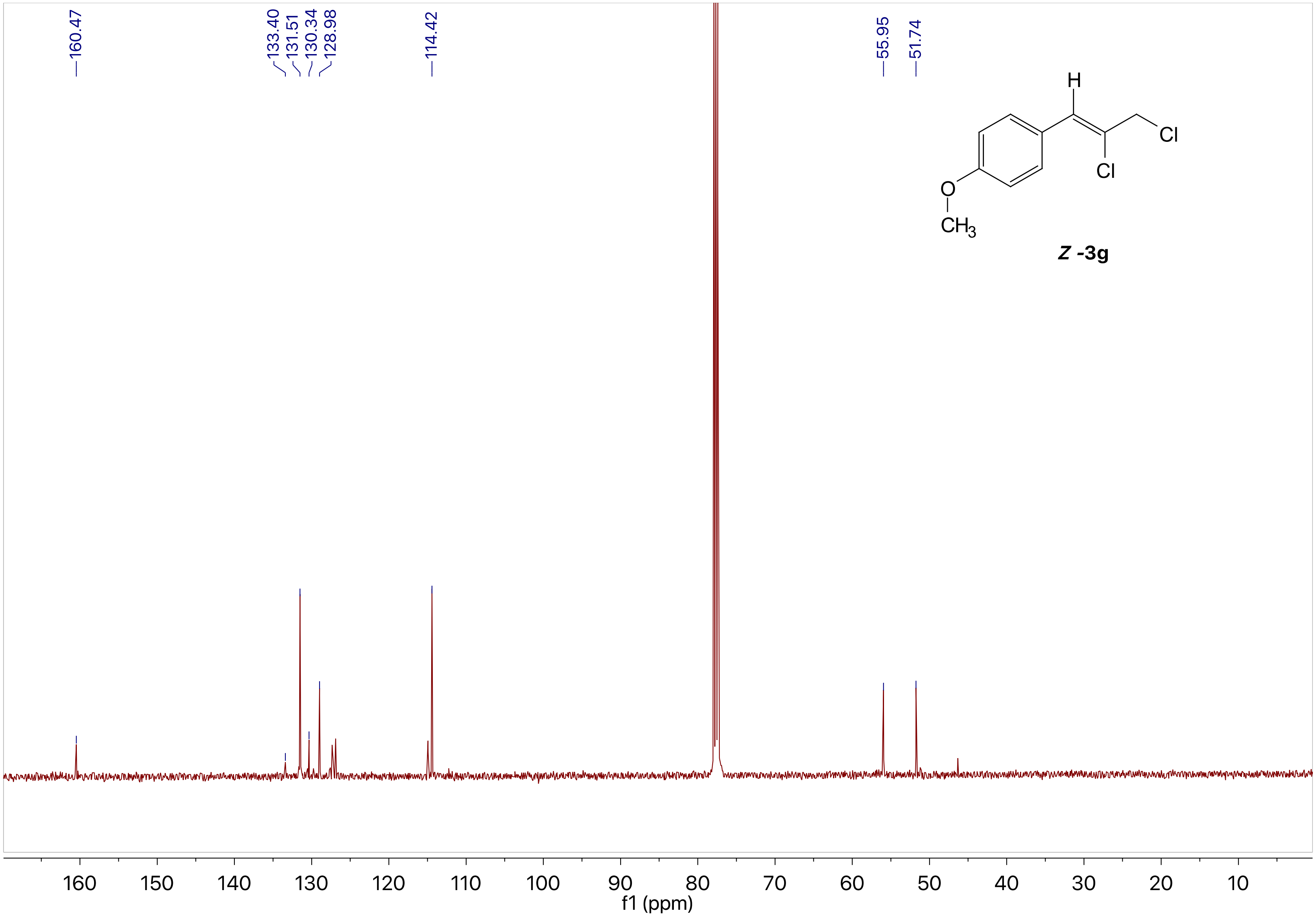
160.47
133.40
131.51
130.34
128.98
114.42
55.95
51.74
H
Cl
Cl
O
CH3
Z -3g
160
150
140
130
120
110
100
90
80
70
60
50
40
30
20
10
f1 (ppm)

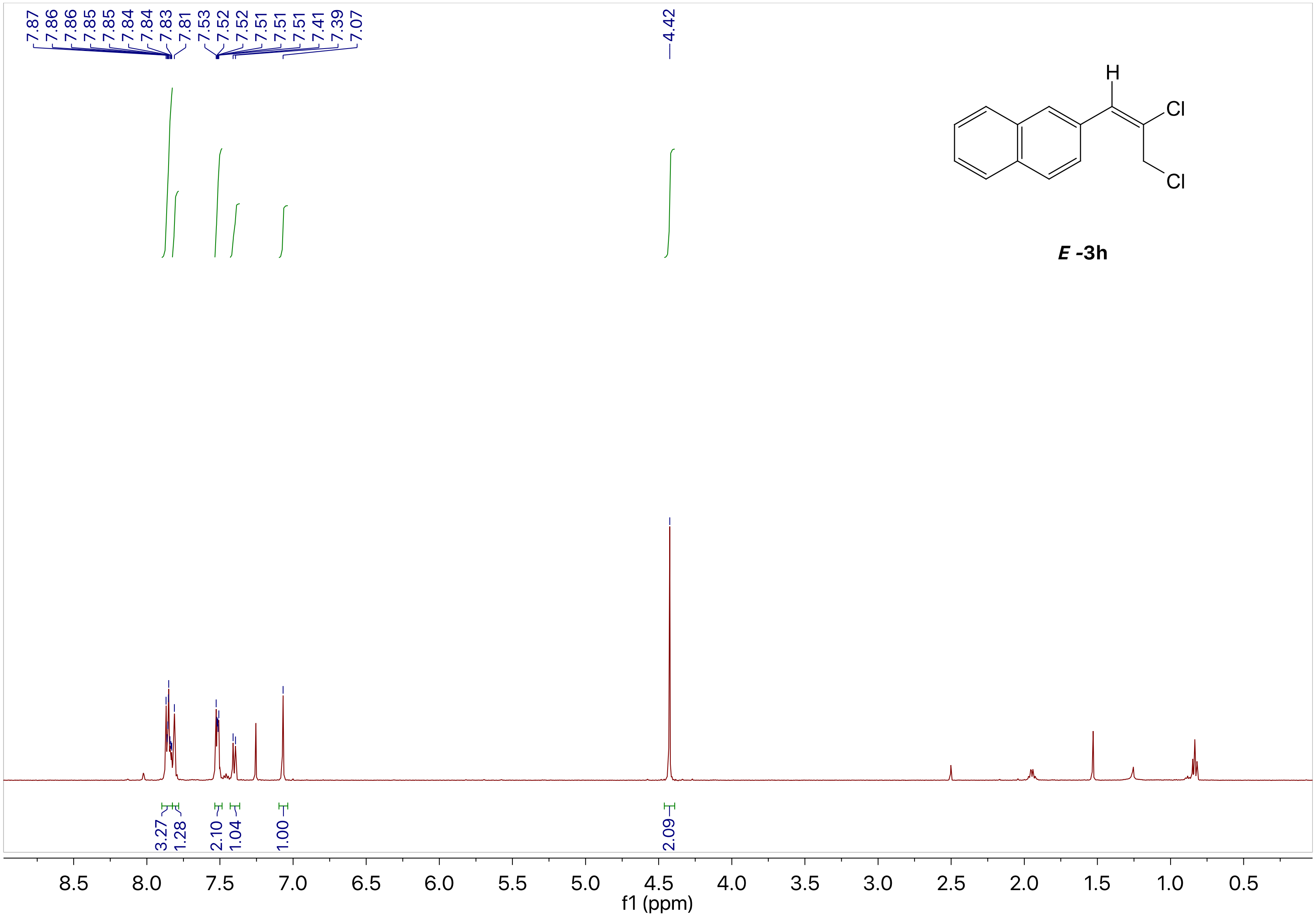
7.87
7.86
7.86
7.85
7.85
7.84
7.84
7.83
7.81
7.53
7.52
7.52
7.51
7.51
7.51
7.41
7.39
7.07
4.42
H
Cl
Cl
3.27
1.28
2.10
1.04
1.00
2.09
8.5
8.0
7.5
7.0
6.5
6.0
5.5
5.0
4.5
4.0
3.5
3.0
2.5
2.0
1.5
1.0
0.5
f1 (ppm)

***E*-3h**

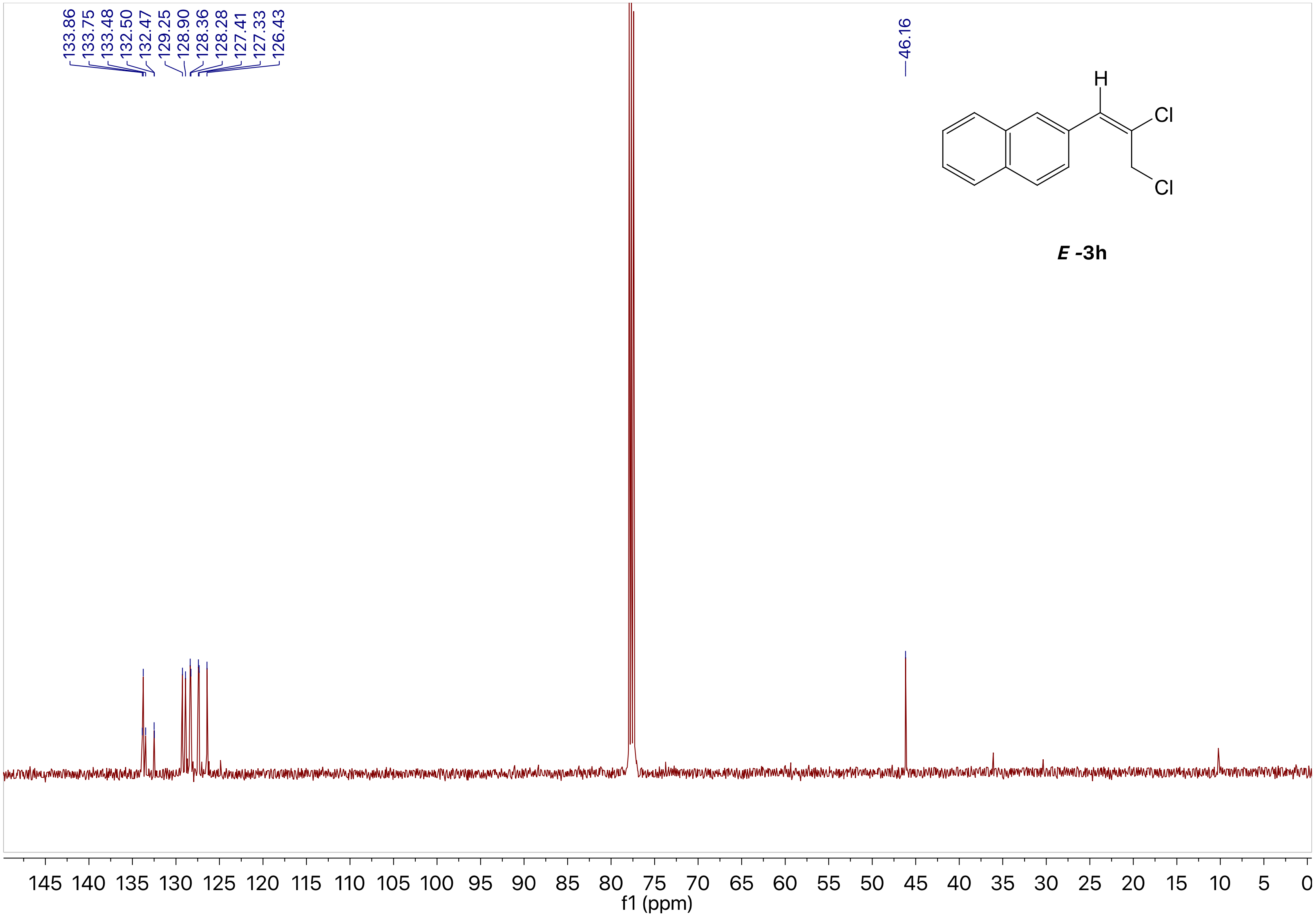
133.86
133.75
133.48
132.50
132.47
129.25
128.90
128.36
128.28
127.41
127.33
126.43
46.16
H
Cl
Cl
E -3h
145 140 135 130 125 120 115 110 105 100 95 90 85 80 75 70 65 60 55 50 45 40 35 30 25 20 15 10 5 0
f1 (ppm)

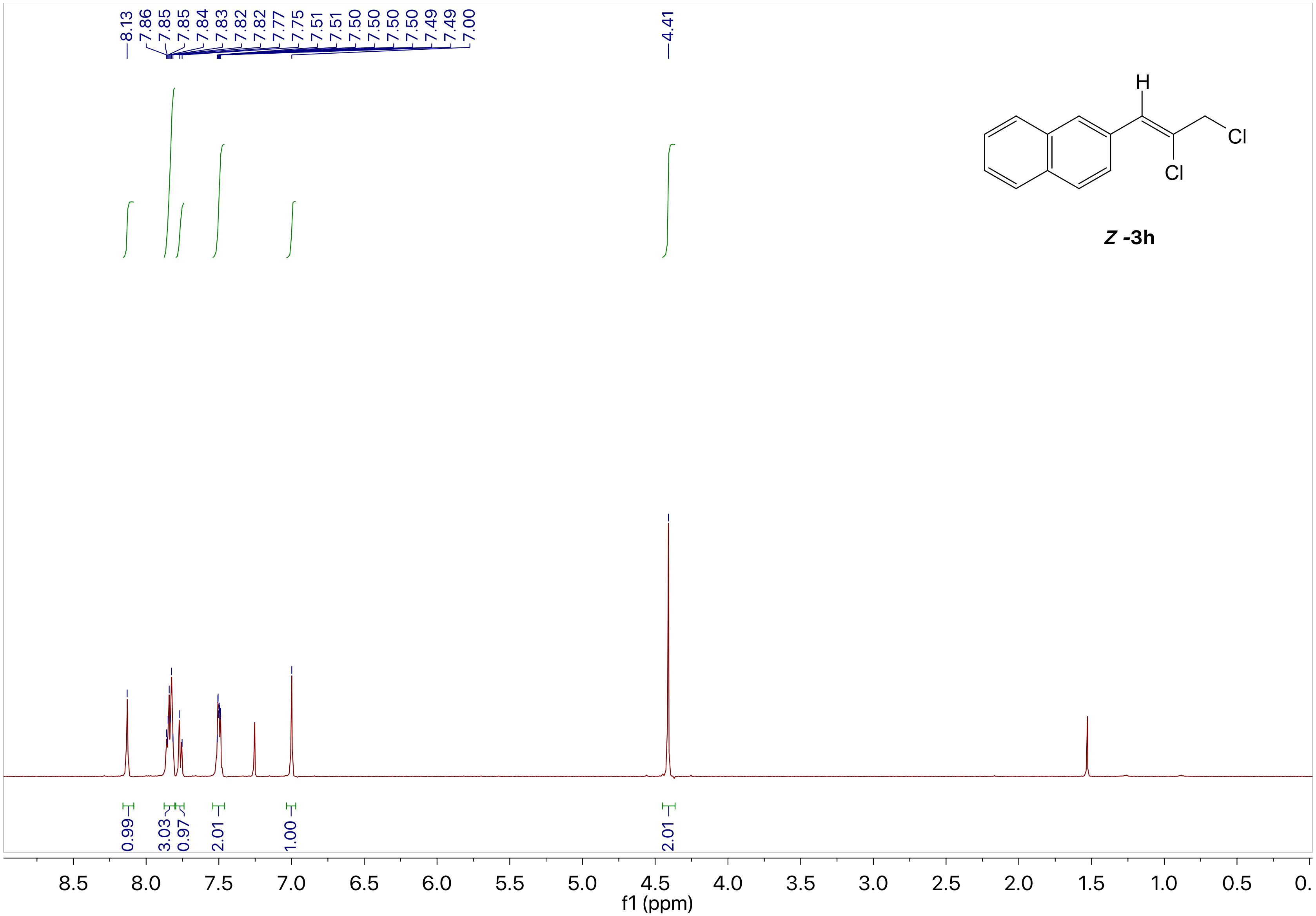

8.13
7.86
7.85
7.85
7.84
7.83
7.82
7.82
7.77
7.75
7.51
7.51
7.50
7.50
7.50
7.50
7.49
7.49
7.00
4.41
H
Cl
Cl
Z -3h
0.99
3.03
0.97
2.01
1.00
2.01
8.5
8.0
7.5
7.0
6.5
6.0
5.5
5.0
4.5
4.0
3.5
3.0
2.5
2.0
1.5
1.0
0.5
0.
f1 (ppm)

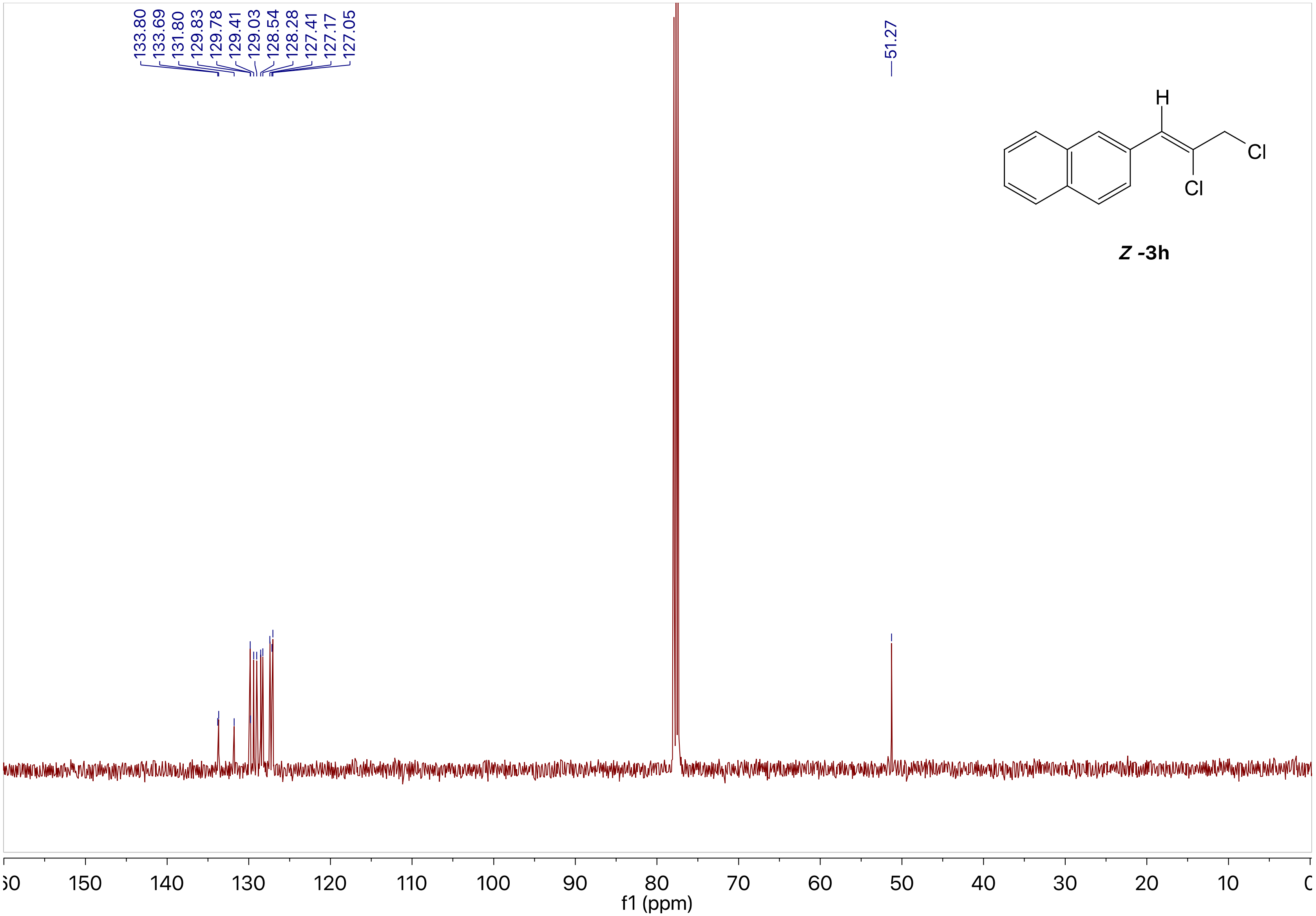
133.80
133.69
131.80
129.83
129.78
129.41
129.03
128.54
128.28
127.41
127.17
127.05
51.27
H
Cl
Cl
150
140
130
120
110
100
90
80
70
60
50
40
30
20
10
f1 (ppm)

***Z*-3h**

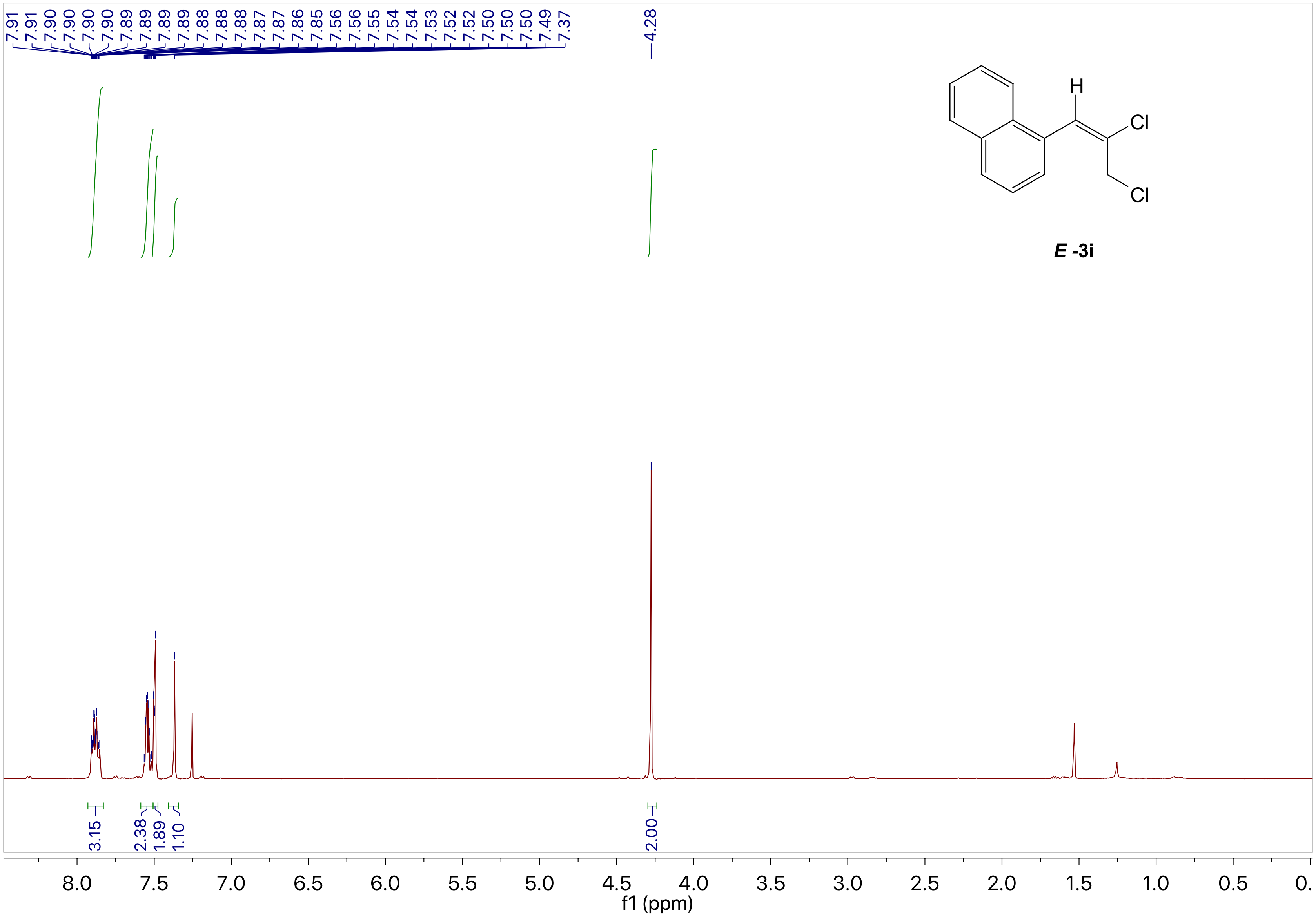

7.91
7.91
7.90
7.90
7.90
7.90
7.89
7.89
7.89
7.89
7.88
7.88
7.88
7.87
7.87
7.86
7.85
7.56
7.56
7.55
7.54
7.54
7.53
7.52
7.52
7.50
7.50
7.50
7.49
7.37
4.28
H
Cl
Cl
3.15
2.38
1.89
1.10
2.00
8.0
7.5
7.0
6.5
6.0
5.5
5.0
4.5
4.0
3.5
3.0
2.5
2.0
1.5
1.0
0.5
0.
f1 (ppm)

E -3i

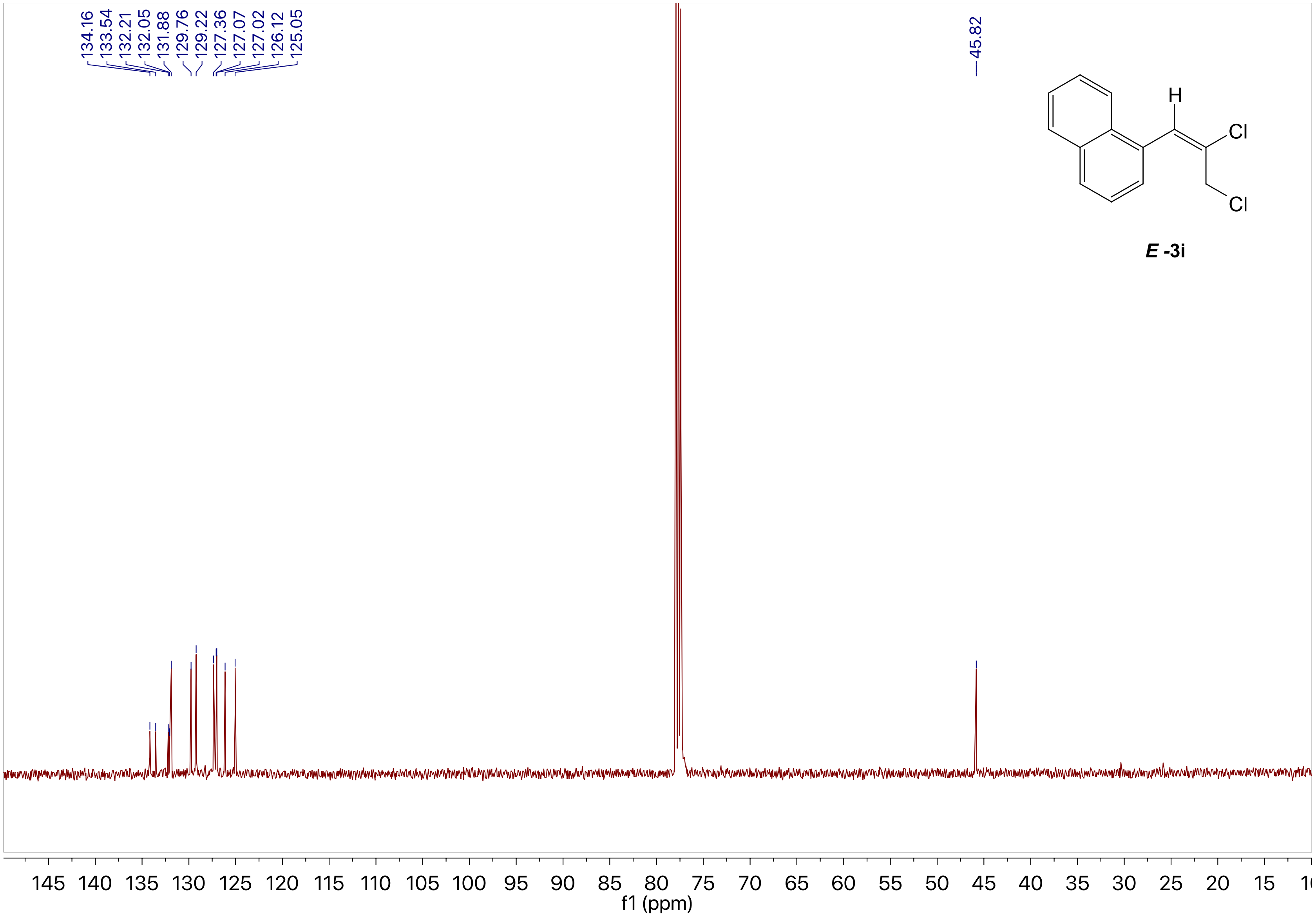
134.16
133.54
132.21
132.05
131.88
129.76
129.22
127.36
127.07
127.02
126.12
125.05
45.82
H
Cl
Cl
E -3i
145 140 135 130 125 120 115 110 105 100 95 90 85 80 75 70 65 60 55 50 45 40 35 30 25 20 15
f1 (ppm)

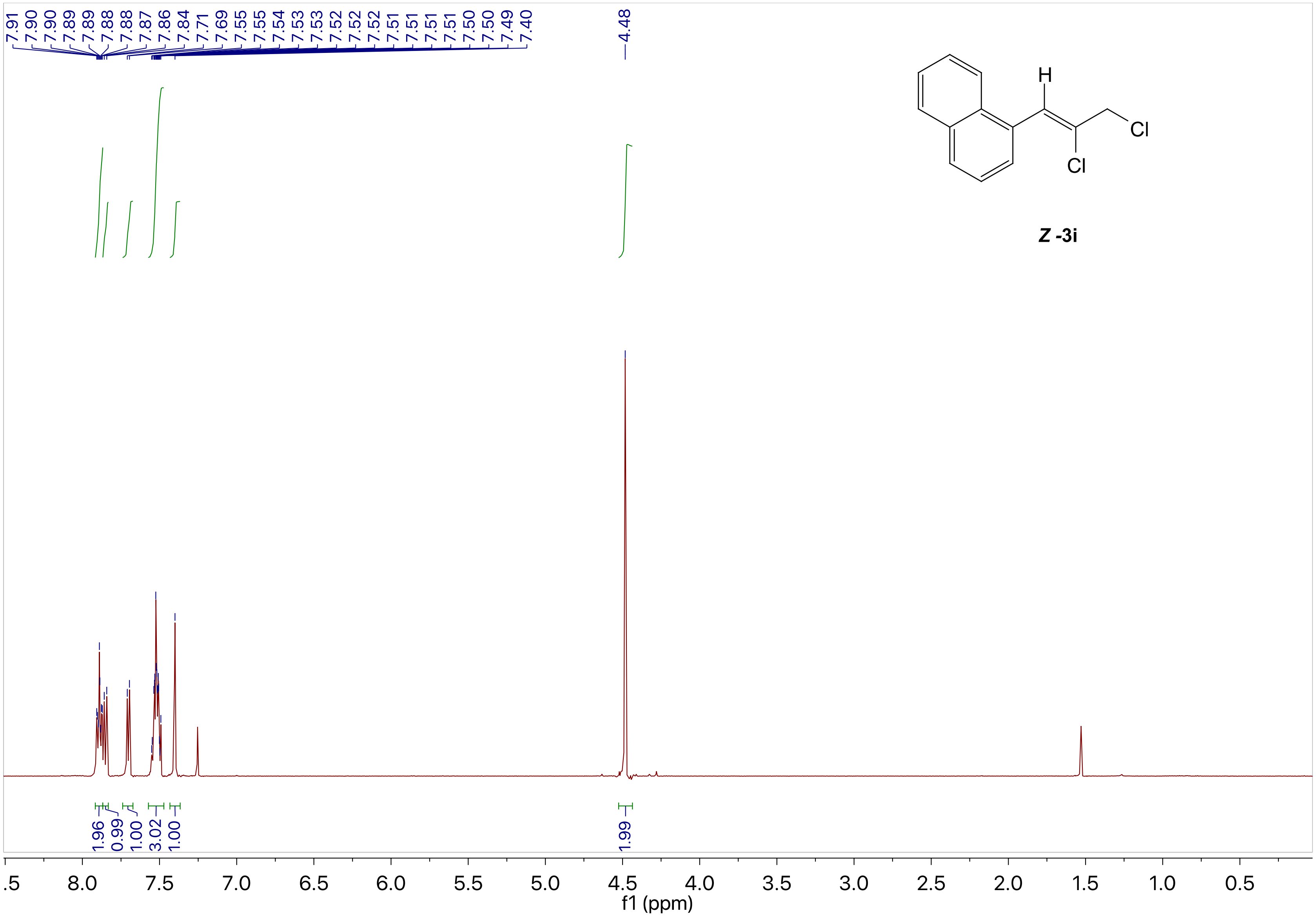
7.91
7.90
7.90
7.89
7.89
7.88
7.88
7.87
7.86
7.84
7.71
7.69
7.55
7.55
7.54
7.53
7.53
7.52
7.52
7.52
7.51
7.51
7.51
7.51
7.50
7.50
7.49
7.40
4.48
1.96
0.99
1.00
3.02
1.00
1.99
f1 (ppm)
H
Cl
Cl
Z -3i

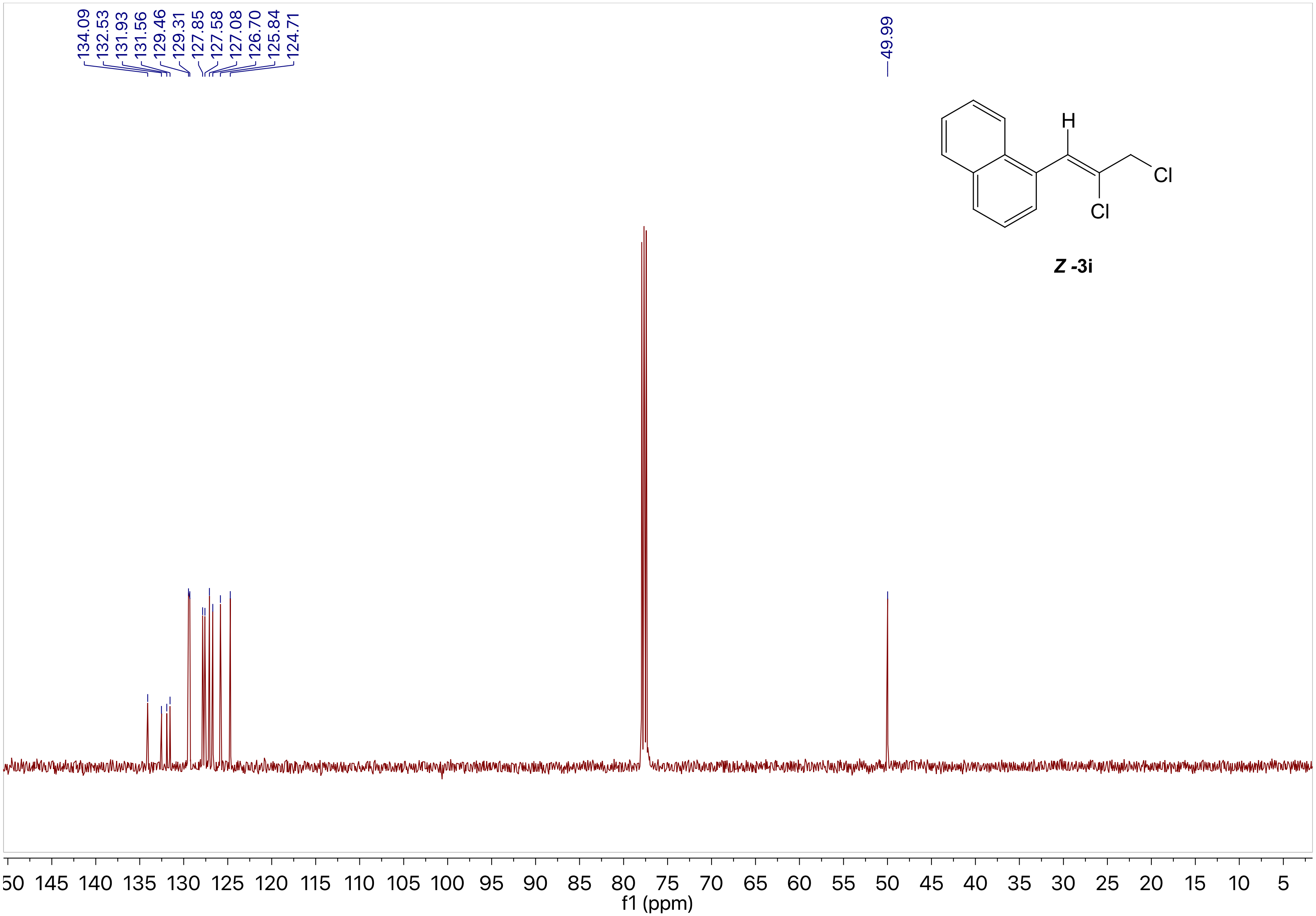
134.09
132.53
131.93
131.56
129.46
129.31
127.85
127.58
127.08
126.70
125.84
124.71
49.99
H
Cl
Cl
Z -3i
50 145 140 135 130 125 120 115 110 105 100 95 90 85 80 75 70 65 60 55 50 45 40 35 30 25 20 15 10 5
f1 (ppm)

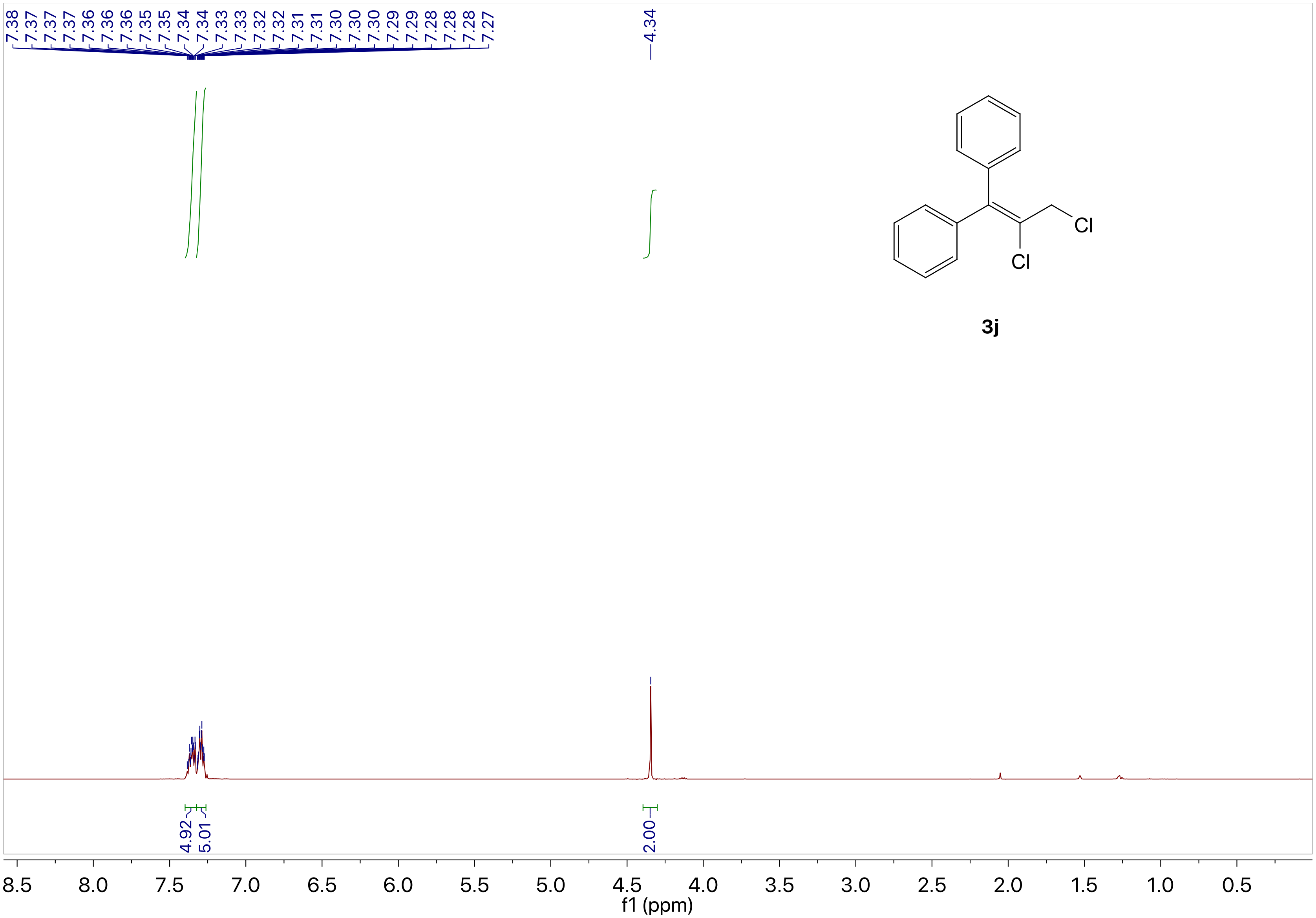
7.38
7.37
7.37
7.37
7.36
7.36
7.36
7.35
7.35
7.34
7.34
7.33
7.33
7.32
7.32
7.31
7.31
7.30
7.30
7.30
7.29
7.29
7.28
7.28
7.28
7.27
4.34
Cl
Cl
4.92
5.01
2.00
8.5
8.0
7.5
7.0
6.5
6.0
5.5
5.0
4.5
4.0
3.5
3.0
2.5
2.0
1.5
1.0
0.5
f1 (ppm)

3j

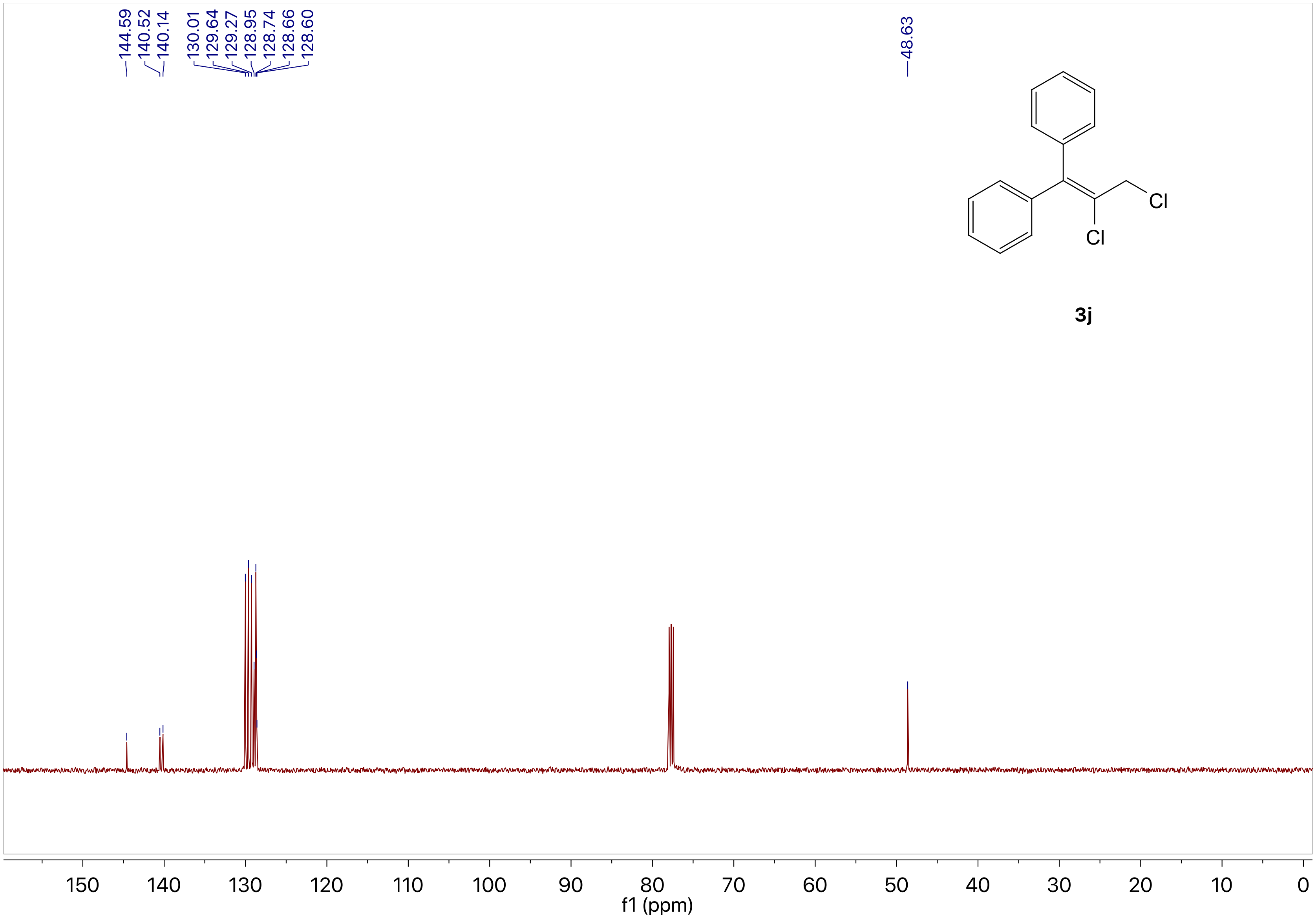
144.59
140.52
140.14
130.01
129.64
129.27
128.95
128.74
128.66
128.60
48.63
Cl
Cl
150
140
130
120
110
100
90
80
70
60
50
40
30
20
10
0
f1 (ppm)

3j

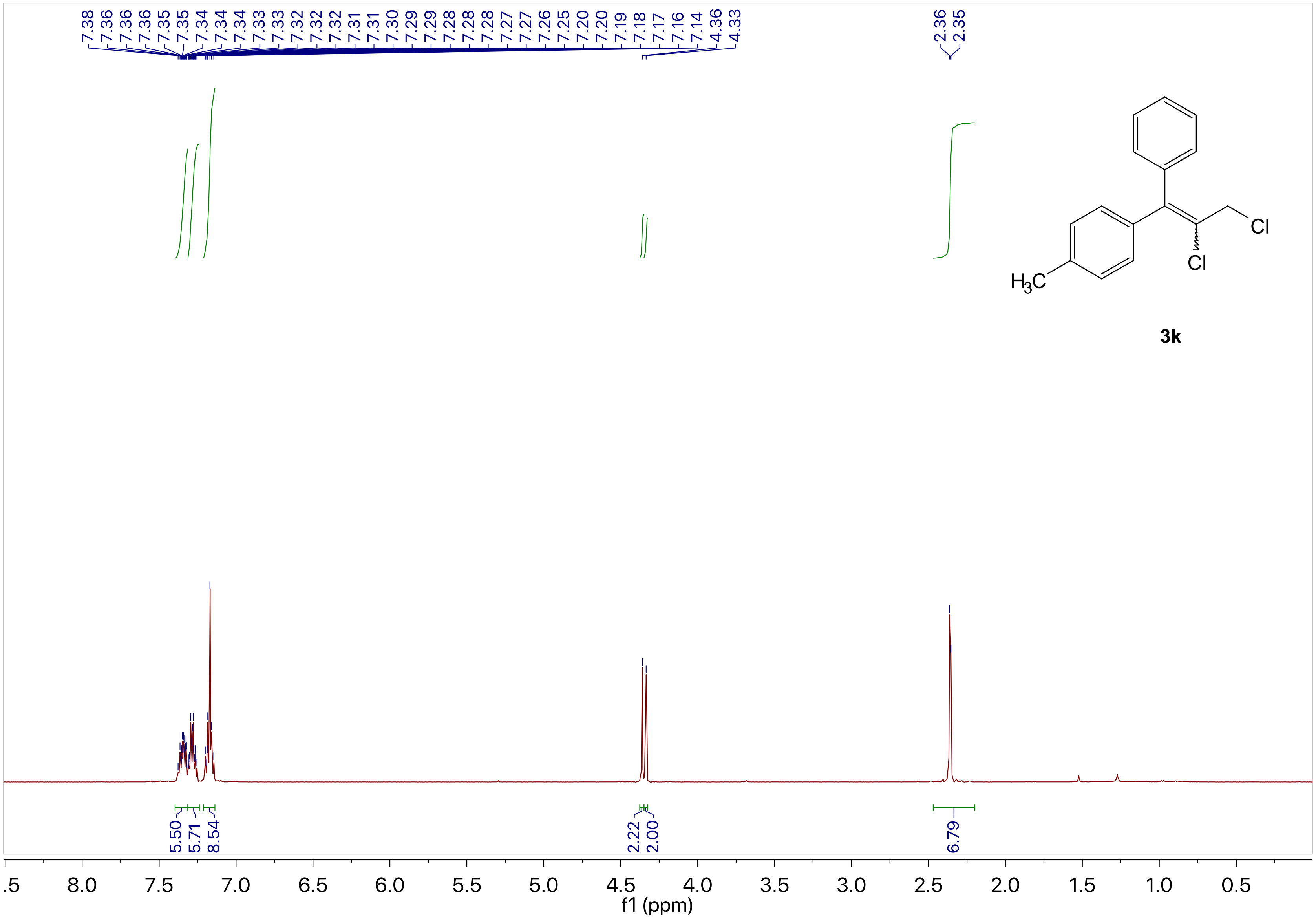
7.38
7.36
7.36
7.36
7.35
7.35
7.34
7.34
7.34
7.33
7.33
7.32
7.32
7.32
7.31
7.31
7.30
7.29
7.29
7.28
7.28
7.28
7.27
7.27
7.26
7.25
7.20
7.20
7.19
7.18
7.17
7.16
7.14
4.36
4.33
2.36
2.35
H3C
Cl
Cl
5.50
5.71
8.54
2.22
2.00
6.79
.5
8.0
7.5
7.0
6.5
6.0
5.5
5.0
4.5
4.0
3.5
3.0
2.5
2.0
1.5
1.0
0.5
f1 (ppm)

3k

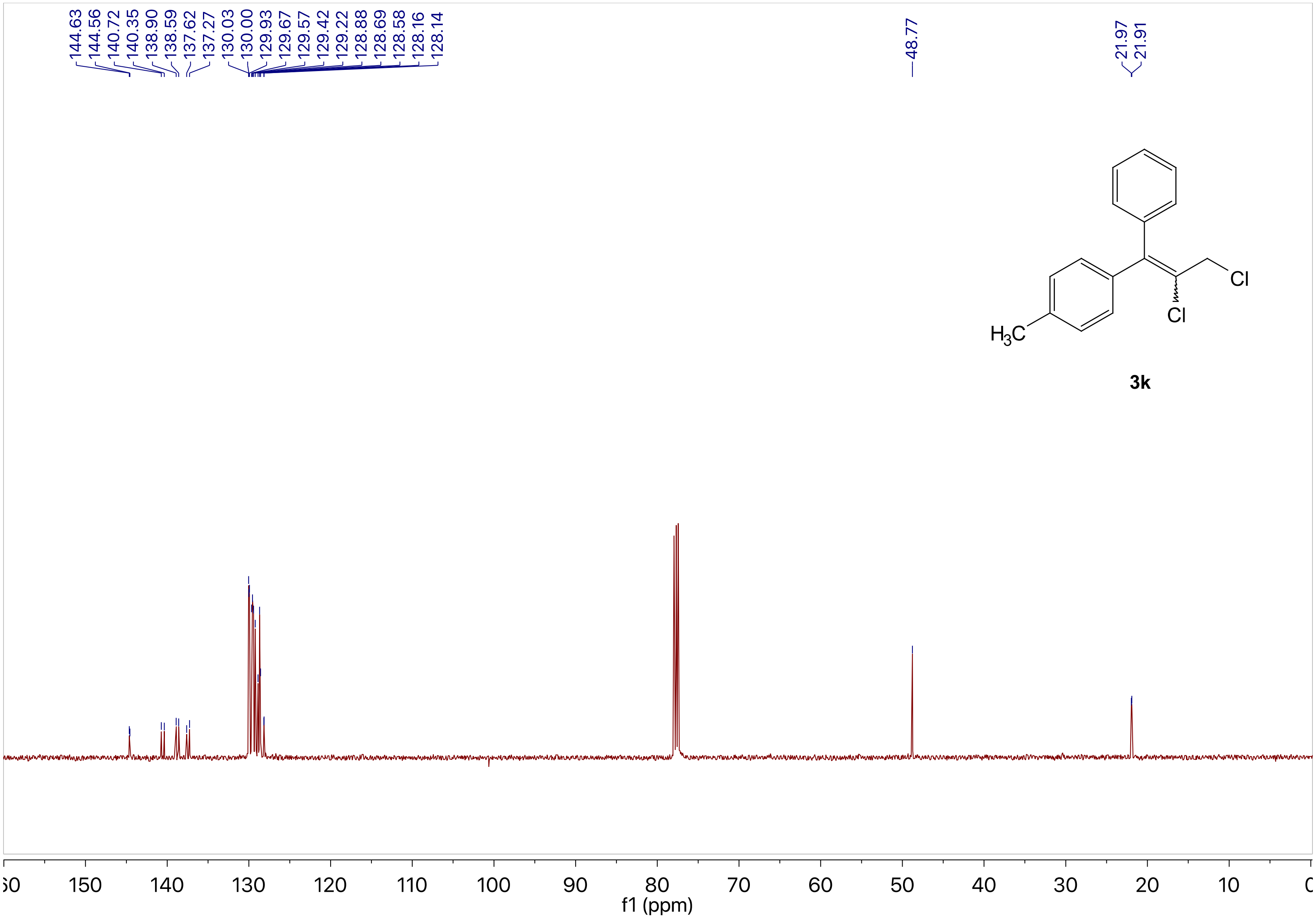
144.63
144.56
140.72
140.35
138.90
138.59
137.62
137.27
130.03
130.00
129.93
129.67
129.57
129.42
129.22
128.88
128.69
128.58
128.16
128.14
48.77
21.97
21.91
Cl
Cl
H_3C
3k
150
140
130
120
110
100
90
80
70
60
50
40
30
20
10
f1 (ppm)

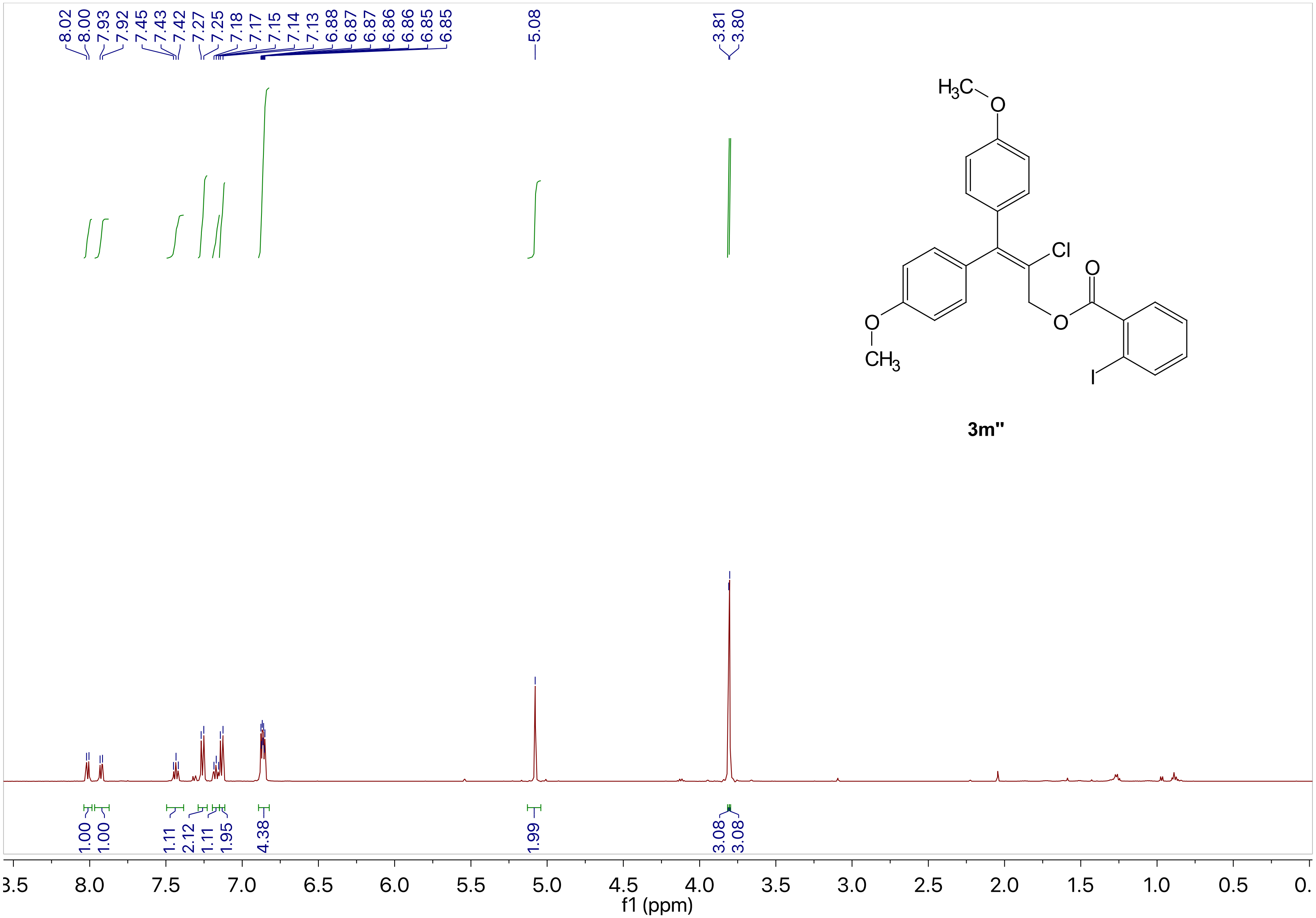
8.02
8.00
7.93
7.92
7.45
7.43
7.42
7.27
7.25
7.18
7.17
7.15
7.14
7.13
6.88
6.87
6.87
6.86
6.86
6.85
6.85
5.08
3.81
3.80
1.00
1.00
1.11
2.12
1.11
1.95
4.38
1.99
3.08
3.08
H3C
O
O
CH3
Cl
O
O
I
3m''
f1 (ppm)

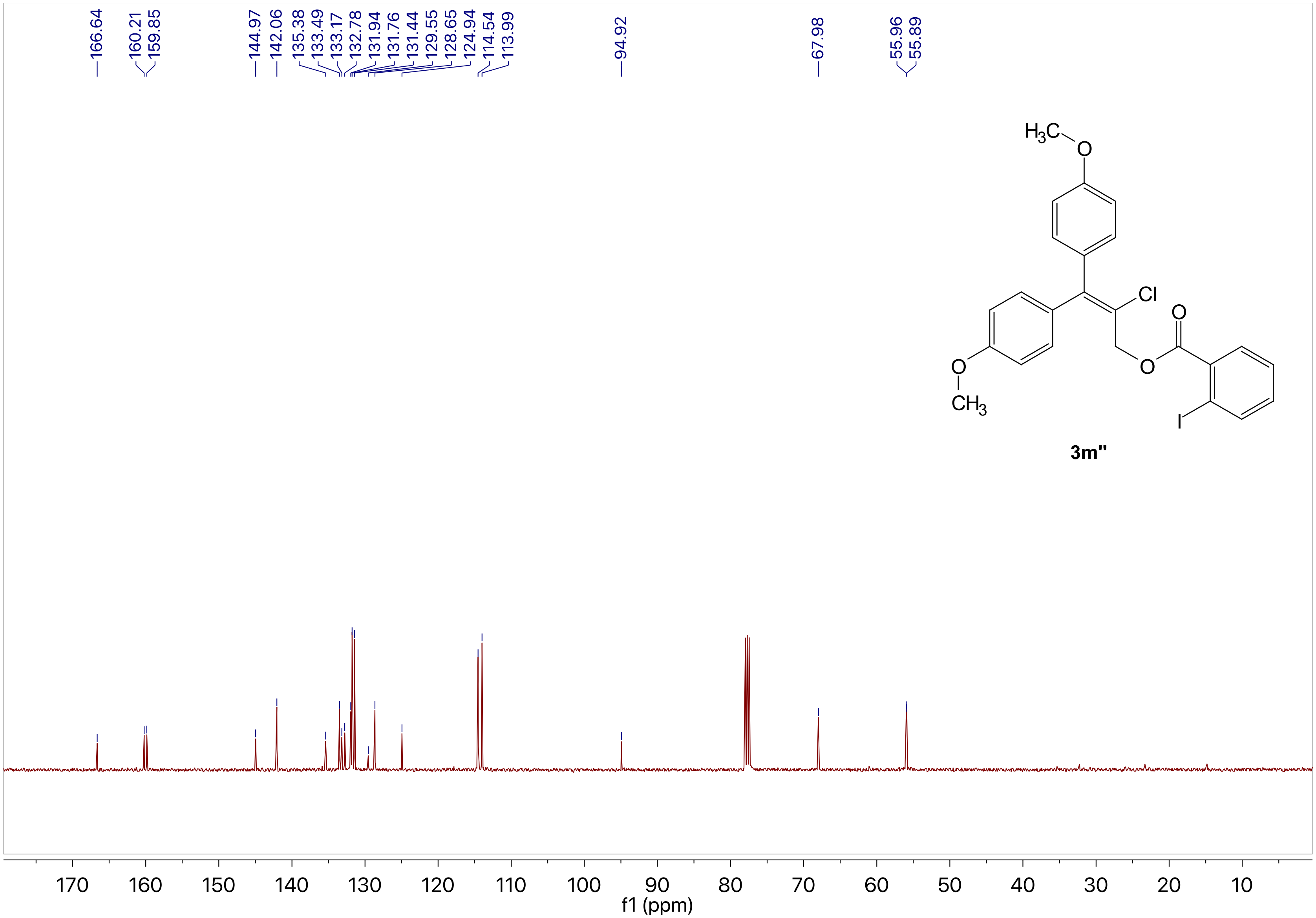
166.64
160.21
159.85
144.97
142.06
135.38
133.49
133.17
132.78
131.94
131.76
131.44
129.55
128.65
124.94
114.54
113.99
94.92
67.98
55.96
55.89
H3C
O
Cl
O
O
O
CH3
I
3m''
170
160
150
140
130
120
110
100
90
80
70
60
50
40
30
20
10
f1 (ppm)

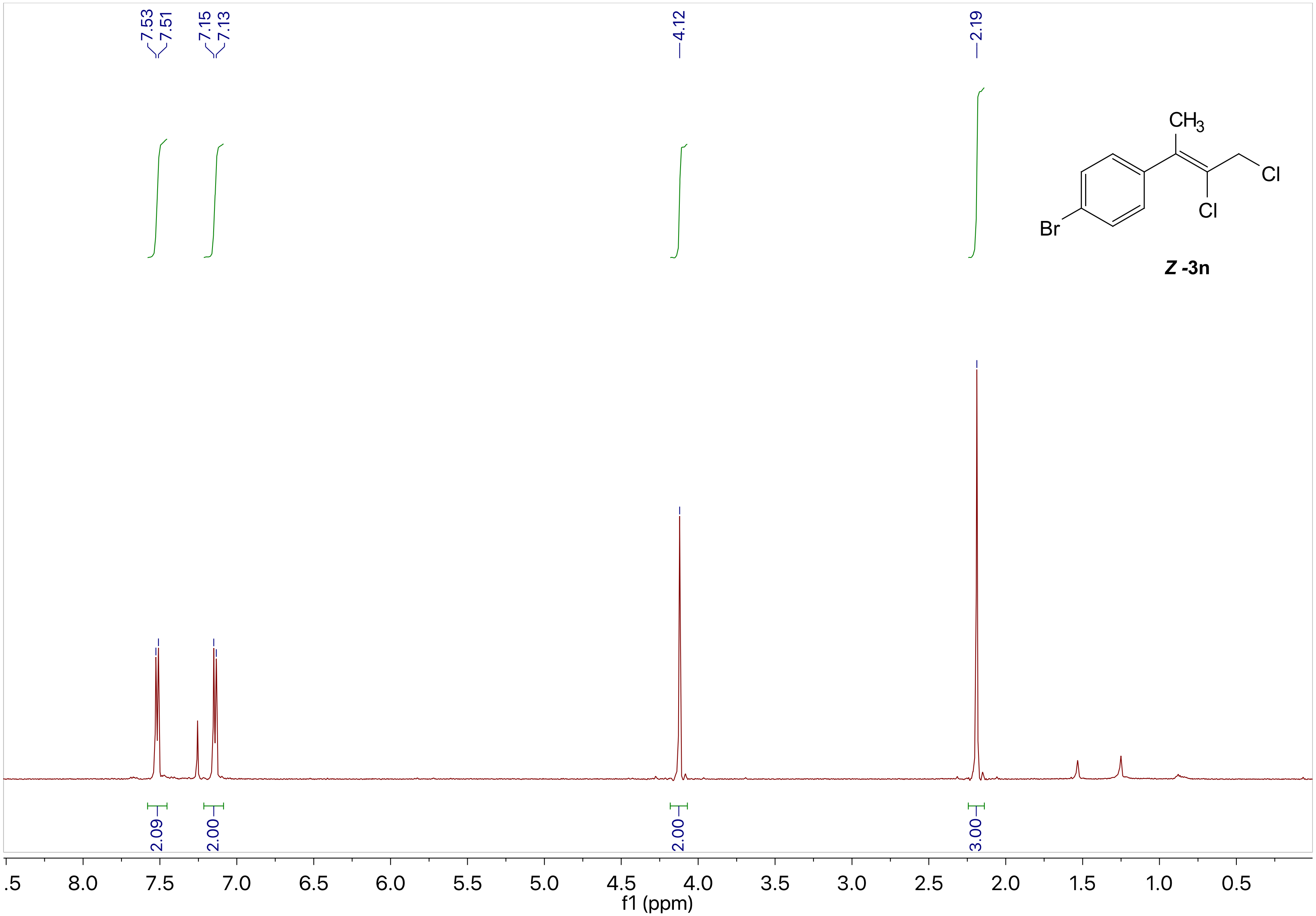
7.53
7.51
7.15
7.13
4.12
2.19
CH3
Cl
Cl
Br
Z -3n
2.09
2.00
2.00
3.00
.5
8.0
7.5
7.0
6.5
6.0
5.5
5.0
4.5
4.0
3.5
3.0
2.5
2.0
1.5
1.0
0.5
f1 (ppm)

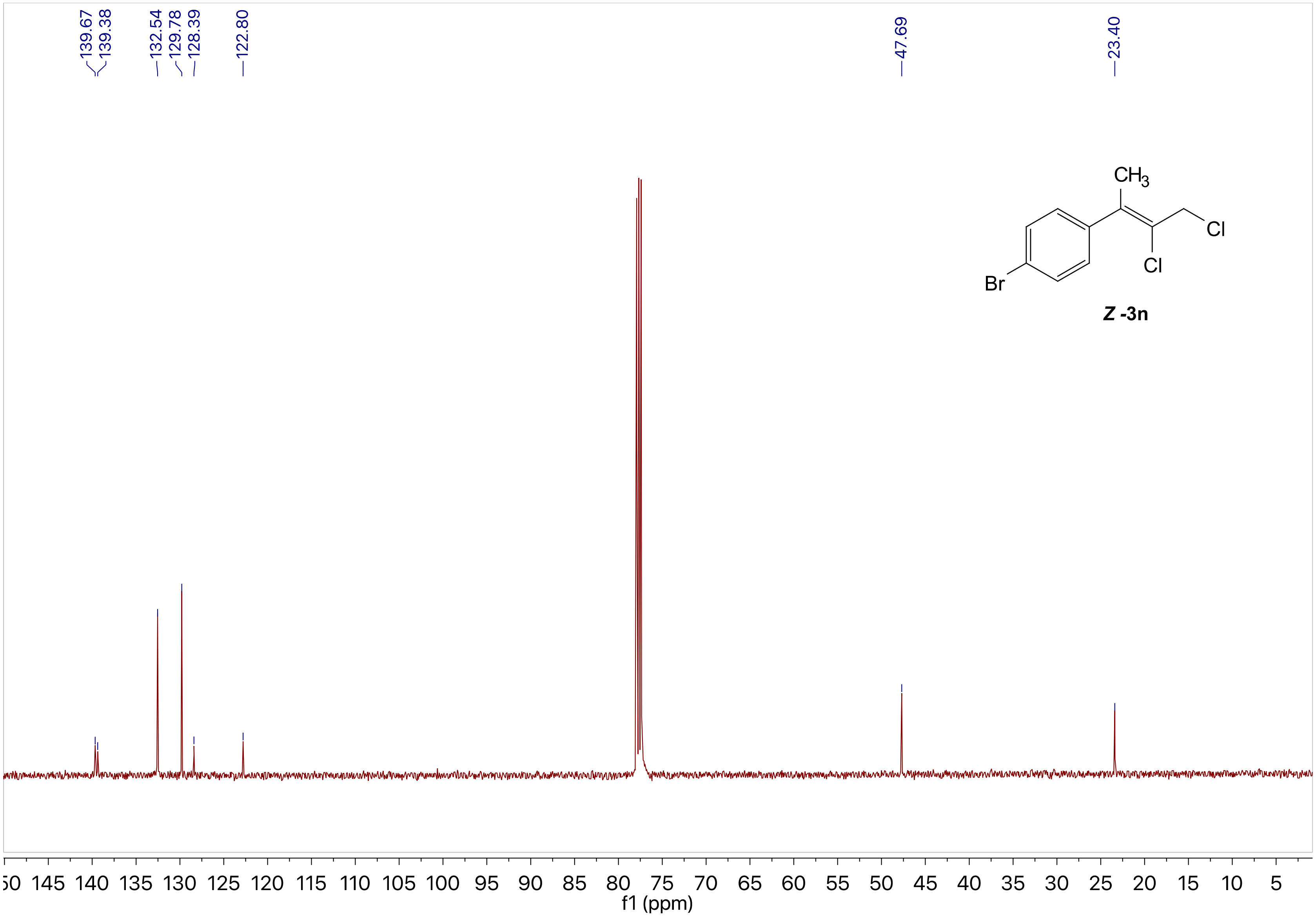

139.67
139.38
132.54
129.78
128.39
122.80
47.69
23.40
CH3
Cl
Cl
Br
Z -3n
50 145 140 135 130 125 120 115 110 105 100 95 90 85 80 75 70 65 60 55 50 45 40 35 30 25 20 15 10 5
f1 (ppm)

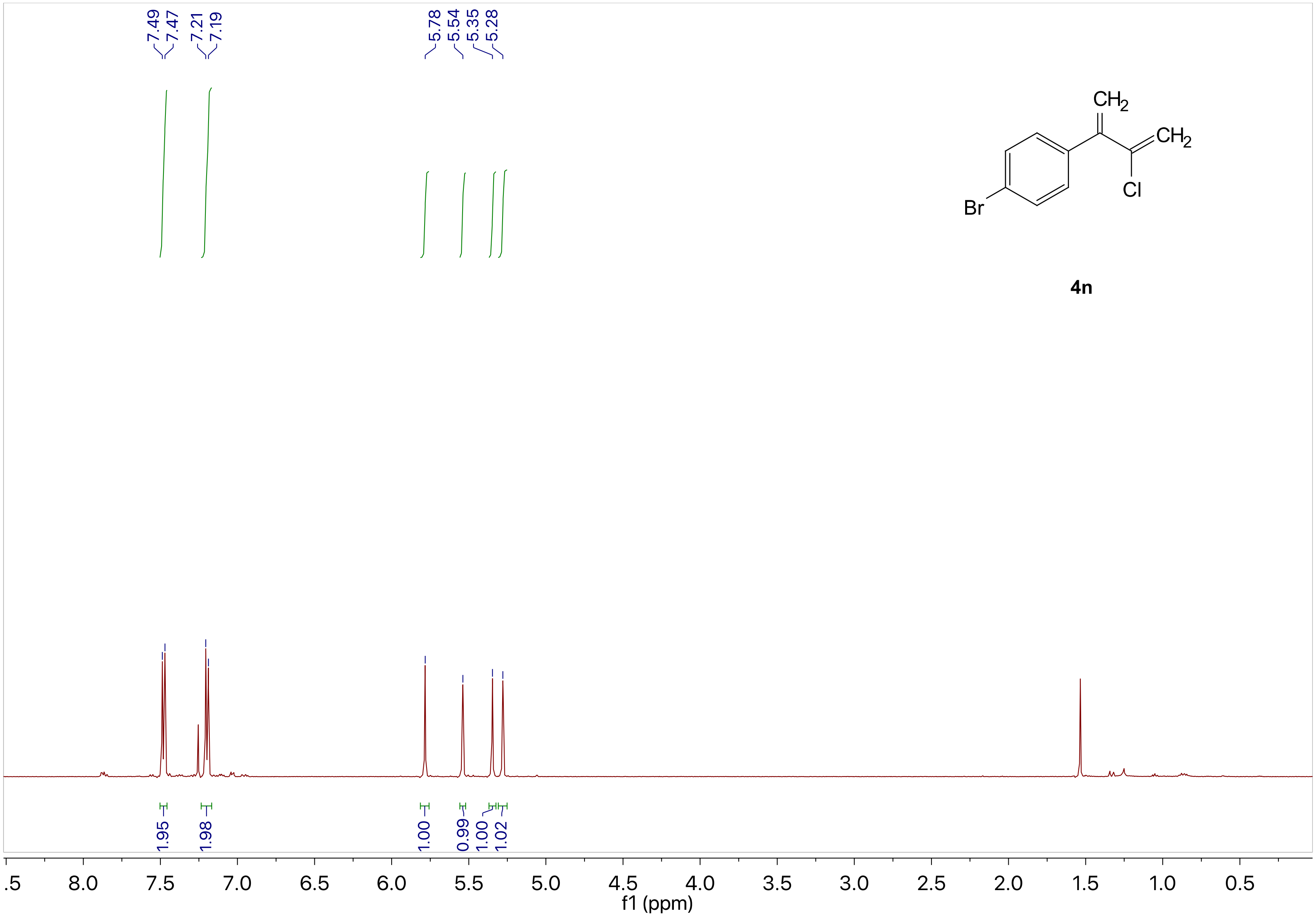
7.49
7.47
7.21
7.19
5.78
5.54
5.35
5.28
CH2
CH2
Br
Cl
4n
1.95
1.98
1.00
0.99
1.00
1.02
.5
8.0
7.5
7.0
6.5
6.0
5.5
5.0
4.5
4.0
3.5
3.0
2.5
2.0
1.5
1.0
0.5
f1 (ppm)

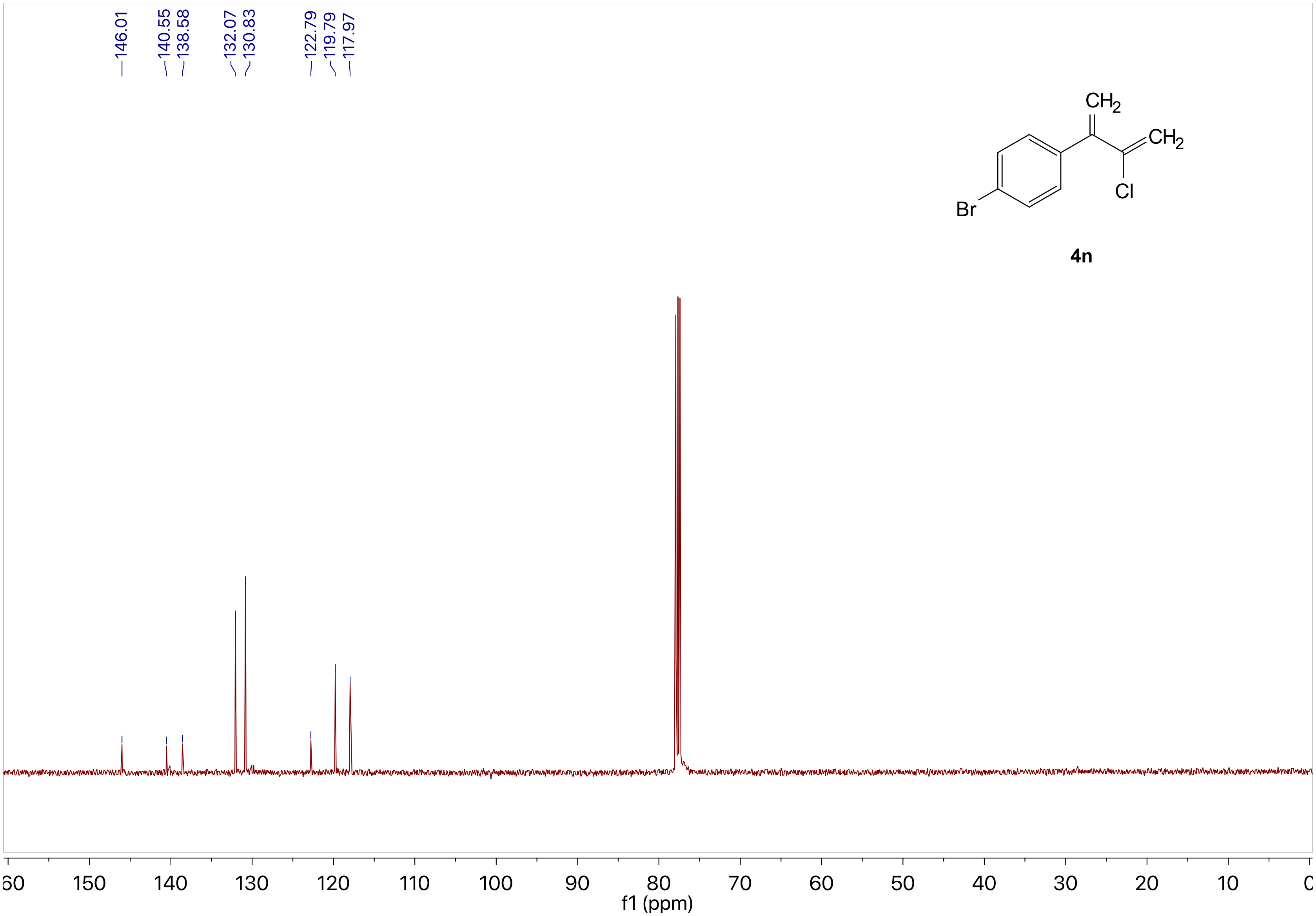

146.01
140.55
138.58
132.07
130.83
122.79
119.79
117.97
CH2
CH2
Cl
Br
4n
160
150
140
130
120
110
100
90
80
70
60
50
40
30
20
10
0
f1 (ppm)

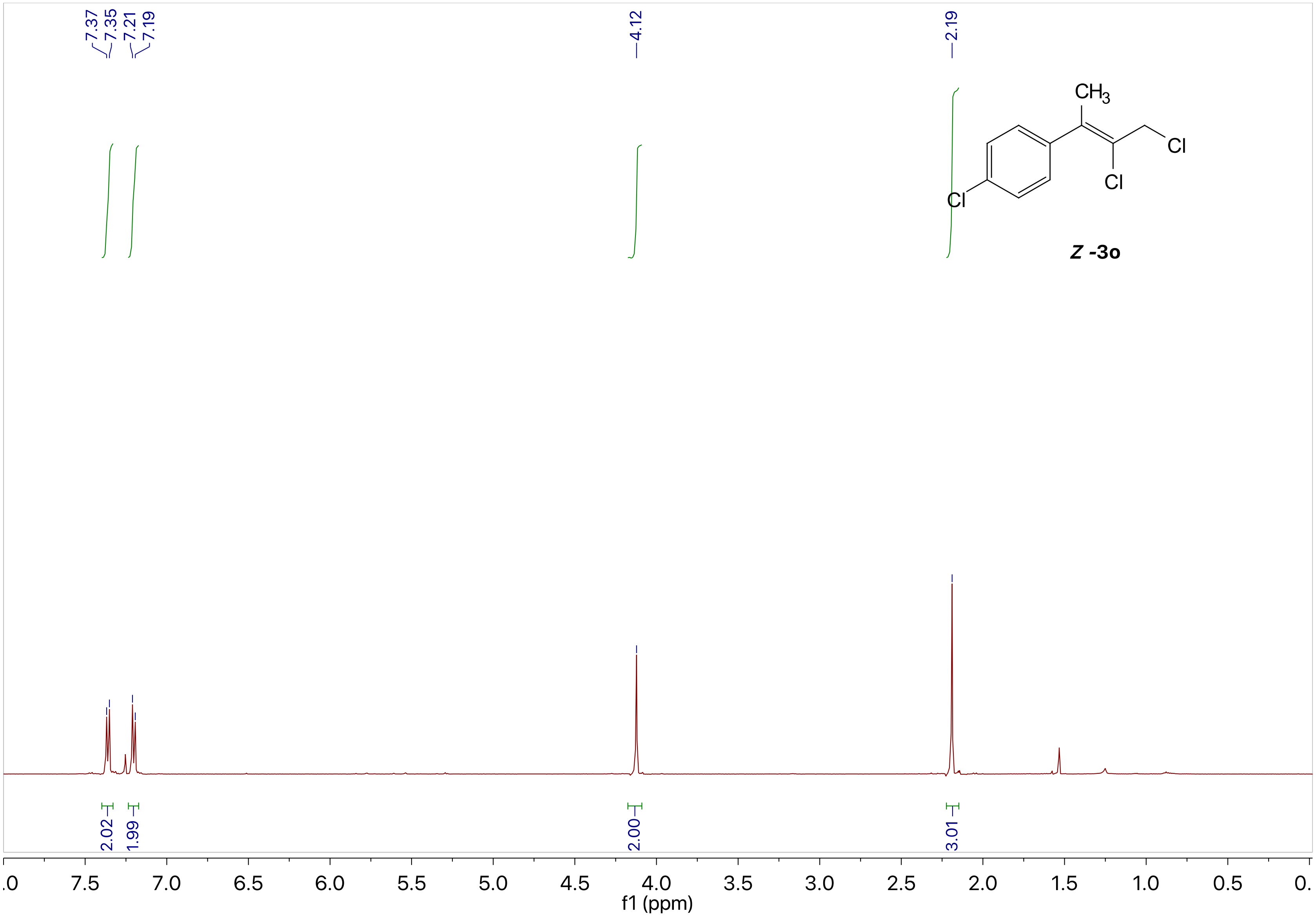
7.37
7.35
7.21
7.19
4.12
2.19
CH3
Cl
Cl
Cl
Z -3o
2.02
1.99
2.00
3.01
7.5
7.0
6.5
6.0
5.5
5.0
4.5
4.0
3.5
3.0
2.5
2.0
1.5
1.0
0.5
f1 (ppm)

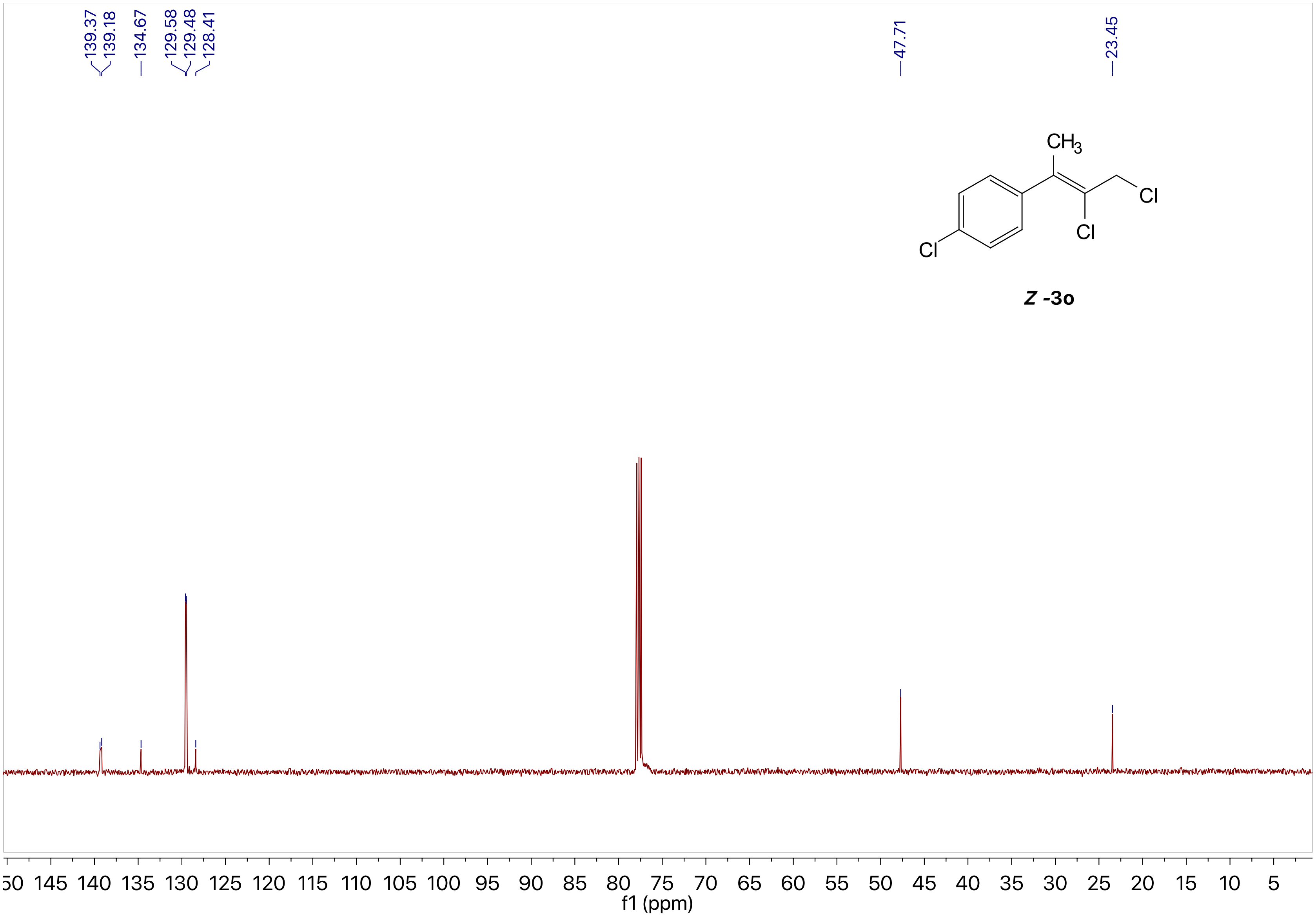
139.37
139.18
134.67
129.58
129.48
128.41
47.71
23.45
CH3
Cl
Cl
Cl
Z -3o
50 145 140 135 130 125 120 115 110 105 100 95 90 85 80 75 70 65 60 55 50 45 40 35 30 25 20 15 10 5
f1 (ppm)

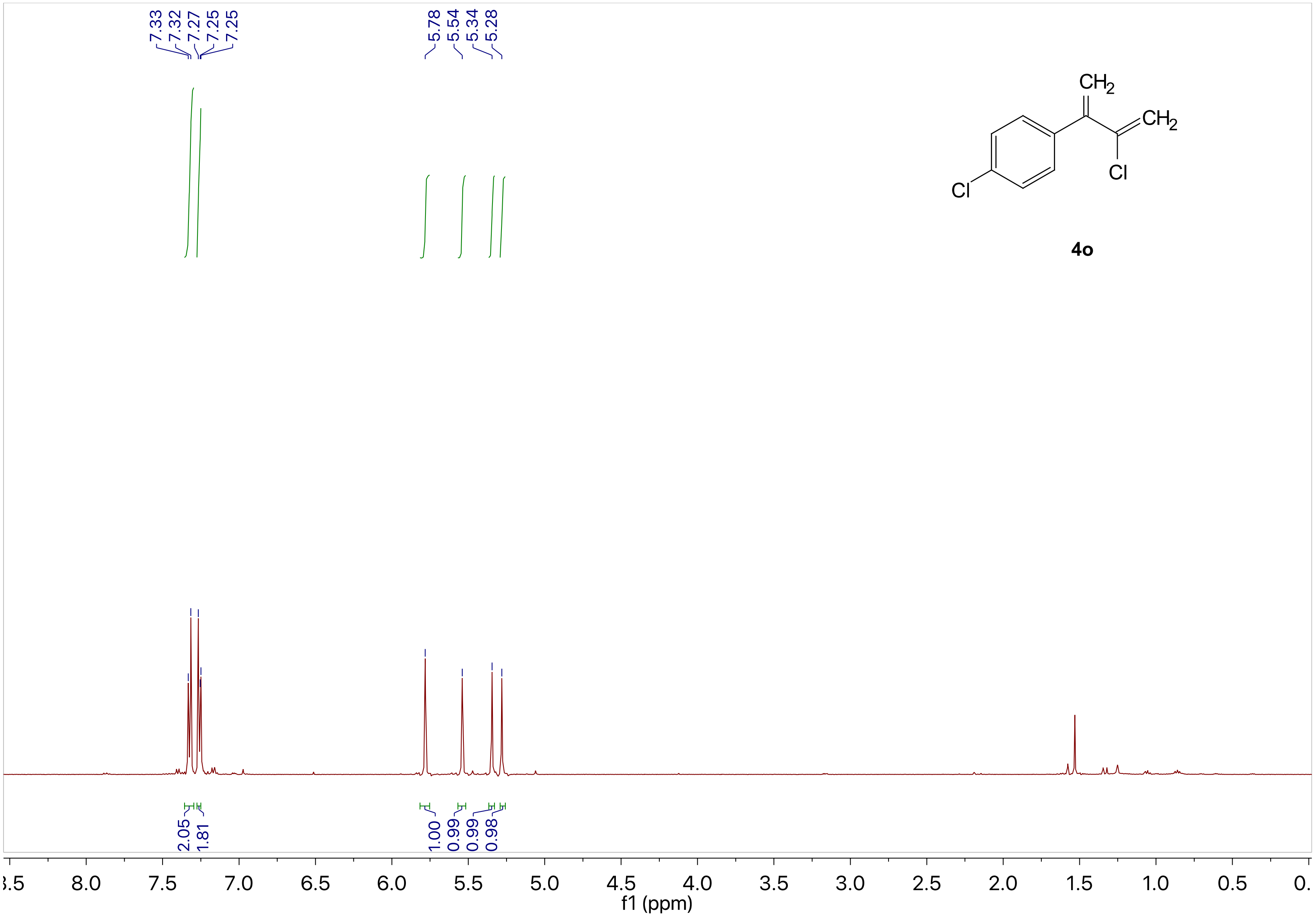
7.33
7.32
7.27
7.25
7.25
5.78
5.54
5.34
5.28
CH2
CH2
Cl
Cl
4o
2.05
1.81
1.00
0.99
0.99
0.98
8.0
7.5
7.0
6.5
6.0
5.5
5.0
4.5
4.0
3.5
3.0
2.5
2.0
1.5
1.0
0.5
f1 (ppm)

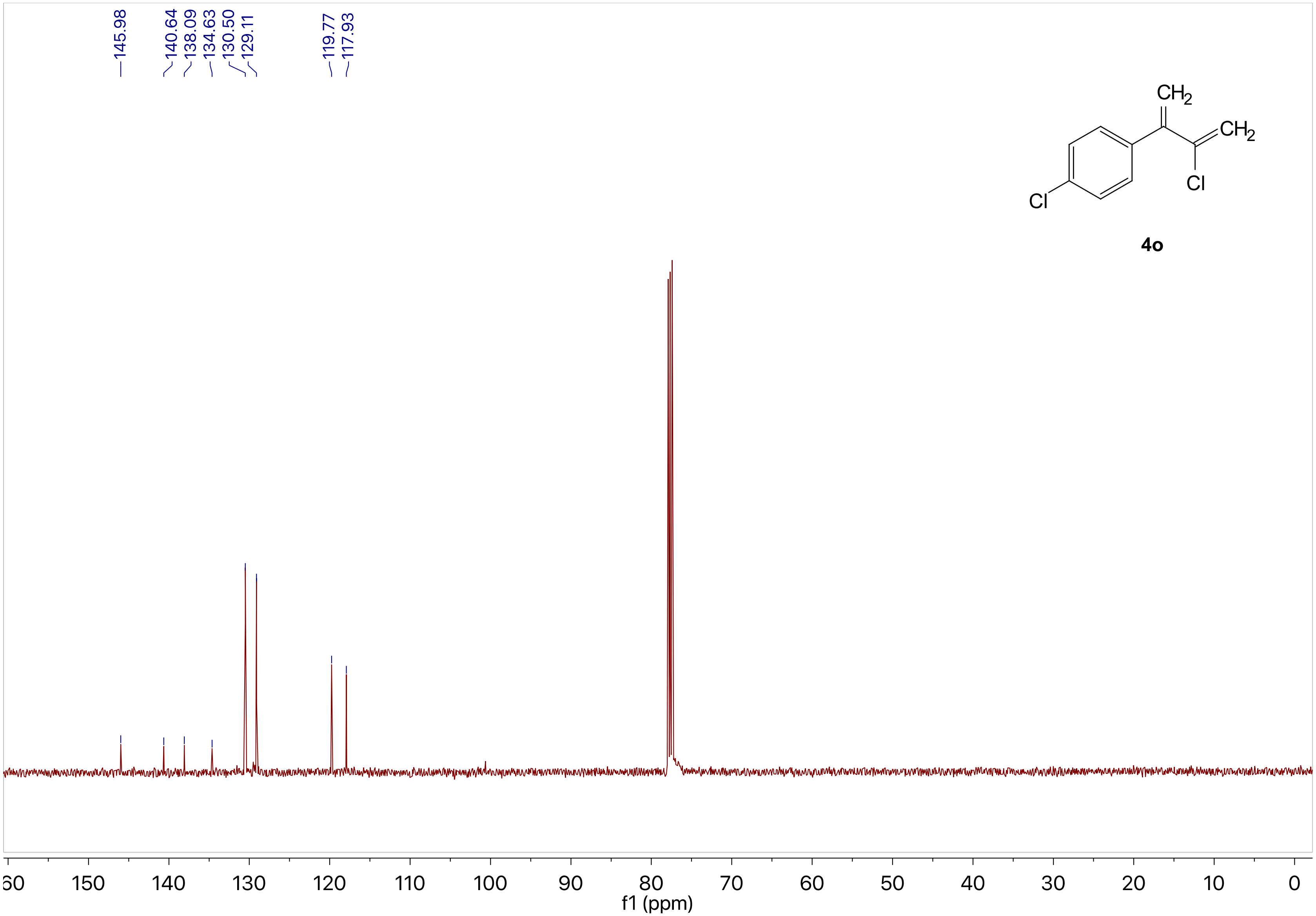
145.98
140.64
138.09
134.63
130.50
129.11
119.77
117.93
CH2
CH2
Cl
Cl
4o
160
150
140
130
120
110
100
90
80
70
60
50
40
30
20
10
0
f1 (ppm)

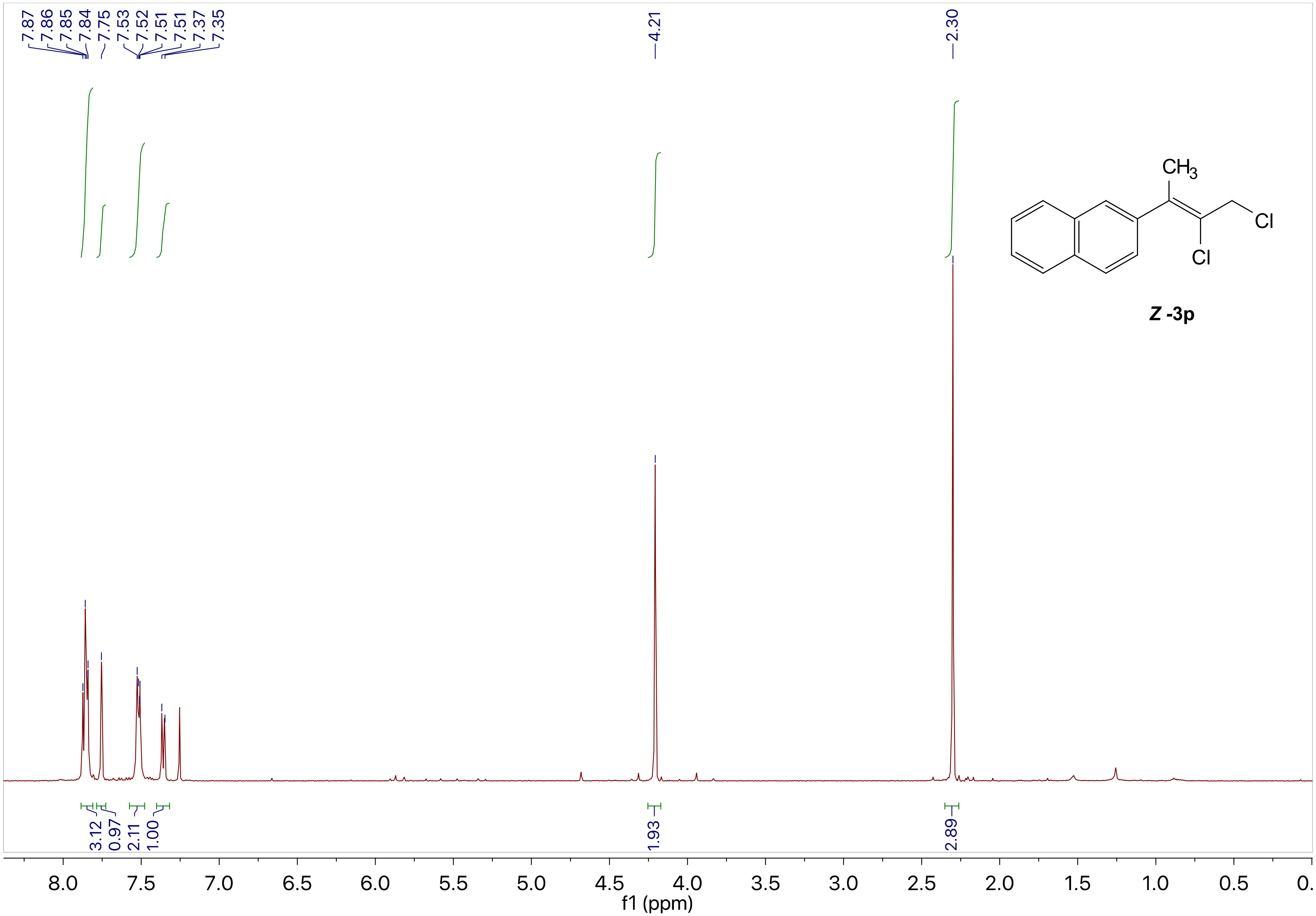
7.87
7.86
7.85
7.84
7.75
7.53
7.52
7.51
7.51
7.37
7.35
4.21
2.30
CH3
Cl
Cl
Z -3p
3.12
0.97
2.11
1.00
1.93
2.89
8.0
7.5
7.0
6.5
6.0
5.5
5.0
4.5
4.0
3.5
3.0
2.5
2.0
1.5
1.0
0.5
0.
f1 (ppm)

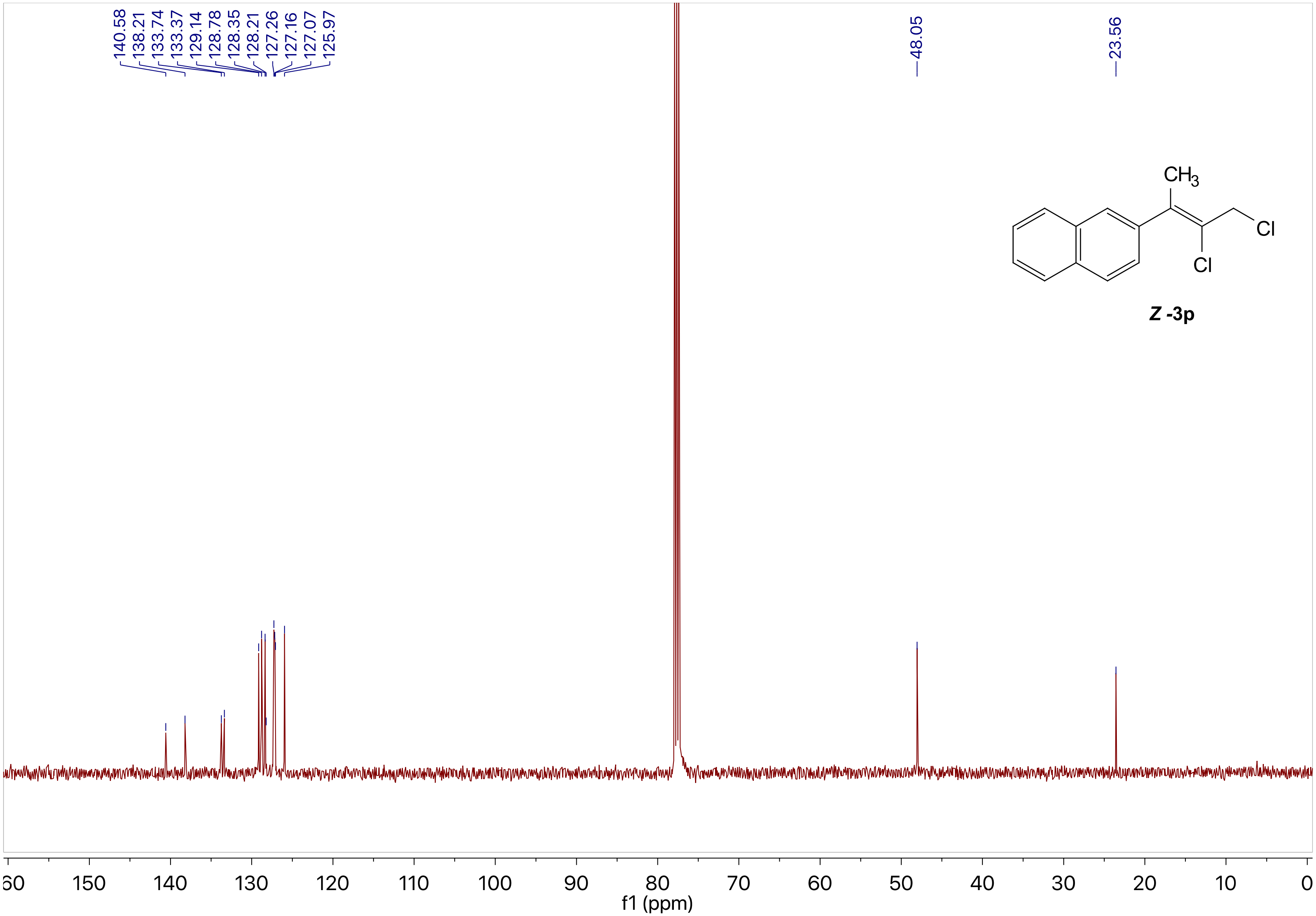
140.58
138.21
133.74
133.37
129.14
128.78
128.35
128.21
127.26
127.16
127.07
125.97
48.05
23.56
CH3
Cl
Cl
f1 (ppm)
150
140
130
120
110
100
90
80
70
60
50
40
30
20
10
0

***Z*-3p**

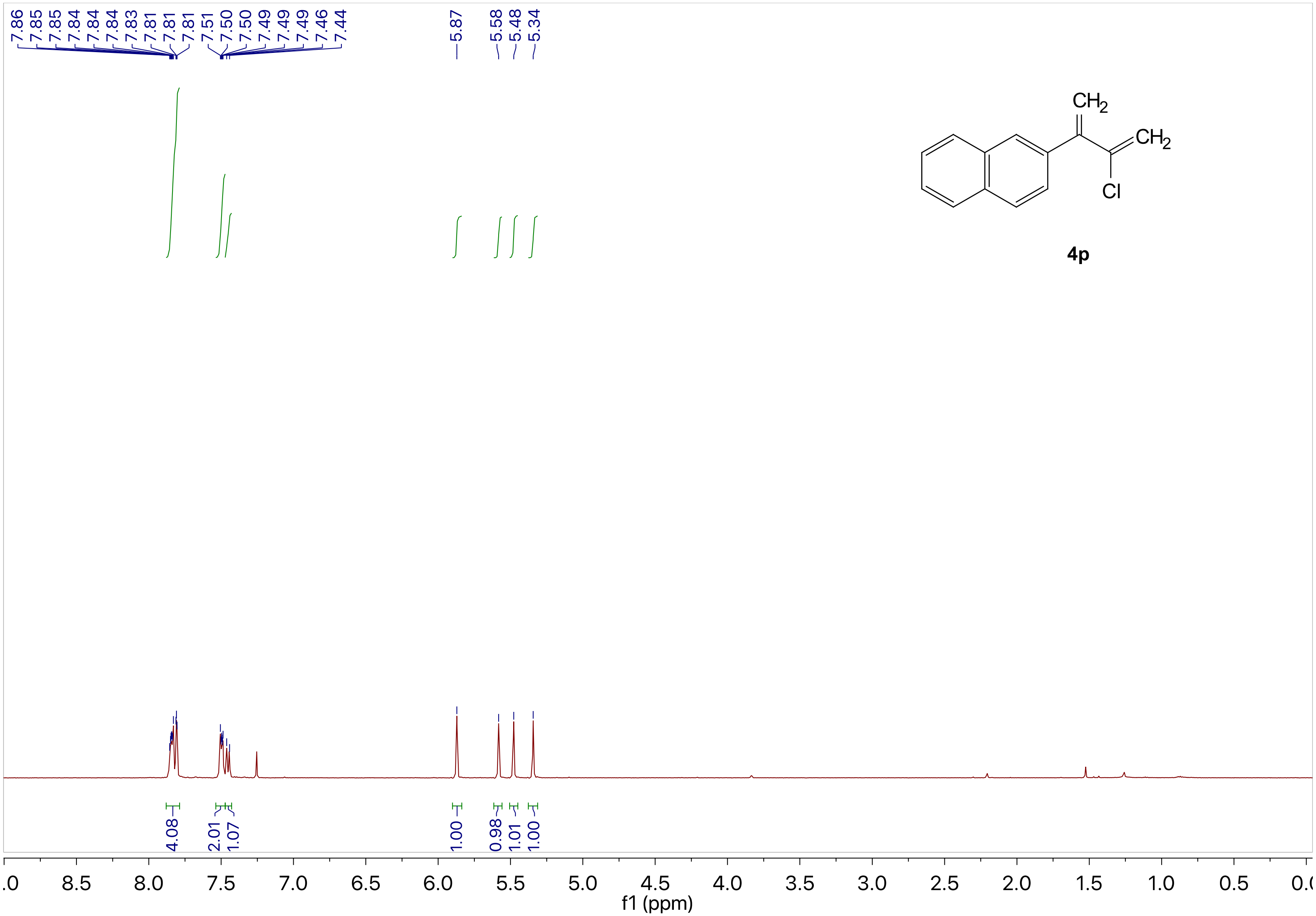
7.86 7.85 7.85 7.84 7.84 7.84 7.83 7.81 7.81 7.81 7.51 7.50 7.50 7.49 7.49 7.49 7.46 7.44
5.87
5.58 5.48 5.34
CH2
CH2
Cl
4p
4.08
2.01 1.07
1.00
0.98 1.01 1.00
.0 8.5 8.0 7.5 7.0 6.5 6.0 5.5 5.0 4.5 4.0 3.5 3.0 2.5 2.0 1.5 1.0 0.5 0.(
f1 (ppm)

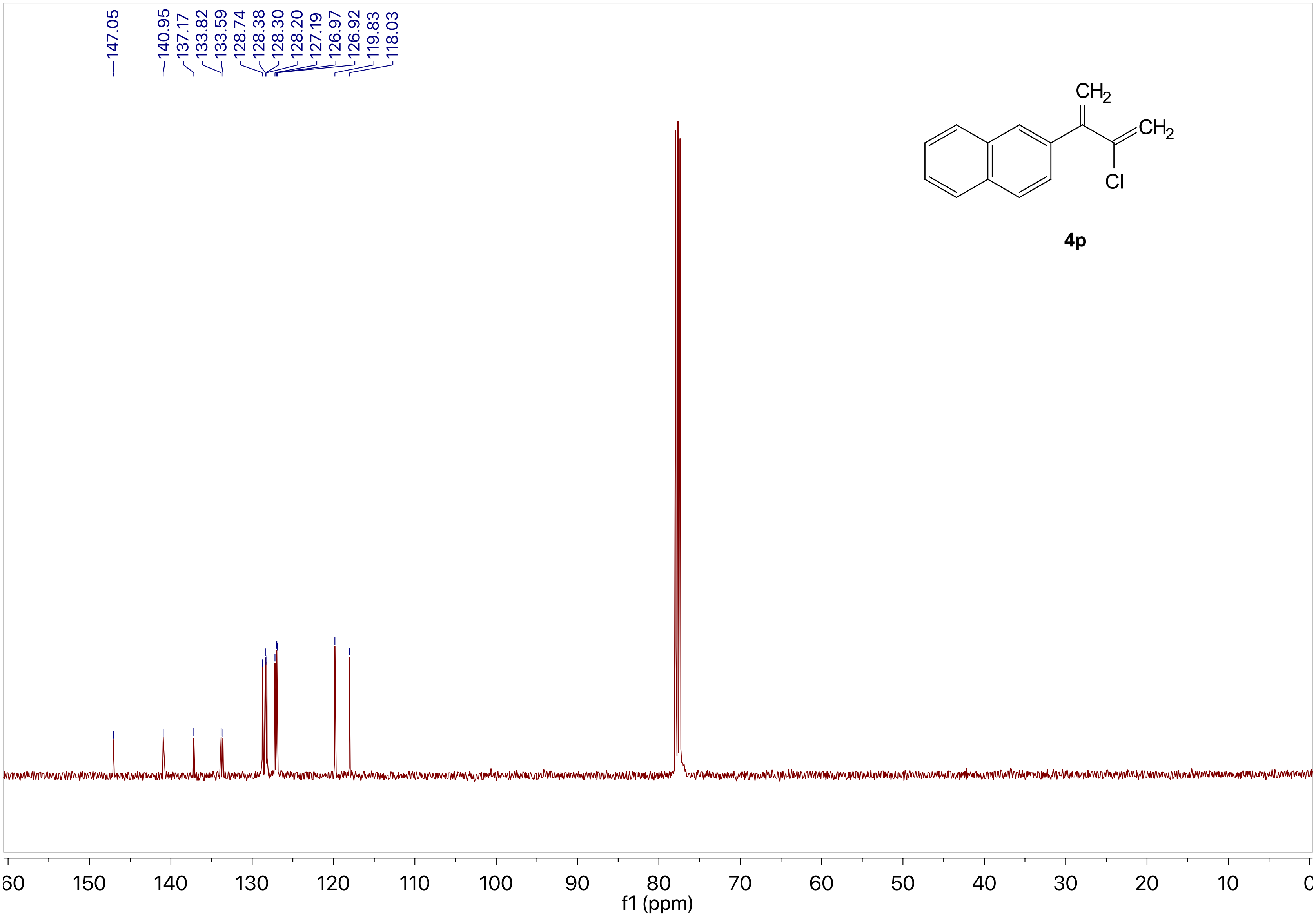
147.05
140.95
137.17
133.82
133.59
128.74
128.38
128.30
128.20
127.19
126.97
126.92
119.83
118.03
CH2
CH2
Cl
4p
160
150
140
130
120
110
100
90
80
70
60
50
40
30
20
10
0
f1 (ppm)

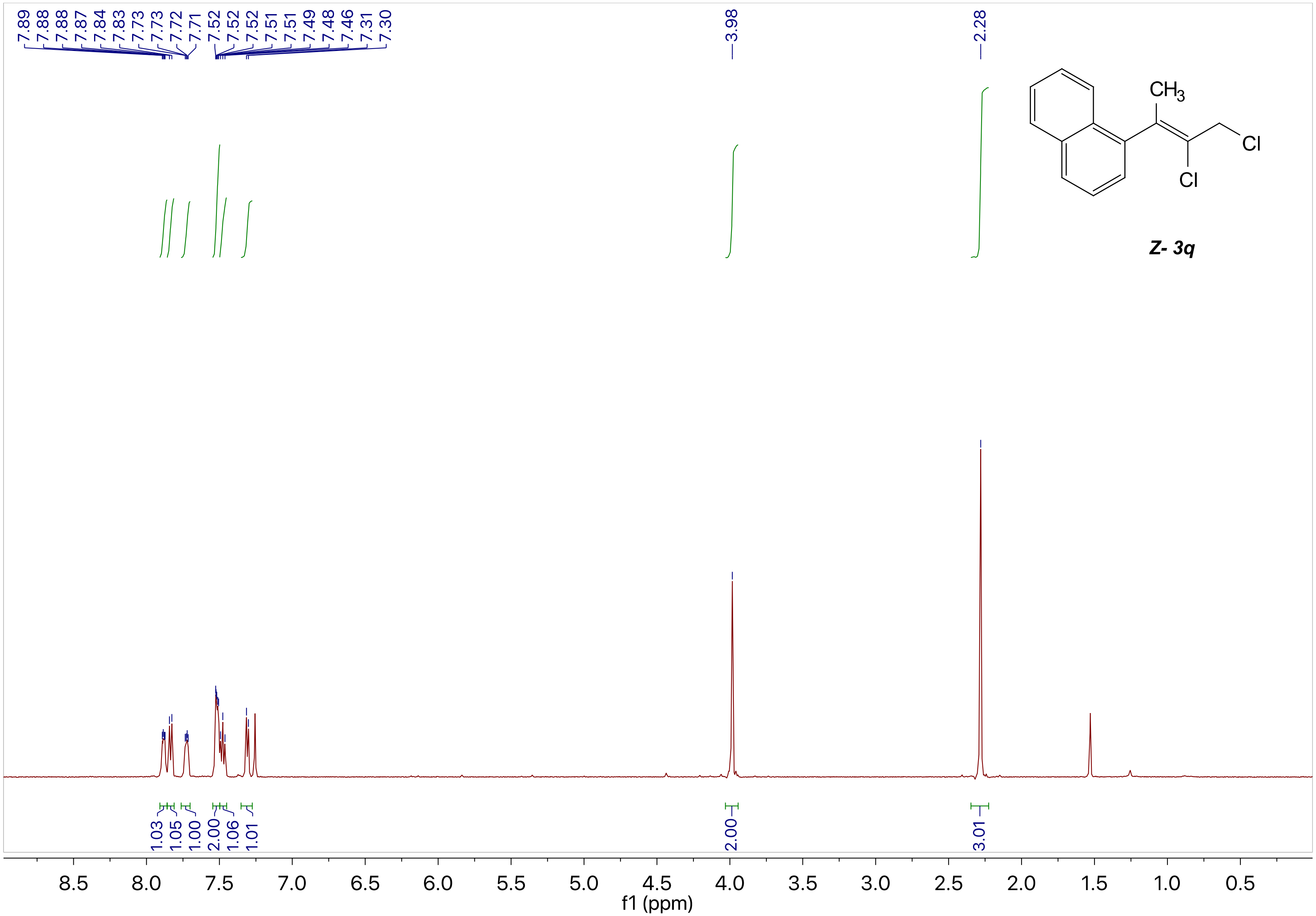
7.89
7.88
7.88
7.87
7.84
7.83
7.73
7.73
7.72
7.71
7.52
7.52
7.52
7.51
7.51
7.49
7.48
7.46
7.31
7.30
3.98
2.28
CH3
Cl
Cl
Z- 3q
1.03
1.05
1.00
2.00
1.06
1.01
2.00
3.01
8.5
8.0
7.5
7.0
6.5
6.0
5.5
5.0
4.5
4.0
3.5
3.0
2.5
2.0
1.5
1.0
0.5
f1 (ppm)

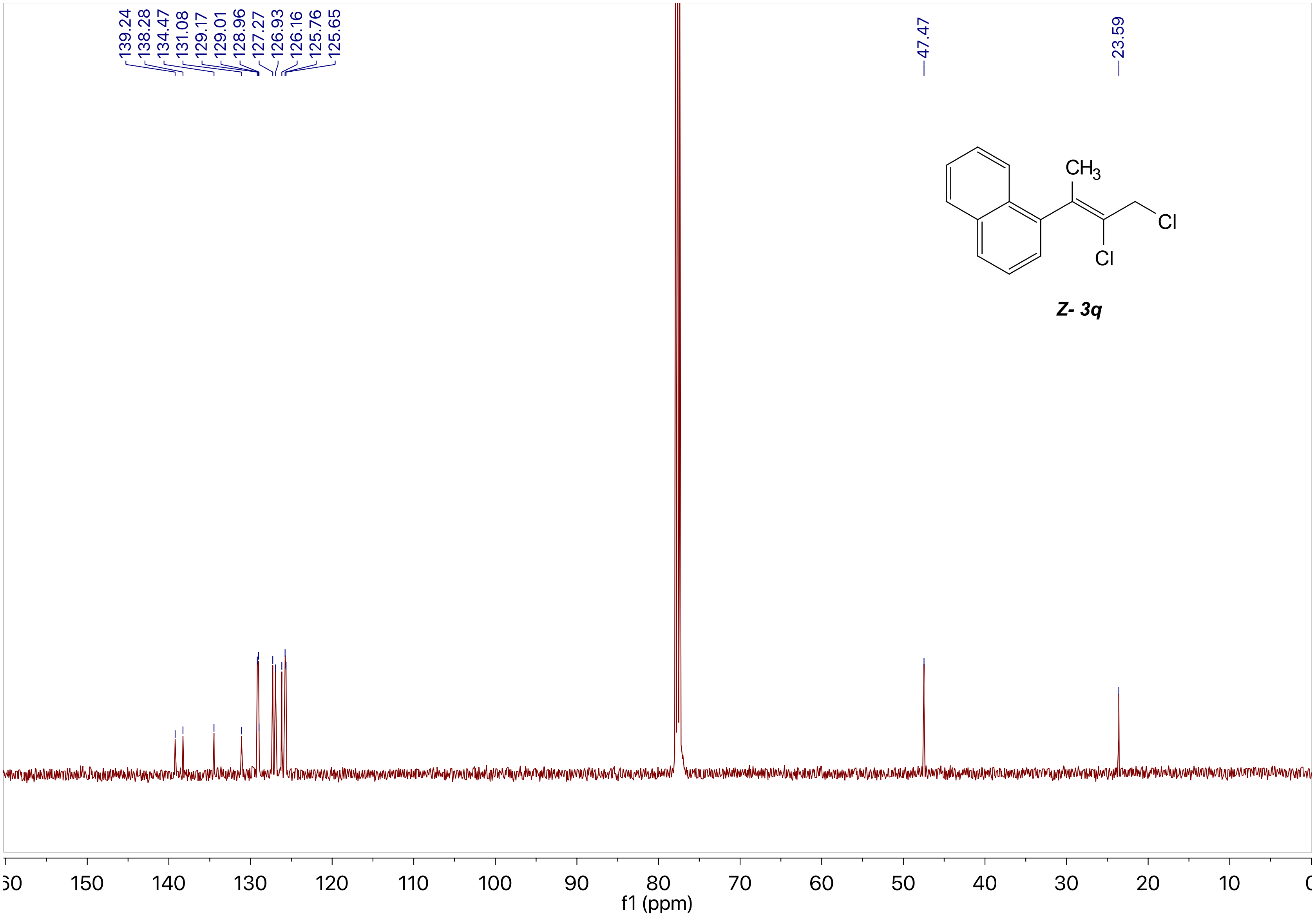
139.24
138.28
134.47
131.08
129.17
129.01
128.96
127.27
126.93
126.16
125.76
125.65
47.47
23.59
CH3
Cl
Cl
Z- 3q
150
140
130
120
110
100
90
80
70
60
50
40
30
20
10
f1 (ppm)

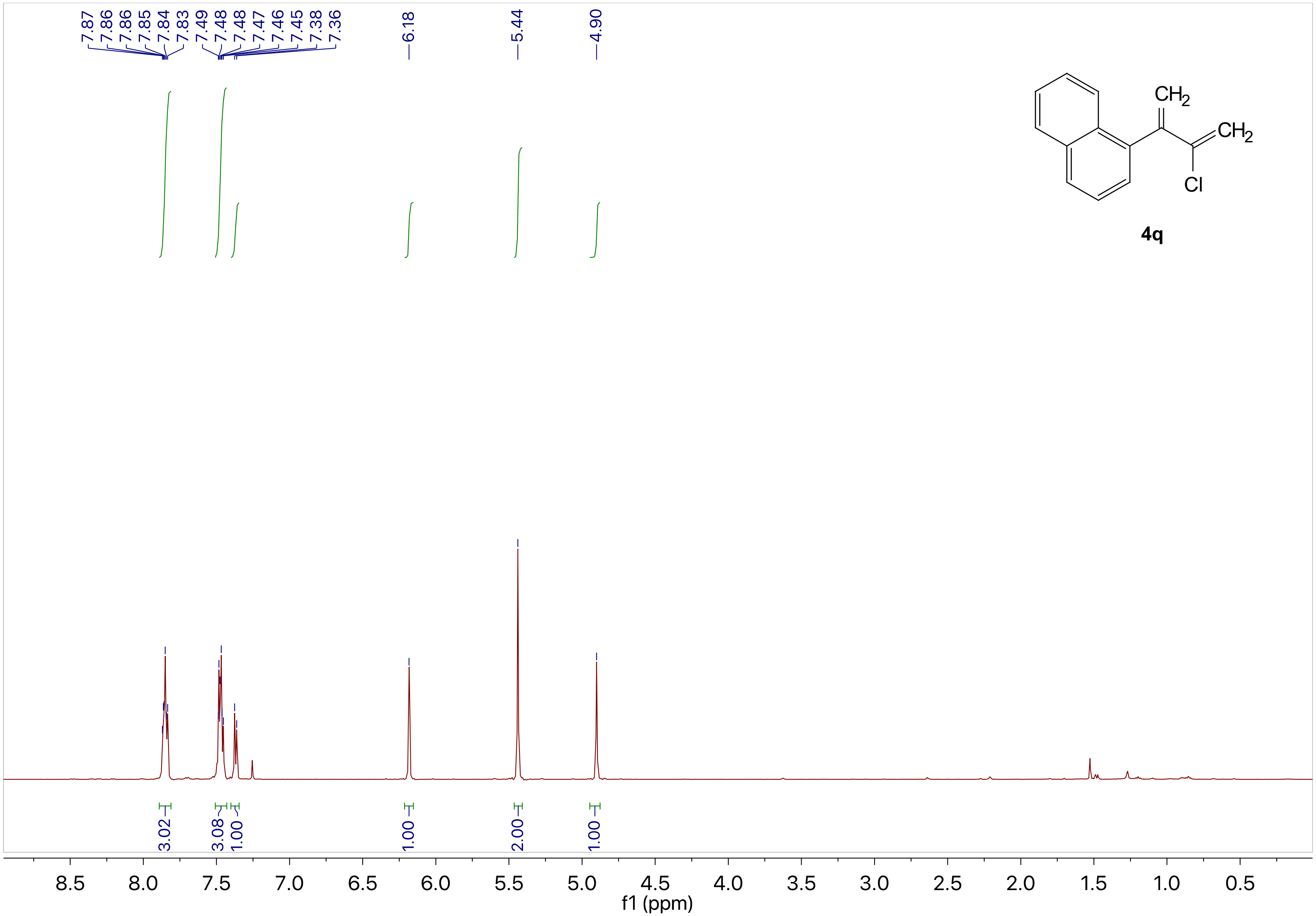
7.87
7.86
7.86
7.85
7.84
7.83
7.49
7.48
7.48
7.47
7.46
7.45
7.38
7.36
6.18
5.44
4.90
CH2
CH2
Cl
4q
3.02
3.08
1.00
1.00
2.00
1.00
8.5
8.0
7.5
7.0
6.5
6.0
5.5
5.0
4.5
4.0
3.5
3.0
2.5
2.0
1.5
1.0
0.5
f1 (ppm)

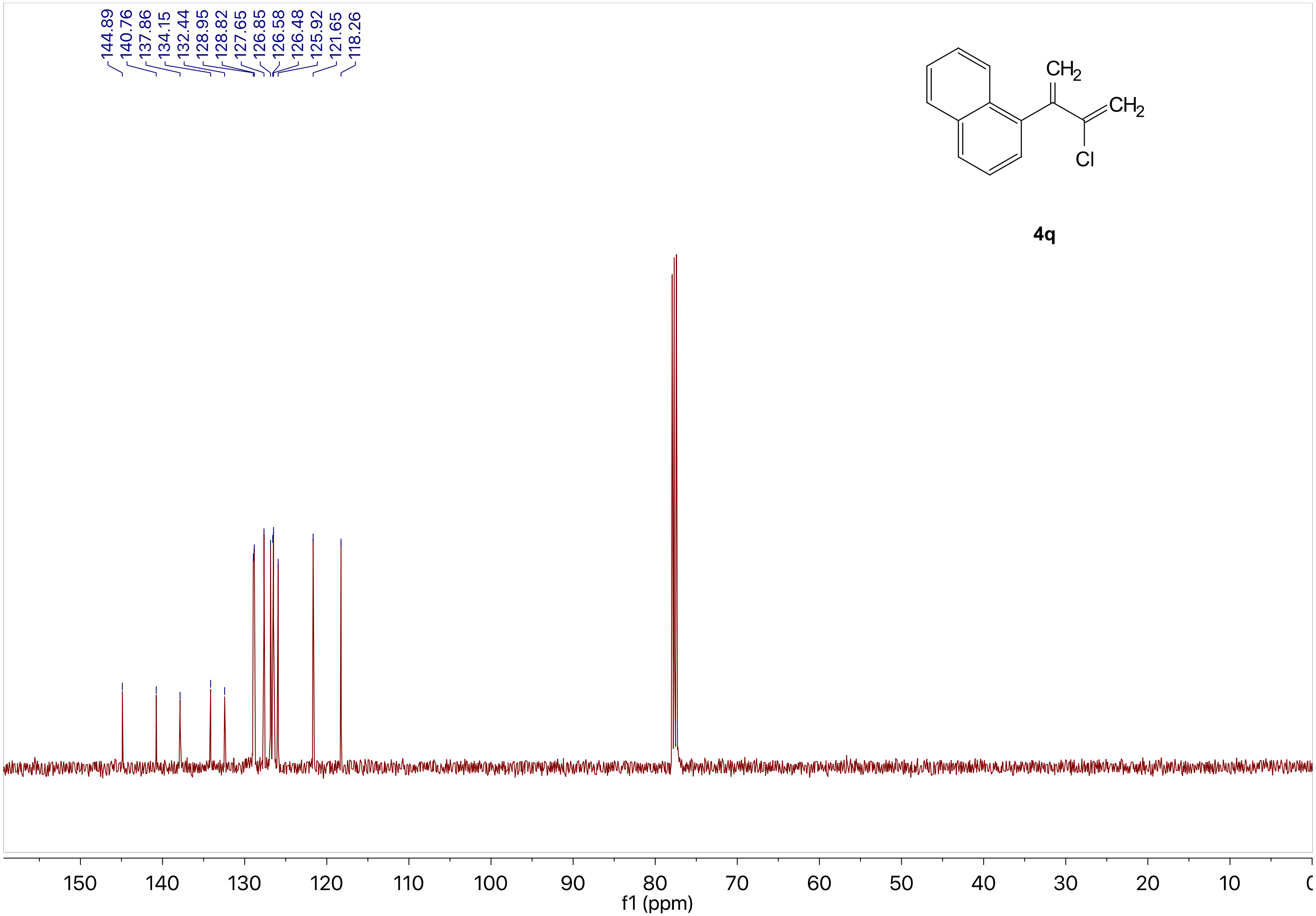
144.89
140.76
137.86
134.15
132.44
128.95
128.82
127.65
126.85
126.58
126.48
125.92
121.65
118.26
CH2
CH2
Cl
4q
150
140
130
120
110
100
90
80
70
60
50
40
30
20
10
f1 (ppm)

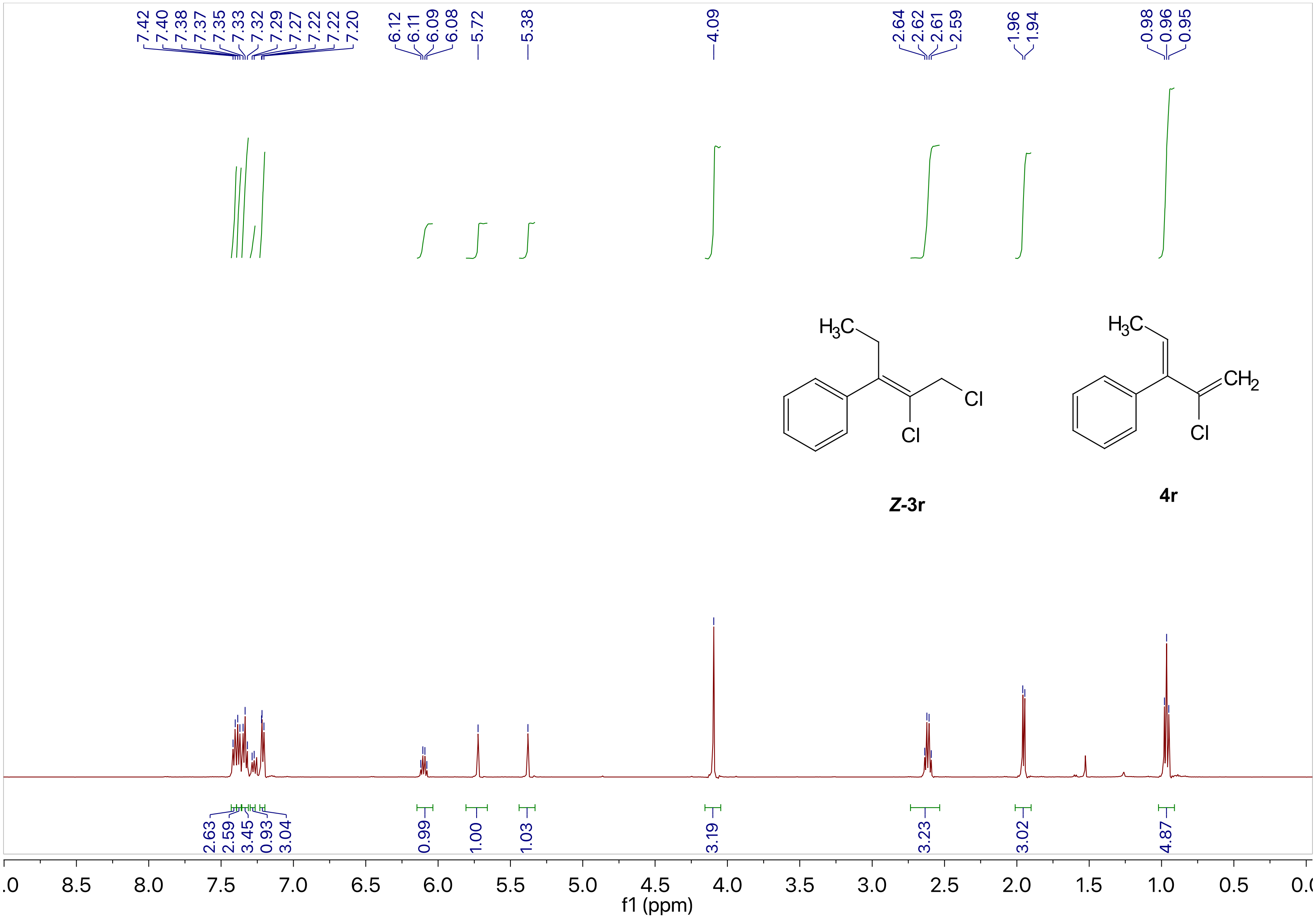
7.42
7.40
7.38
7.37
7.35
7.33
7.32
7.29
7.27
7.22
7.22
7.20
6.12
6.11
6.09
6.08
5.72
5.38
4.09
2.64
2.62
2.61
2.59
1.96
1.94
0.98
0.96
0.95
H3C
Cl
Cl
Z-3r
H3C
CH2
Cl
4r
2.63
2.59
3.45
0.93
3.04
0.99
1.00
1.03
3.19
3.23
3.02
4.87
f1 (ppm)

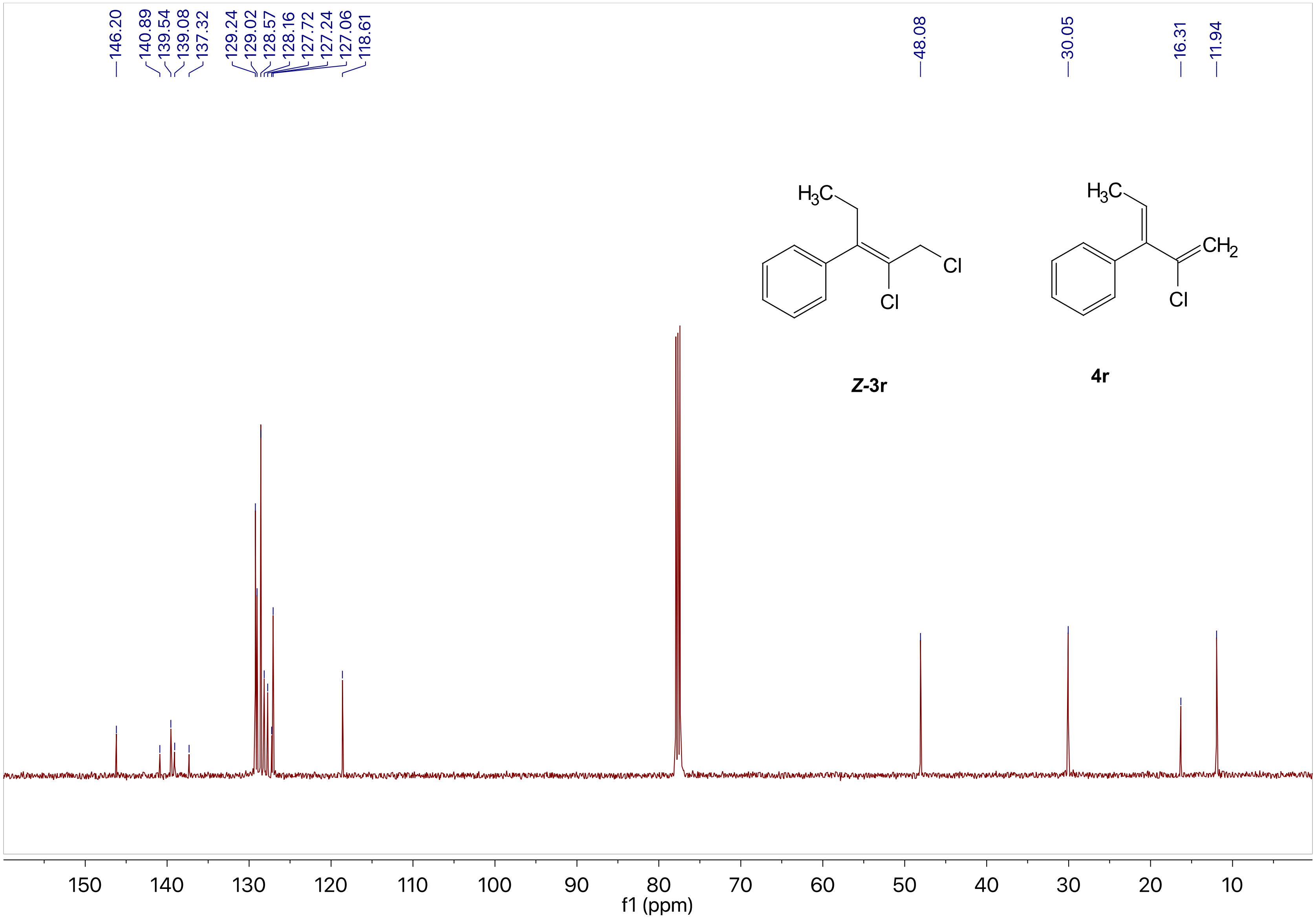
146.20
140.89
139.54
139.08
137.32
129.24
129.02
128.57
128.16
127.72
127.24
127.06
118.61
48.08
30.05
16.31
11.94
H3C
Cl
Cl
CH2
Z-3r
4r
150
140
130
120
110
100
90
80
70
60
50
40
30
20
10
f1 (ppm)

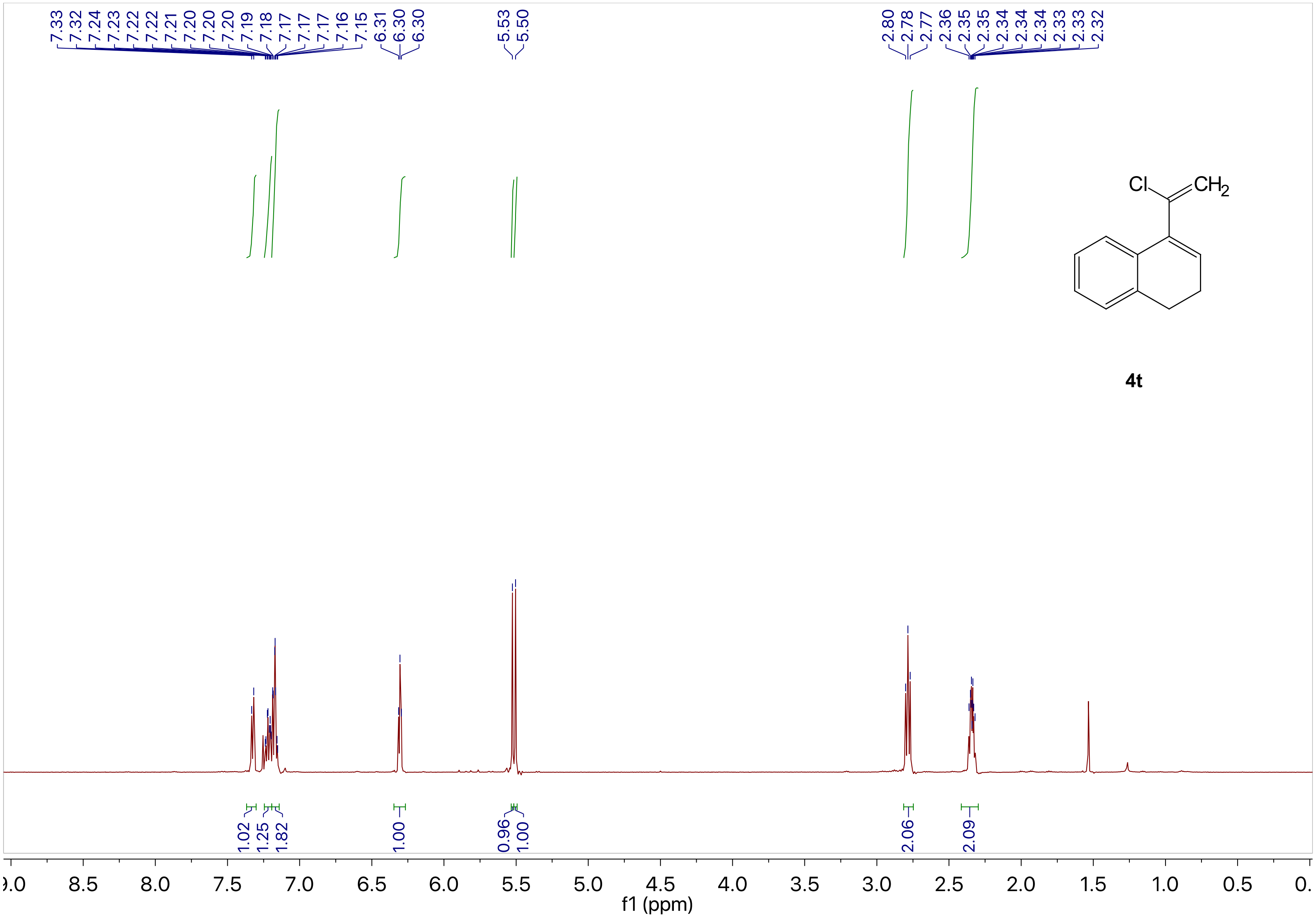
7.33
7.32
7.24
7.23
7.22
7.22
7.21
7.20
7.20
7.20
7.19
7.18
7.17
7.17
7.17
7.16
7.15
6.31
6.30
6.30
5.53
5.50
2.80
2.78
2.77
2.36
2.35
2.35
2.34
2.34
2.34
2.33
2.33
2.32
Cl
CH2
4t
1.02
1.25
1.82
1.00
0.96
1.00
2.06
2.09
f1 (ppm)

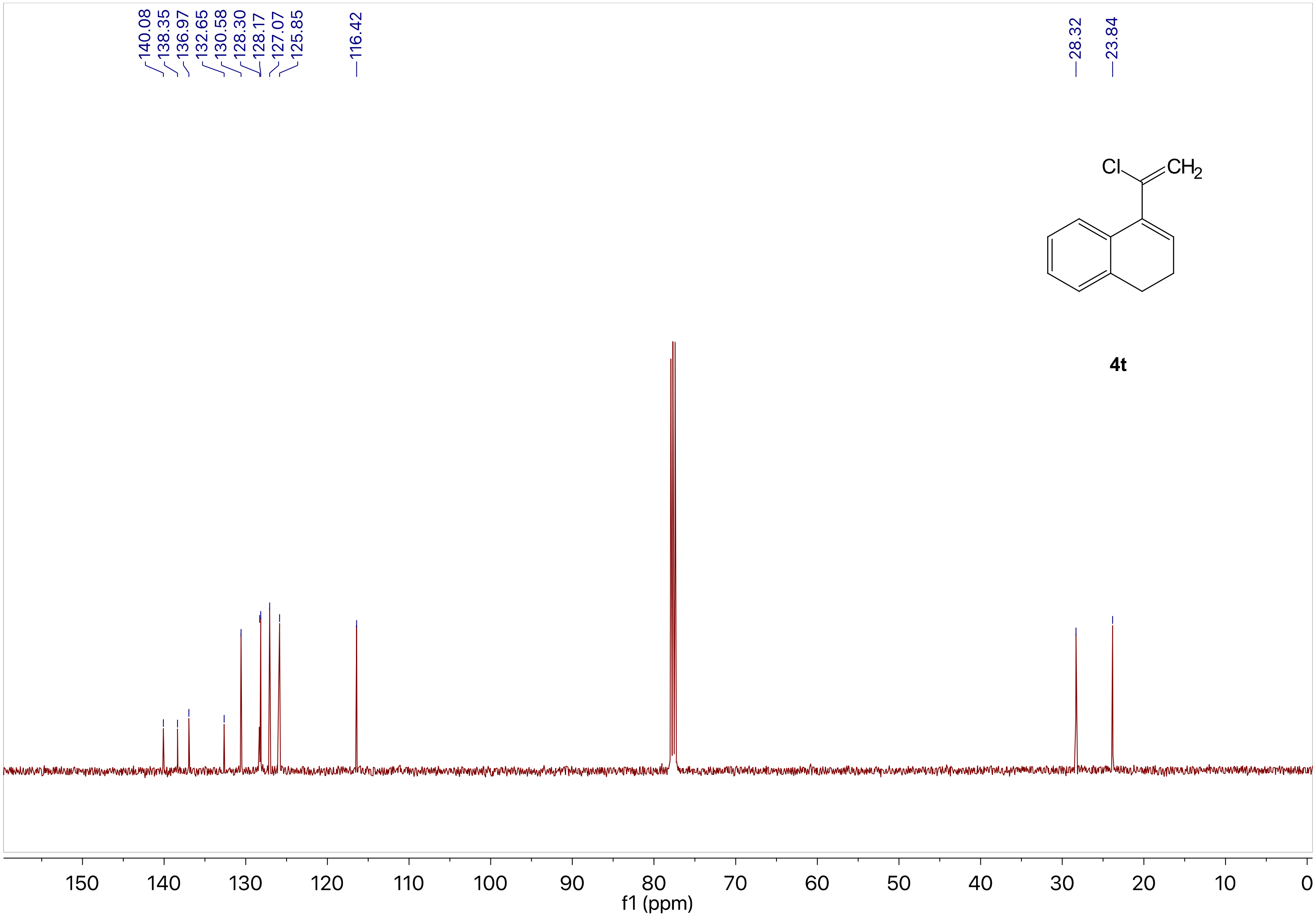

140.08
138.35
136.97
132.65
130.58
128.30
128.17
127.07
125.85
116.42
28.32
23.84
Cl
CH2
4t
150
140
130
120
110
100
90
80
70
60
50
40
30
20
10
0
f1 (ppm)

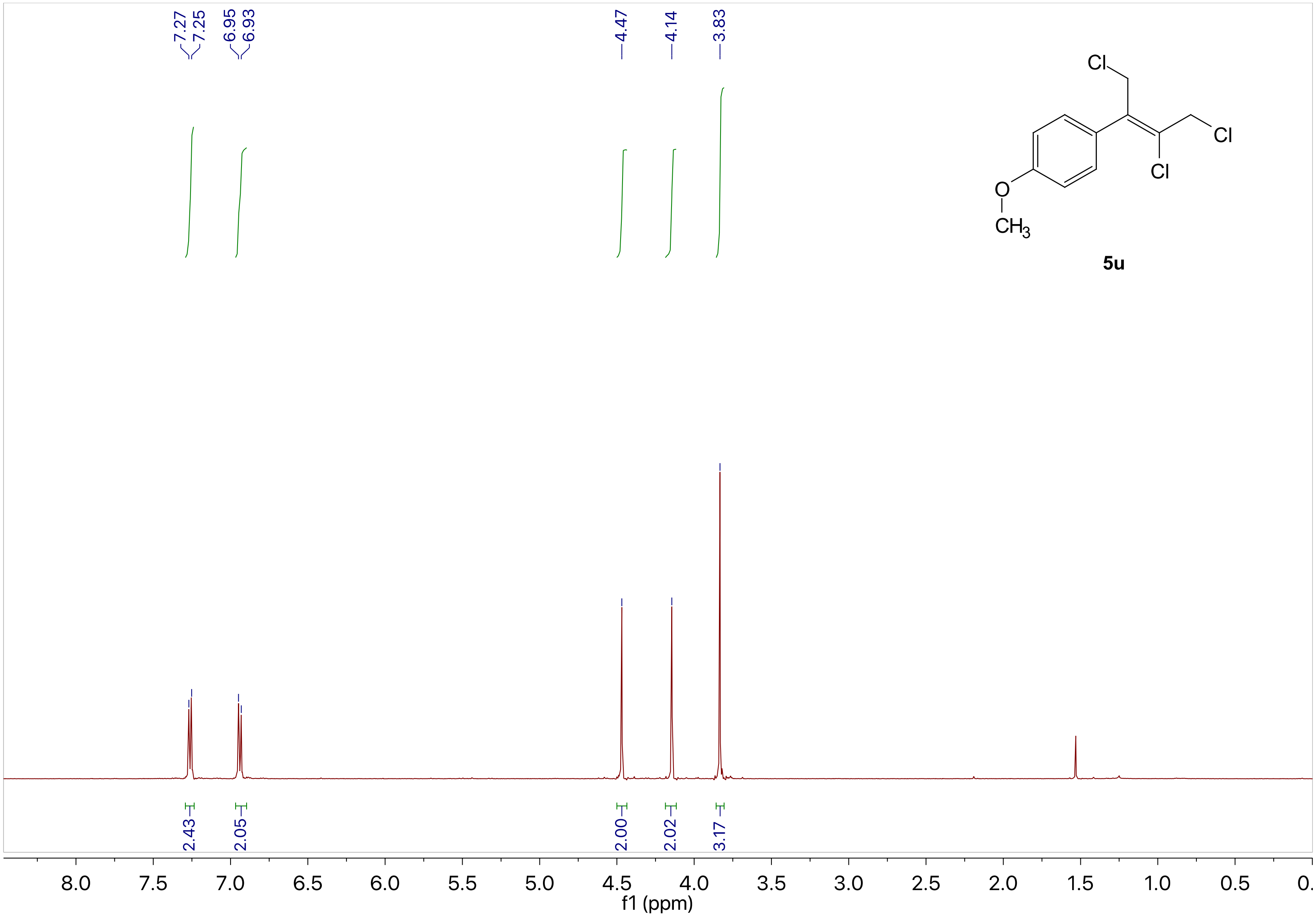

7.27
7.25
6.95
6.93
4.47
4.14
3.83
Cl
Cl
Cl
O
CH3
5u
2.43
2.05
2.00
2.02
3.17
8.0
7.5
7.0
6.5
6.0
5.5
5.0
4.5
4.0
3.5
3.0
2.5
2.0
1.5
1.0
0.5
0.
f1 (ppm)

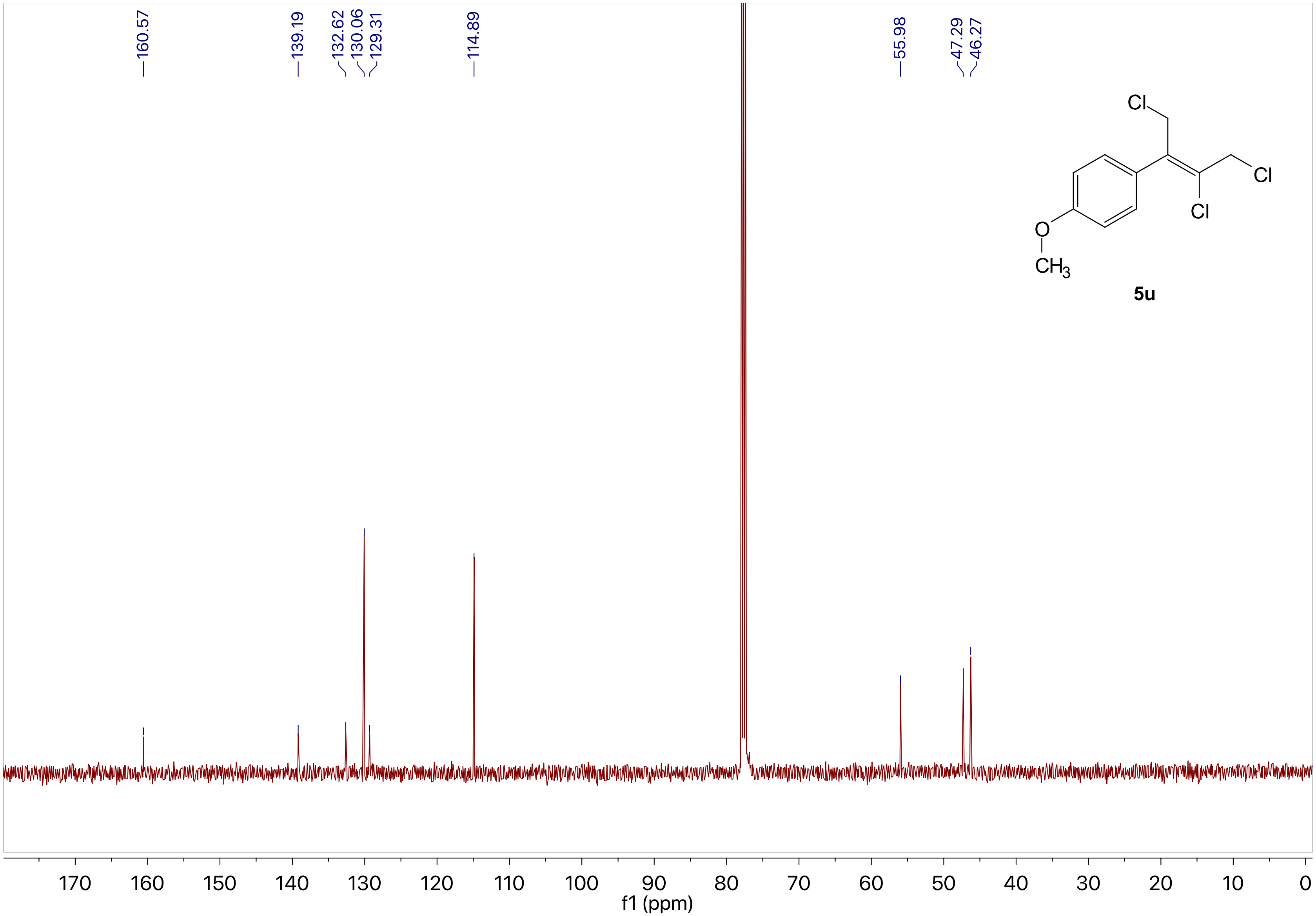
160.57
139.19
132.62
130.06
129.31
114.89
55.98
47.29
46.27
Cl
Cl
Cl
O
CH3
5u
170
160
150
140
130
120
110
100
90
80
70
60
50
40
30
20
10
0
f1 (ppm)

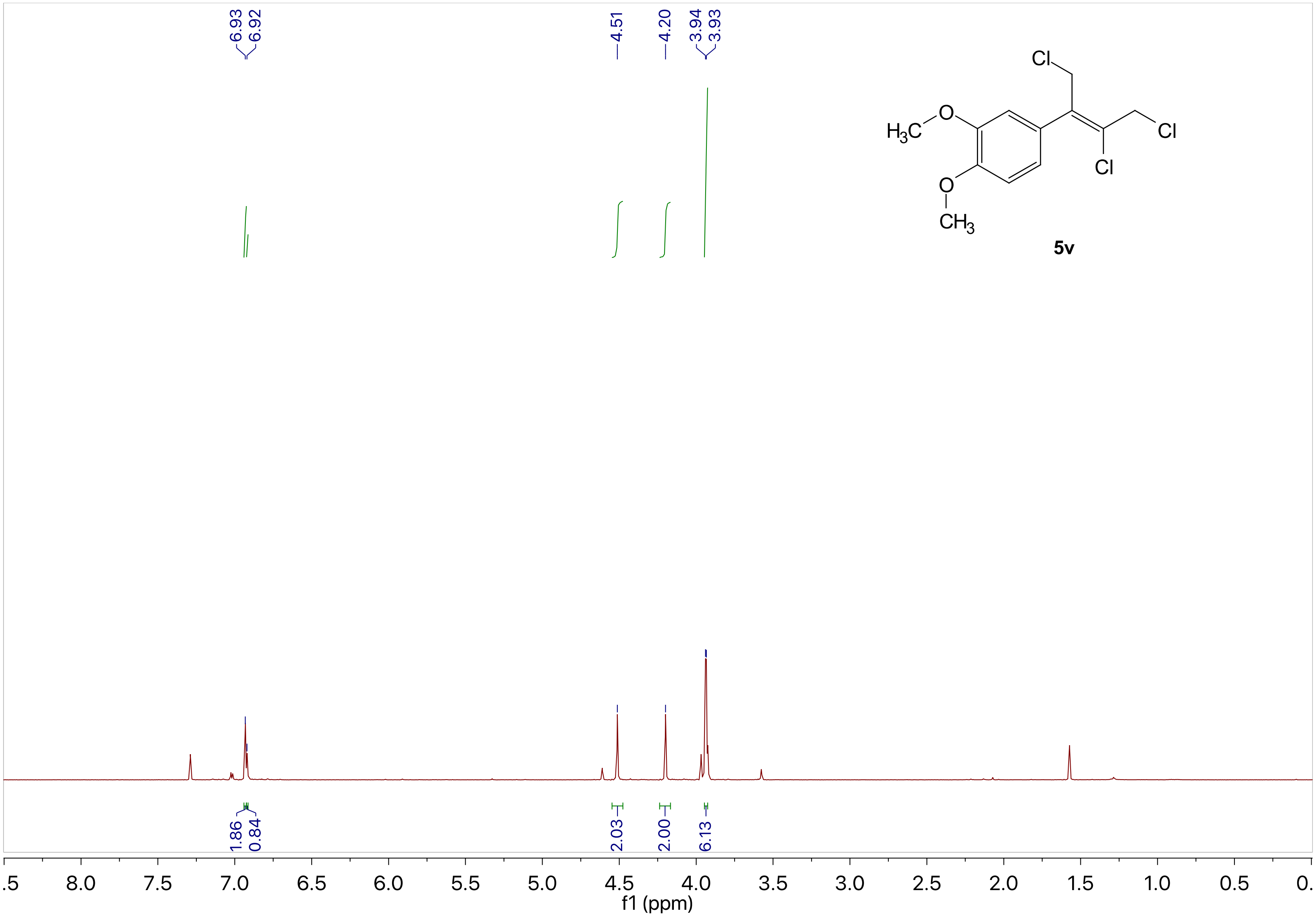
6.93
6.92
4.51
4.20
3.94
3.93
Cl
H3C
O
O
CH3
Cl
Cl
5v
1.86
0.84
2.03
2.00
6.13
.5
8.0
7.5
7.0
6.5
6.0
5.5
5.0
4.5
4.0
3.5
3.0
2.5
2.0
1.5
1.0
0.5
0.
f1 (ppm)

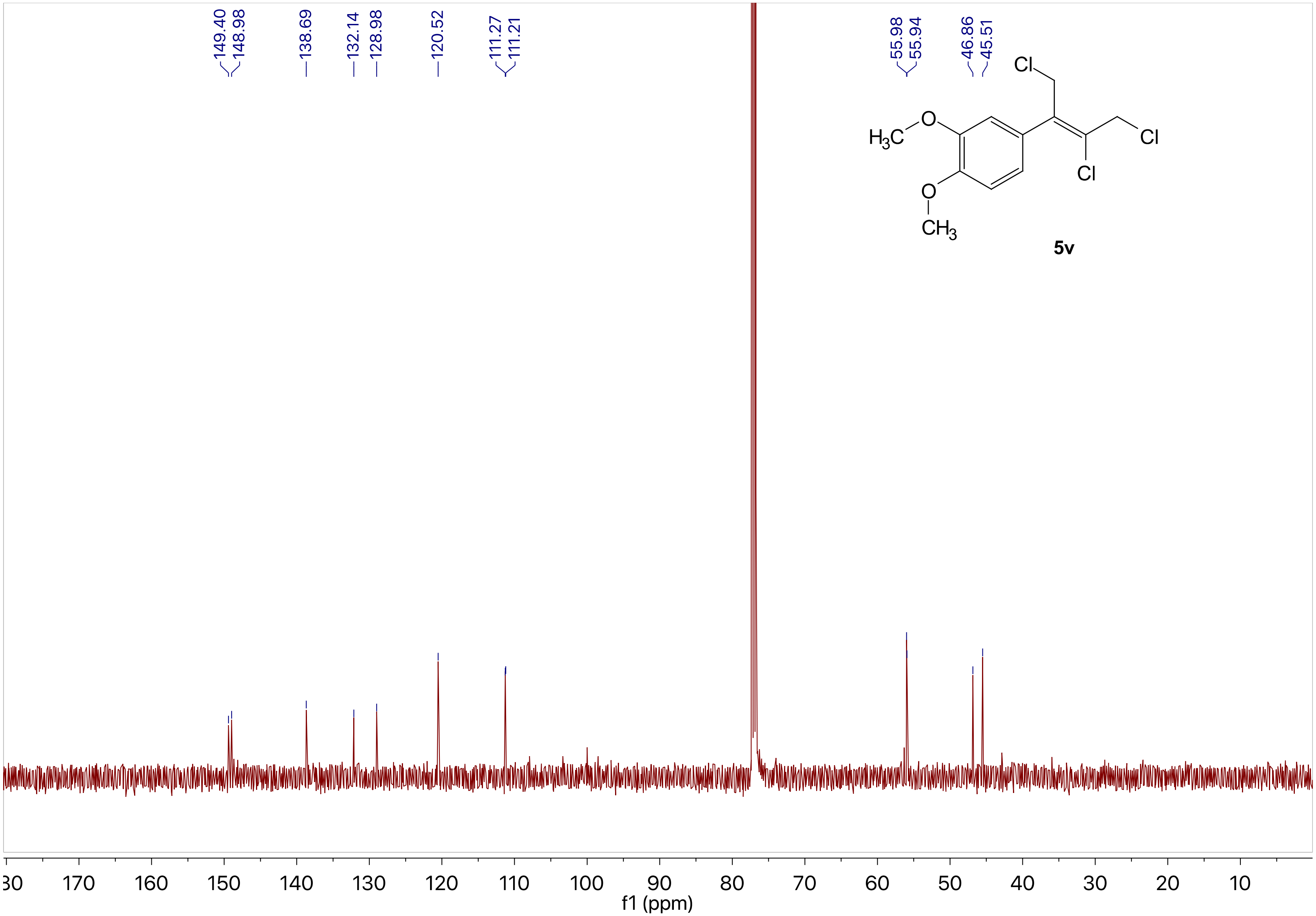
149.40
148.98
138.69
132.14
128.98
120.52
111.27
111.21
55.98
55.94
46.86
45.51
Cl
Cl
Cl
O
O
H_3C
CH_3
180
170
160
150
140
130
120
110
100
90
80
70
60
50
40
30
20
10
f1 (ppm)

5v

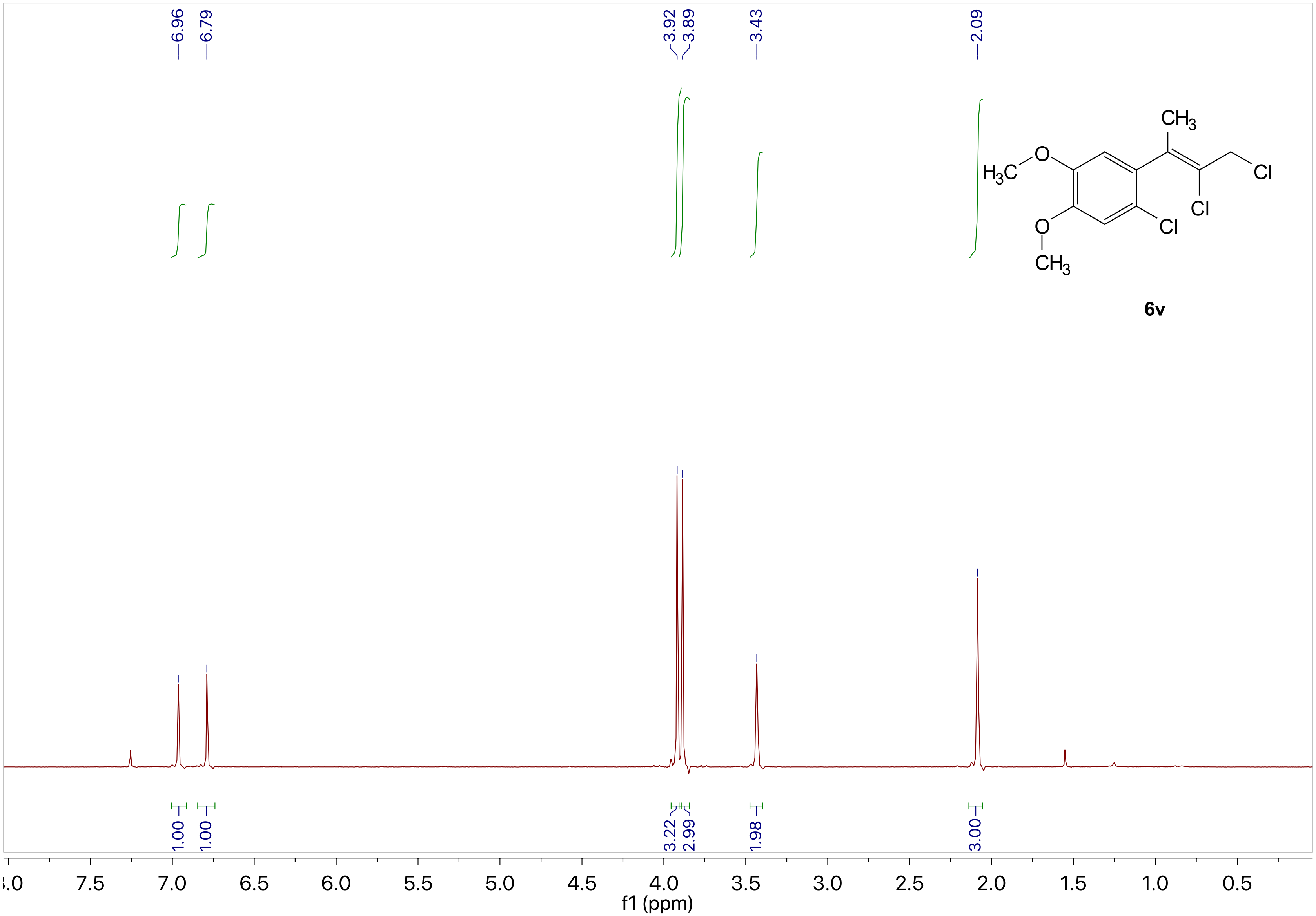
6.96
6.79
3.92
3.89
3.43
2.09
CH3
H3C
O
O
CH3
Cl
Cl
Cl
6v
1.00
1.00
3.22
2.99
1.98
3.00
7.5
7.0
6.5
6.0
5.5
5.0
4.5
4.0
3.5
3.0
2.5
2.0
1.5
1.0
0.5
f1 (ppm)

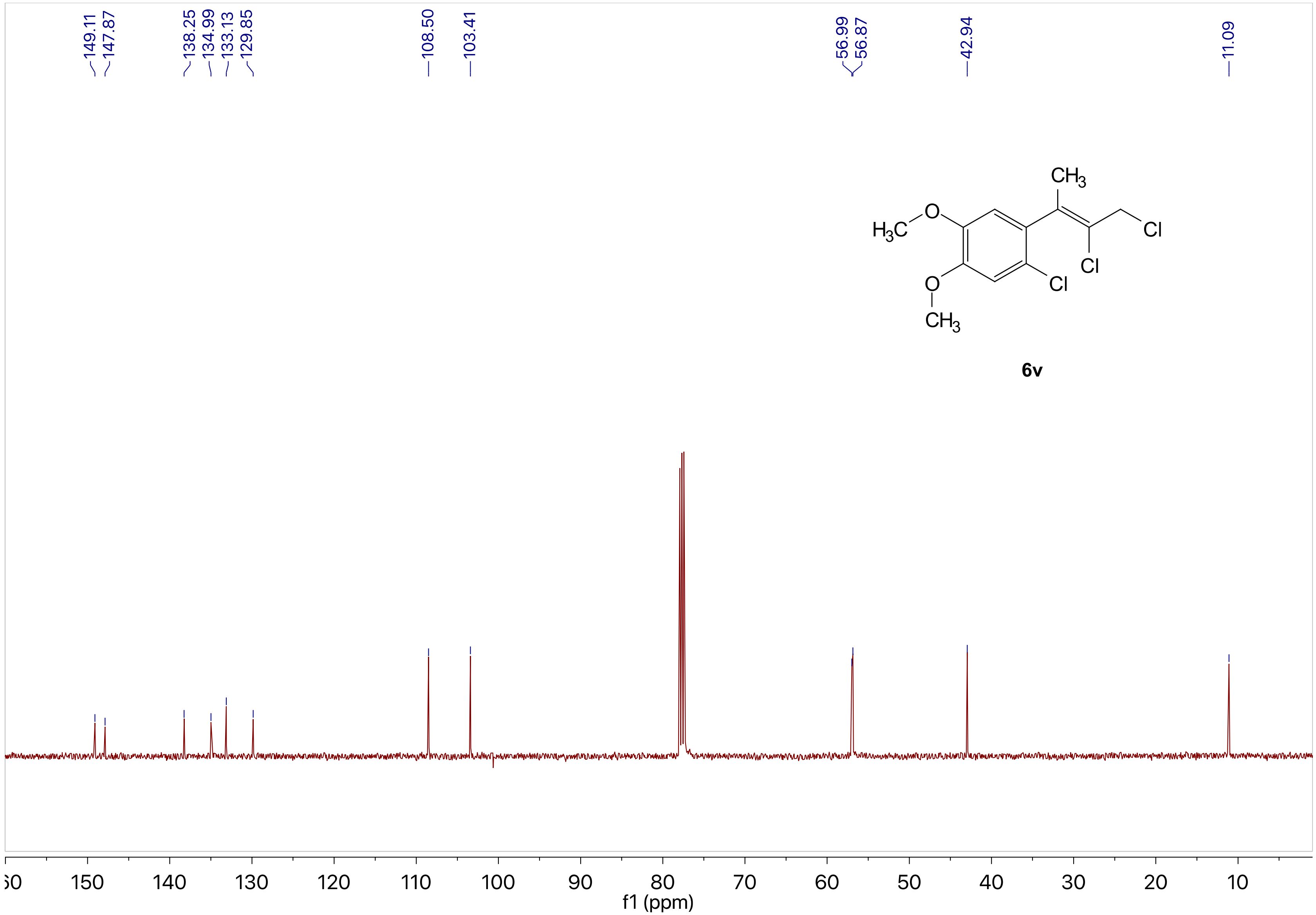
149.11
147.87
138.25
134.99
133.13
129.85
108.50
103.41
56.99
56.87
42.94
11.09
CH3
H3C
O
Cl
Cl
O
Cl
CH3
6v
150
140
130
120
110
100
90
80
70
60
50
40
30
20
10
f1 (ppm)

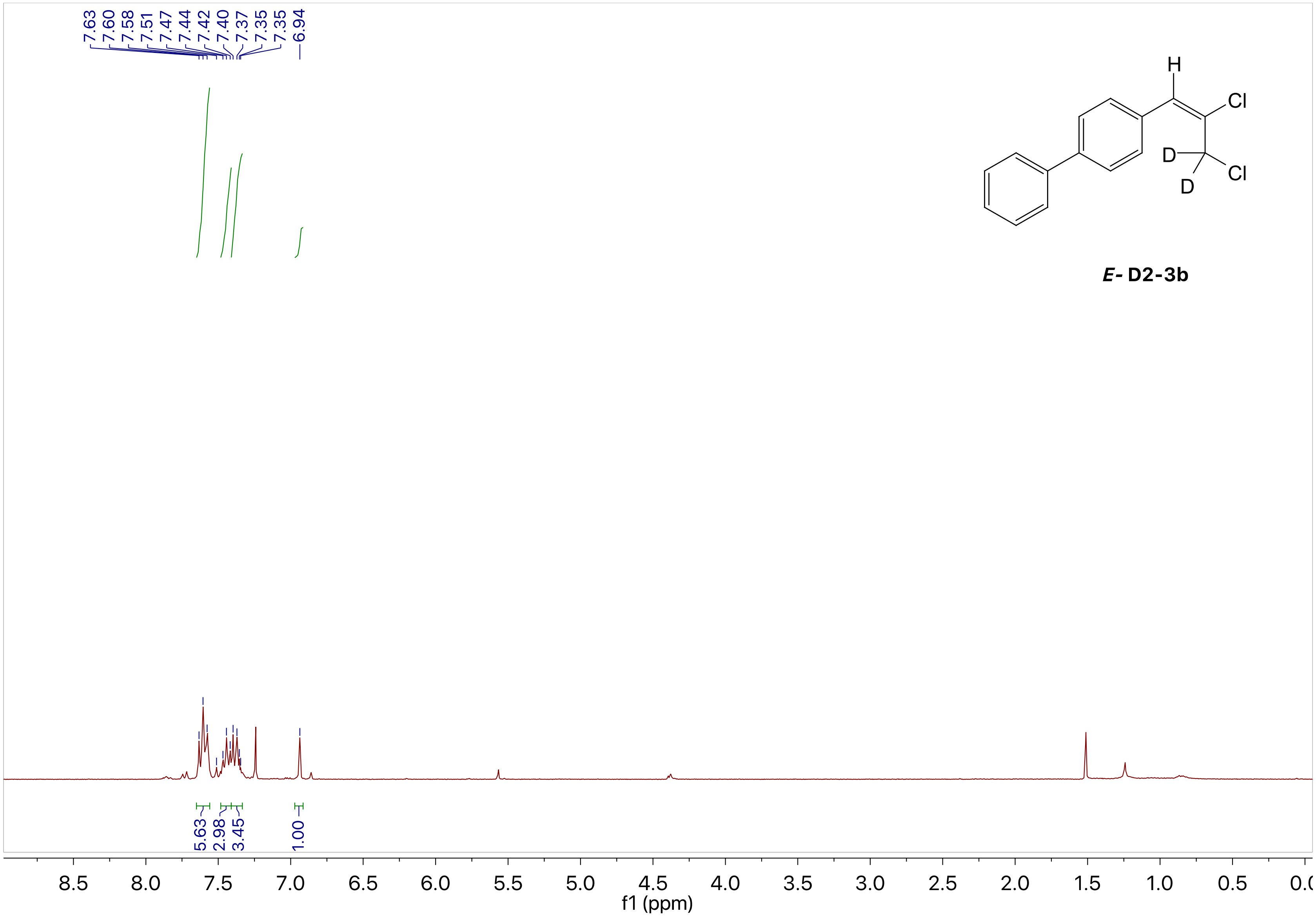
7.63
7.60
7.58
7.51
7.47
7.44
7.42
7.40
7.37
7.35
7.35
6.94
H
Cl
D
D
Cl
5.63
2.98
3.45
1.00
8.5
8.0
7.5
7.0
6.5
6.0
5.5
5.0
4.5
4.0
3.5
3.0
2.5
2.0
1.5
1.0
0.5
f1 (ppm)

***E*- D2-3b**

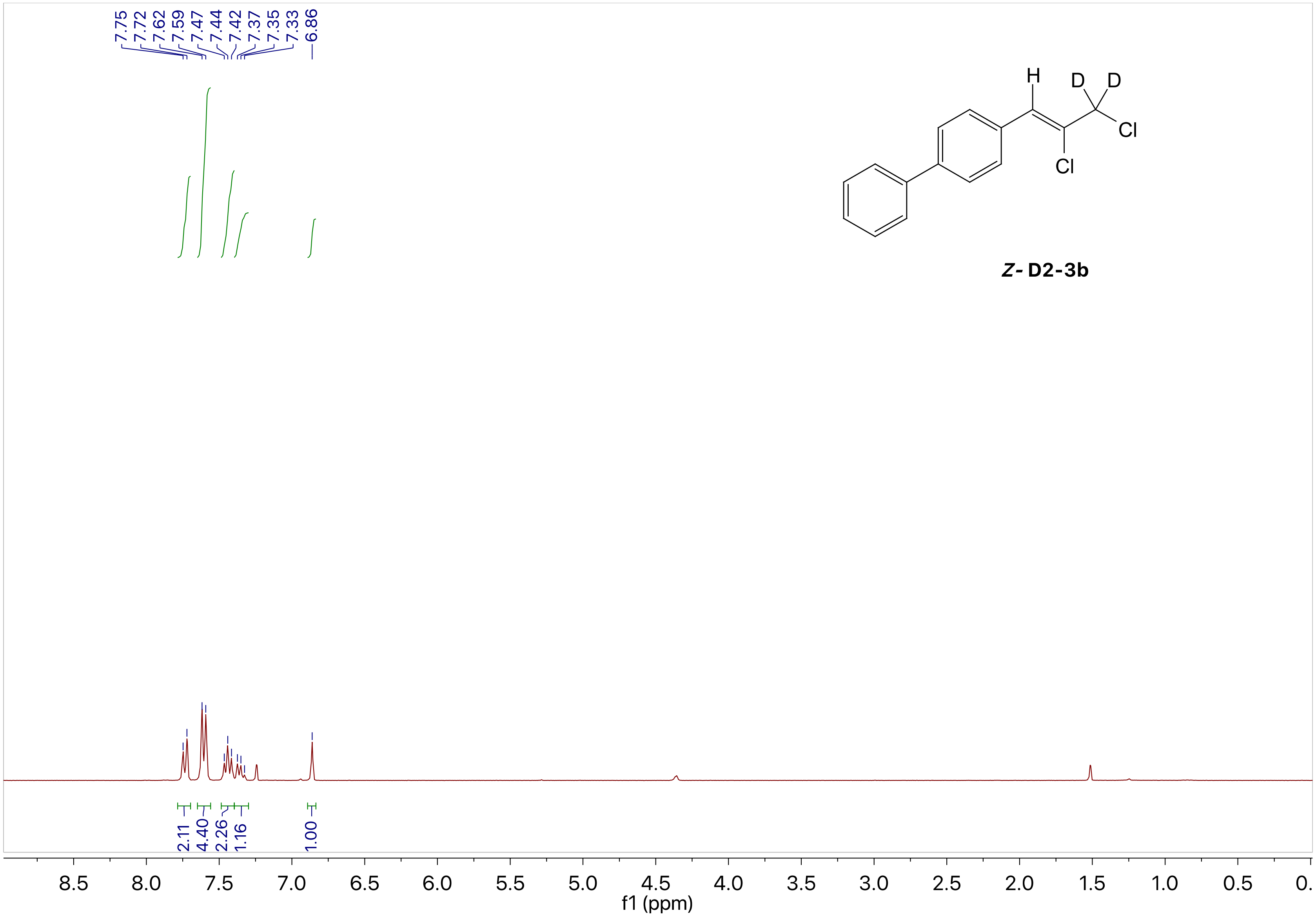
7.75
7.72
7.62
7.59
7.47
7.44
7.42
7.37
7.35
7.33
6.86
2.11
4.40
2.26
1.16
1.00
8.5
8.0
7.5
7.0
6.5
6.0
5.5
5.0
4.5
4.0
3.5
3.0
2.5
2.0
1.5
1.0
0.5
0.
f1 (ppm)
H
D
D
Cl
Cl

***Z*- D2-3b**
